# Association Between the *TP53* Polymorphisms and Breast Cancer Risk: An Updated Meta-Analysis

**DOI:** 10.3389/fgene.2022.807466

**Published:** 2022-04-27

**Authors:** Lin Zhao, Xiang-Xiongyi Yin, Jun Qin, Wei Wang, Xiao-Feng He

**Affiliations:** ^1^ Teaching Reform Class of 2018 of the First Clinical College, Changzhi Medical College, Changzhi, China; ^2^ Fifth Class of 2018 of the Second Clinical College, Changzhi Medical College, Changzhi, China; ^3^ General Surgery, Heping Hospital Affiliated to Changzhi Medical College, Changzhi, China; ^4^ Beijing Zhendong Guangming Pharmaceutical Research Institute, Beijing, China; ^5^ Institute of Evidence-Based Medicine, Heping Hospital Affiliated to Changzhi Medical College, Changzhi, China; ^6^ Department of Epidemiology, School of Public Health, Southern Medical University, Guangzhou, China

**Keywords:** TP53, polymorphism, breast, cancer, meta-analysis

## Abstract

**Background:** The relationship of *TP53* codons 72, IVS3 16 bp, and IVS6+62A > G polymorphisms with breast cancer (BC) risk has been analyzed in seventeen published meta-analyses. However, the credibility of statistically significant associations was ignored and many new studies have been reported on these themes.

**Objectives:** To explore whether *TP53* codons 72, IVS3 16 bp, and IVS6+62A > G polymorphisms are associated with BC risk and the clinical phenomena.

**Methods:** To comprehensively search the data (through October 25, 2021), we provided a clear search strategy and reviewed the references of published meta-analyses. The Preferred Reporting Items for Systematic Reviews and Meta-Analyses (PRISMA) were used.

**Results:** The current meta-analysis had a larger sample size than the previous ones: 99 studies with 43,951 BC and 48,479 controls for *TP53* codon 72 polymorphism, 35 studies with 8,705 BC and 7,516 controls for IVS3 16 bp polymorphism, and 25 studies with 12,222 BC and 12,895 controls for IVS6+62A > G polymorphism. Five gene models were used to explore the association between the three polymorphisms and BC risk, and partial positive results were similar to published meta-analyses results. However, a large number of significant results were considered to be unreliable after correcting with Bayesian false-discovery probability (BFDP), except for the association between *TP53* IVS3 16 bp polymorphism and BC risk in overall analysis (GG vs. CC: BFDP = 0.738), matched studies (GG vs. CC: BFDP = 0.173; GG vs. CC + CG: BFDP = 0.447), and tumor size below 2 cm (GG vs. CC: BFDP = 0.088; GG + CG vs. CC: BFDP = 0.730; GG vs. CC + CG: BFDP = 0.311). These unreliable results were confirmed again without new solid results emerging in further sensitivity analysis (only studies in compliance with the quality assessment standard).

**Conclusion:** After considering the quality of the included studies and the reliability of the results, the present meta-analysis suggested that *TP53* codons 72, IVS3 16 bp, and IVS6+62A > G polymorphisms were not significantly associated with the BC risk. Those results which prove that these three polymorphisms increase BC risk are more likely to be false-positive results due to various confounding factors.

## Introduction

According to the latest global cancer burden data released by the International Agency for Research on Cancer (IARC) of the World Health Organization, the number of new cases of BC in the world has surpassed lung cancer as the most commonly diagnosed cancer with 2.26 million (Sung et al., 2021) (https://gco.iarc.fr/today/home). Estrogen receptor (ER), progesterone receptor (PR), and human epidermal growth factor receptor 2 (HER2) are currently recognized as biomarkers affecting treatment decisions in BC patients (Voduc et al., 2010; El-Deiry et al., 2019). These markers can be used to predict BC response to treatment and to guide treatment plans. Determining ER, PR, and HER2 status was recommended for all newly diagnosed invasive BCs and any recurrence when feasible (Zardavas et al., 2015; Du et al., 2021). Some personal habits and environmental factors have been shown to be associated with the initiation of BC, such as smoking and radiation-related work (Jagsi, 2014; Siegel et al., 2021). But not all women who are exposed to these risk factors will develop BC. Therefore, there must be some intrinsic factors that make the women more likely to develop BC, such as specific genetic polymorphisms (Fachal et al., 2020).

Owing to its important role in cell mutation, tumor-suppressor gene *TP53* located on 17p13 is a persistent research hotspot in the field of life science (Fukasawa et al., 1997; Negrini et al., 2010; Levine, 2020). In general, according to the latest research, the function of *TP53* has been proved to involve in the regulation of almost all cellular biological processes, including cell apoptosis, aging, differentiation, migration, metabolism, autophagy, and so on (Mercer et al., 1982; Donehower, 2009; Oren and Rotter, 2010; Kastenhuber and Lowe, 2017; Mantovani et al., 2019). However, *TP53* is often inactivated by missense mutations in the DNA-binding domain. When *TP53* gene is mutated, due to spatial conformation changed, *TP53* not only loses tumor suppression functions, but also promotes cancer (Hingorani et al., 2005; Kim and Lozano, 2018). In a mutation study of 560 Malaysian breast tumors, compared with breast tumors in Caucasian women, they found that ER+ Asian breast tumors had a higher prevalence of *TP53* somatic mutations and Asian women showed an increased prevalence of HER2-positive molecular subtypes (Pan et al., 2020). HER2 subtypes and abundant immune scores were associated with improved survival, whereas the presence of *TP53* somatic mutations was associated with worser survival in ER+ tumors (Pan et al., 2020). A new study found that wild-type *TP53* and mutant *TP53* completely played opposite roles in the cGAS-STING pathway, and the mutant *TP53* promoted the initiation of tumors by inhibiting innate immune signaling pathways, while inhibiting immune surveillance function (Ghosh et al., 2021). They provide a novel molecular mechanism of mutant *TP53* in the cancer-promoting and a new potential therapeutic target for BC associated with mutant *TP53*.

Since single-nucleotide polymorphisms (SNPs) of tumor-suppressor gene *TP53* are proved to relate to the susceptibility of BC (Shiovitz and Korde, 2015), exploring the distribution of *TP53* genotypes in the population becomes a helpful procedure to provide a reference for the prediction of BC. Therefore, we selected three well-characterized SNPs in the *TP53* gene at codon 72 of exon 4 (rs1042522), IVS3 16 bp (rs17878362), and IVS6+62A > G (rs1625895) for this study. Codon 72 polymorphism is the variant encoding a proline rather than an arginine residue (Matlashewski et al., 1987), IVS3 16 bp is an insertion polymorphism between a 16-base pair (bp) in intron 3 (Lazar et al., 1993; Runnebaum et al., 1994), and IVS6+62A > G is a SNP, a CCGG to CCAG transition, located in 61 bp of intron 6 downstream of exon 6 (Chumakov and Jenkins, 1991). Although these three polymorphisms have been reported by 108 studies, the relationship between the three SNPs and BC risk remains unclear, because of small sample size and contradictory results. Furthermore, seventeen meta-analyses (Dunning et al., 1999; Suspitsin et al., 2003; Zhuo et al., 2009; Hu et al., 2010a; Hu et al., 2010b; Zhang et al., 2010; Francisco et al., 2011; He et al., 2011; Ma et al., 2011; Cheng et al., 2012; Dahabreh et al., 2013; Hou et al., 2013; Sagne et al., 2013; Wu et al., 2013; Gonçalves et al., 2014; Diakite et al., 2020a; Diakite et al., 2020b) have been published and tried to describe the connection between *TP53* codons 72, IVS3 16 bp, and IVS6+62A > G polymorphisms and BC risk, but their results tended to be contradictory and heterogeneous. Moreover, *TP53* polymorphisms based on patient clinical characteristics were not considered in all these published meta-analyses. Therefore, based on the previous meta-analysis studies and to make the final results more robust, we performed an updated systematic review and meta-analysis to address the above issues by improving data collection and evaluation methods and refining statistical evaluation methods.

## Materials and Methods

All processes for the current systematic review and meta-analysis were based on the PRISMA guidelines.

### Search Strategy

We conducted a systematic literature search using PubMed, China National Knowledge Infrastructure (CNKI), and Wanfang Database. Combinations of the following keywords and their synonyms were used: “*TP53*,” “polymorphism, genetic,” “breast,” “arg72pro,” “IVS3 16 bp,” “IVS6+62A>G.” The full search strategy was available in Supplementary Appendix. In addition, two *TP53*-specific databases were also used: the International Agency for Research on Cancer *TP53* database (Hainaut et al., 1998; Hainaut et al., 1997) (http://www-p53.iarc.fr/) and the p53 website (Béroud and Soussi, 2003; Hamroun et al., 2006) (http://p53.free.fr/), and the search process for both databases followed the annotated bibliography lists provided by the database itself. There were no language restrictions for eligible studies here. Meanwhile, additional studies were screened out from the references of published reviews and meta-analyses. All qualified studies were determined by reading the title, abstract, and full text of the literature. In addition, if necessary, we contacted the corresponding authors for more information by e-mail. All searches were updated to October 25, 2021.

### Inclusion and Exclusion Criteria

Studies were included if only they met the following criteria: 1) case-control, nested case-control, or cohort studies; 2) genotype frequency or odds ratios (ORs) and 95% confidence intervals (CIs) were available; 3) studies discussed the association between *TP53* codon 72 (rs1042522), IVS3 16 bp (rs17878362), or IVS6+62A > G (rs1625895) polymorphisms and BC risk; 4) studies population were female.

In the case of multiple overlapping research populations, we only included the latest study with the largest sample in the present analysis, and excluded data from other overlapping reports. Overlapping characteristics of study populations were determined by comparing authors, study centers, demographic characteristics, and recruitment periods. We did not consider the studies that the population was a mix of men and women. And studies with significant heterogeneity, such as cases and controls that were not in the same continent, were excluded. Eventually, editorials, narrative reviews, or other manuscripts that do not report major research findings were excluded.

### Data Extraction and Quality Assessment

Based on the inclusion and exclusion criteria listed above, the two authors (Zhao and Yin) independently screened the abstracts and full texts and carefully extracted information from all the eligible studies searched. The following data were collected from each study: first author, year of publication, sample country, geographic region and ethnicity, sample size, source of controls, source of cases, with what technique to ascertain cancers and controls, source of *TP53* genotyping material of case (tumor tissue or other DNA sources), type of controls, whether cases and controls were matched (case-control studies), whether genotyping was done blindly and/or quality controlled, whether the association between genotypes and BC was assessed with appropriate statistics and adjustment for confounders, and genotype distribution.

The quality scores of the included studies were evaluated respectively by two authors (Zhao and Yin) following the Supplementary Table S1 criteria. These assessment criteria were applied based on a comprehensive consideration for the quality of the characters of the included researches (e.g., HWE, source of participants, matching of controls, sample size, research method, and so on), which had been used by the previous studies (Klug et al., 2009; Thakkinstian et al., 2011). In the control group, the goodness-of-fit chi-square test was used to test the Hardy-Weinberg equilibrium (HWE) of every study with complete genotype data. Significant bias was considered if the *p-*value was less than 0.05 (Thakkinstian et al., 2005). The highest score was 23, and a study that met both scoring >16, 16 and HWE compliant was regarded as high-quality. Similarly, the disagreement in scores was assessed by a superior author.

### Statistical Analysis

Crude ORs and 95% CIs were pooled to evaluate the association between *TP53* codon 72, IVS3 16 bp and IVS6+62A > G polymorphisms, and the susceptibility of BC. We performed ORs with the corresponding 95% CIs following five genetic models: 1) heterozygote model (CG vs. CC), 2) homozygote model (GG vs. CC), 3) dominant model (CG + GG vs. CC), 4) recessive model (GG vs. CC + CG), and 5) allele model (G vs. C). The C allele was the major or wild allele, and the G allele was the rare or mutant allele.

The heterogeneity test was carried out based on the chi-square *Q*-test (Davey Smith and Egger, 1997) and *I* ^*2*^ test (Higgins and Thompson, 2002; Higgins et al., 2003). The result was interpreted as no obvious heterogeneity if *p* > 0.10 and *I* ^*2*^ ≤ 50%, and a fixed-effects model was used (Mantel and Haenszel, 1959). Otherwise, we applied a random-effects model (DerSimonian and Laird, 1986; Higgins et al., 2009). If a significant heterogeneity was present, we might apply a meta-regression analysis to search for the sources of heterogeneity. Moreover, subgroup analyses were performed by stratifying studies with following characteristics: matching of controls, blindly and/or quality control genotyping, HWE condition, source of controls, geographic region, and ethnicity. According to the scoring result and the HWE condition, we performed a sensitivity analysis with only high-quality (>16,16) and fit HWE research. To evaluate the publication bias, we carried out Begg’s rank correlation test, Begg’s funnel plot (Begg and Mazumdar, 1994), and Egger’s linear regression test (Egger et al., 1997). The result was considered no obvious publication bias when the quantitative method showed Begg’s and Egger’s test (*p* > 0.05), and Begg’s funnel plots were symmetrical by visualizing. If a significant publication bias existed, we might use the nonparametric “trim and fill” method to correct and identify funnel plot asymmetry caused by publication bias, and meanwhile estimate the true value of the quantitative synthesis (Duval and Tweedie, 2000). Additionally, the BFDP, a further credibility calculation, was used to evaluate the credibility of significant results in the present analysis. As long as the BFDP value proved under 0.8 with a prior probability of 0.001 (Wakefield, 2007), the credibility of positive results was considered to be affirmative. All statistical analyses in the current study were performed by using Stata version 12.0 (Stata Corporation, College Station, TX, United States).

## Results

### Study Characteristics

**FIGURE 1:**
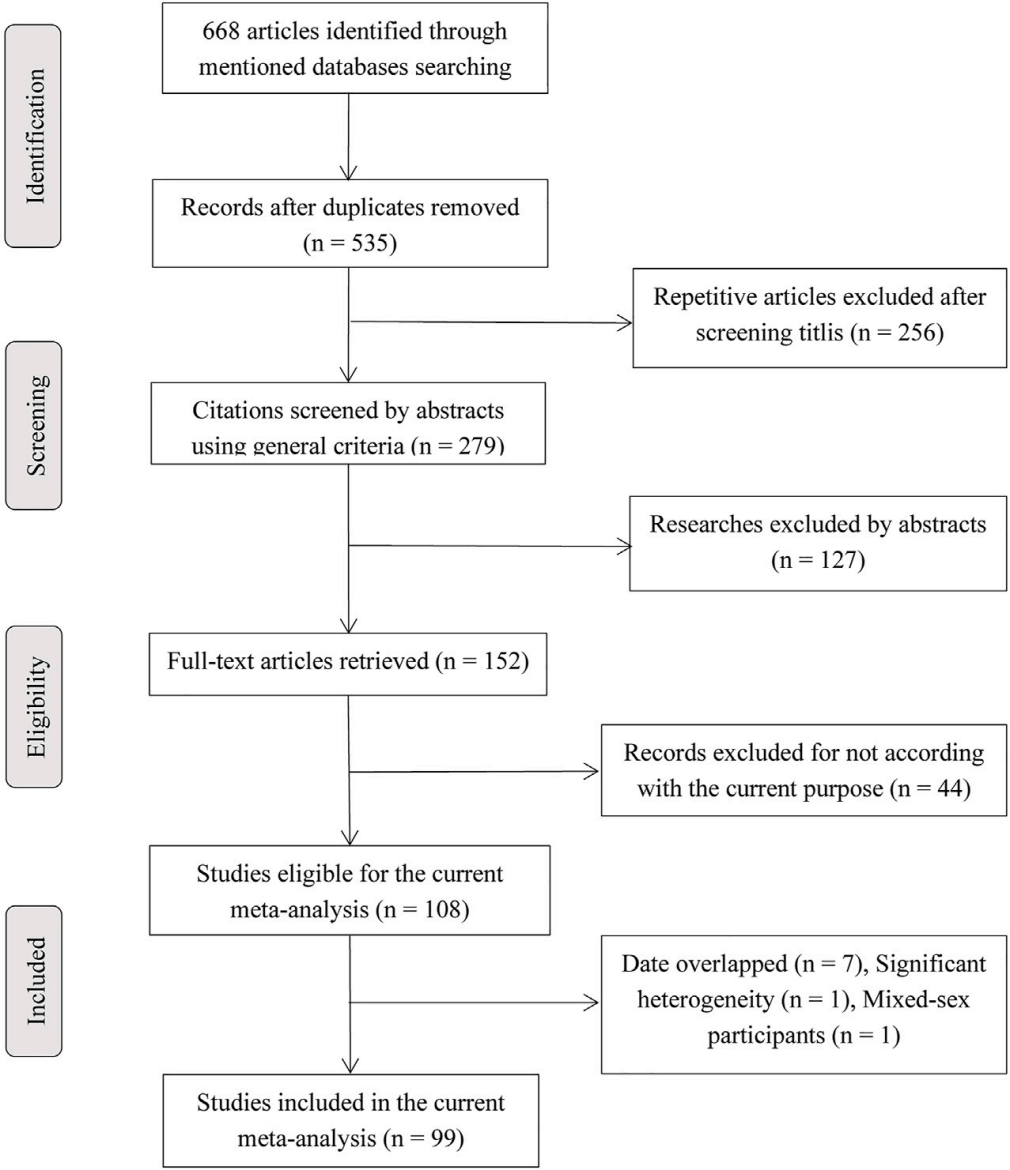
Flow diagram for searching included articles.

Figure 1 showed a detailed flow diagram for identifying and incorporating studies. In summary, a total of 108 articles (see Supplementary Appendix) were eligible for the current study. Then, 11 studies were excluded because their data were redundant with other 9 studies. The details of the redundancy are shown in Supplementary Appendix. One study (Siddique et al., 2005) was excluded because cases and controls were not from the same continent, which may lead to significant heterogeneity, and one study (Syeed et al., 2010) was excluded because of mixed gender participants. Finally, 99 articles were involved in this analysis. Of these, because several articles reported more than one locus and study, 99 studies (including 43,951 BC cases and 48,479 controls) described *TP53* codon 72 polymorphism, 35 studies (including 8,705 BC cases and 7,516 controls) on IVS3 16 bp polymorphism, and 25 studies (including 12,222 BC cases and 12,895 controls) belong to IVS6+62A > G polymorphism. Supplementary Table S6 lists the detailed characteristics of the included studies and Supplementary Table S7 displays the data of *TP53* polymorphisms based on the clinicopathological characteristics of BC, and the cells with red color indicated HWE in violation in these tables.

According to the evaluation criteria in Supplementary Table S1, we sorted the concise characteristics of the included studies with respect to these three polymorphisms (Table 1). As is shown in Table 1, studies with relatively high-quality characteristics accounted for more than 60% of the total included studies. However, the proportion of matched studies and blindly and/or quality-controlled genotyping studies were relatively low, especially those involved in *TP53* codon 72 and IVS3 16 bp polymorphisms.

**TABLE 1:** Concise characterization of the studies included from 99 articles in present meta-analysis.

	n		
	codon 72 (rs1042522) total 99	IVS3 16bp (rs17878362) total 35	IVS6+62A > G (rs1625895) total 25
Source of case			
Selected from population or cancer registry	27 (27.3%)	10 (28.6%)	11 (44.0%)
Selected from hospital	56 (56.6%)	21 (60.0%)	10 (40.0%)
Selected from pathology archives, but without description	5 (5.1%)	1 (2.7)	1 (4.0%)
Not described	11 (11.1%)	3 (8.6%)	3 (12.0%)
Source of control			
Population-based	37 (37.4%)	16 (45.7%)	13 (52.0%)
Blood donors or volunteers	20 (20.2%)	9 (25.7%)	4 (16.0%)
Hospital-based	18 (18.2%)	4 (11.4%)	3 (12.0%)
Not described	24 (24.2%)	6 (17.1%)	5 (20.0%)
Ascertainment of cancer			
Histological or pathological confirmation	54 (54.5%)	23 (65.7%)	15 (60.0%)
Diagnosis of BC by patient medical record	29 (29.3%)	8 (22.9%)	8 (32.0%)
Not described	16 (16.2%)	4 (11.4%)	2 (8.0%)
Ascertainment of control			
Controls were tested to screen out BC	11 (11.1%)	2 (5.7%)	0 (0.0%)
Controls were subjects who did not report BC, no objective testing	82 (82.8%)	29 (82.9%)	20 (88.0%)
Not described	6 (6.1%)	4 (11.4%)	5 (20.0%)
Matching			
Controls matched with cases by age	43 (43.4%)	18 (51.4%)	13 (52.0%)
Not matched or not described	56 (56.6%)	17 (48.6%)	12 (48.0%)
Source of genotyping material of case			
Appropriate DNA sources (such as peripheral blood, buccal swabs, saliva, and so on)	77 (77.8%)	29 (82.9%)	21 (84.0%)
Tumor tissue	11 (11.1%)	4 (11.4%)	2 (8.0%)
Not described	11 (11.1%)	2 (5.7%)	2 (8.0%)
Genotyping examination			
Genotyping done blindly and quality control	13 (13.1%)	5 (14.3%)	6 (24.0%)
Only genotyping done blindly or quality control	24 (24.2%)	6 (17.1%)	5 (20.0%)
Unblinded and without quality control	62 (62.6%)	24 (68.6%)	14 (56.0%)
HWE			
HWE in the control group	79 (79.8%)	31 (88.6%)	21 (84.0%)
Hardy-Weinberg disequilibrium in the control group	20 (20.2%)	4 (11.4%)	4 (16.0%)
Association assessment			
Assess association between genotypes and BC with appropriate statistics and adjustment for confounders	25 (25.3%)	10 (28.6%)	5 (20.0%)
Assess association between genotypes and BC with appropriate statistics without adjustment for confounders	71 (71.7%)	23 (65.7%)	18 (72.0%)
Inappropriate statistics used	3 (3.0%)	2 (5.7%)	2 (8.0%)
Total sample size			
>1000	24 (24.2%)	3 (8.6%)	5 (20.0%)
500–1000	20 (20.2%)	9 (25.7%)	7 (28.0%)
200–500	38 (38.4%)	17 (48.6%)	7 (28.0%)
<200	17 (17.2%)	6 (17.1%)	6 (24.0%)

HWE: Hardy-Weinberg equilibrium, BC: breast cancer.

### Meta-Analysis Results

#### *TP53* Codon 72 Polymorphism

**FIGURE 2:**
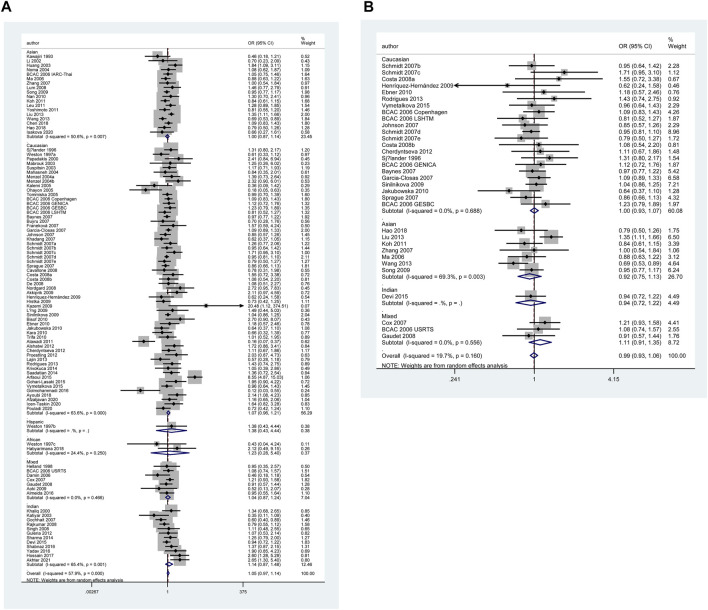
Forest plot of the association between *TP53* codon 72 polymorphism and BC risk in ethnicity subgroup analysis (GG vs. CC + CG) [**(A)**: Overall analysis; **(B)**: Sensitivity analysis].

The results of pooled analyses and ethnicity distribution are shown in Table 2 and Figure 2. It was indicated that *TP53* codon 72 polymorphism and BC risk did not have a strong link in overall analysis. In subgroup analyses, the quantitative syntheses of HWE-compliant studies (GG + CG vs. CC: OR = 1.06, 95% CI = 1.00–1.12; G vs. C: OR = 1.05, 95% CI = 1.00–1.10) and HWE in violation studies (G vs. C: OR = 0.84, 95% CI = 0.73–0.96) showed opposite conclusions. In both subgroup analyses of matched studies and subgroup analyses of controls with blood donors or volunteers (BV) source, the results showed increased risk of BC. In the analysis of clinical presentations, the expression of *TP53* codon 72 polymorphism was increased in subgroups of tumor grade I, premenopausal status, distant metastases negative, distant metastases positive, while the expression of *TP53* codon 72 polymorphism was decreased in the subgroups of PR-negative, tumor stage II, patients younger than 50 years and right localization.

**TABLE 2:** Pooled results on the association between the *TP53* codon 72 (rs1042522) polymorphism and BC risk.

Variable	n (Cases/Controls)	CG vs. CC			GG vs. CC			GG + CG vs. CC			GG vs. CC + CG			G vs. C		
		OR (95%CI)	*P*_h_/*I* ^2^ (%)	BFDP	OR (95%CI)	*P*_h_/*I* ^2^ (%)	BFDP	OR (95%CI)	*P*_h_/*I* ^2^ (%)	BFDP	OR (95%CI)	*P*_h_/*I* ^2^ (%)	BFDP	OR (95%CI)	*P*_h_/*I* ^2^ (%)	BFDP
Overall	99 (43,951/48,479)	0.99 (0.93, 1.06)^a^	<0.001/69.2	–	1.05 (0.96, 1.15)^a^	<0.001/65.2	–	1.00 (0.94, 1.07)^a^	<0.001/71.7	–	1.05 (0.97, 1.14)^a^	<0.001/57.9	–	1.02 (0.97, 1.06)^a^	<0.001/72.2	–
Matching																
No/NR	56 (26,429/25,940)	0.96 (0.88, 1.04)^a^	<0.001/69.7	–	0.95 (0.85, 1.07)^a^	<0.001/53.8	–	0.95 (0.88, 1.03)^a^	<0.001/69.6	–	0.98 (0.89, 1.08)^a^	<0.001/47.4	–	0.97 (0.92, 1.02)^a^	<0.001/64.5	–
Yes (include Age)	43 (17,522/22,539)	1.04 (0.95, 1.15)^a^	<0.001/68.8	–	**1.21 (1.03, 1.41)** **^a^**	**<0.001/72.8**	0.997	1.08 (0.98, 1.19)^a^	<0.001/73.8	–	**1.16 (1.02, 1.31)** **^a^**	**<0.001/66.0**	0.998	–	<0.001/77.8	–
Blinding and/or Quality control																
No	62 (14,222/15,943)	0.92 (0.82, 1.03)^a^	<0.001/73.3	–	0.99 (0.84, 1.16)^a^	<0.001/69.8	–	–	<0.001/75.3	–	1.05 (0.91, 1.20)^a^	<0.001/66.1	–	–	<0.001/76.6	–
Yes	37 (29,729/32,536)	1.03 (0.97, 1.10)^a^	<0.001/59.8	–	1.06 (0.96, 1.18)^a^	<0.001/54.6	–	1.04 (0.97, 1.11)^a^	<0.001/63.5	–	1.02 (0.94, 1.10)^a^	0.040/30.9	–	1.03 (0.98, 1.08)^a^	<0.001/60.6	–
HWE																
In violation	20 (2,951/4,112)	–	<0.001/83.6	–	0.77 (0.57, 1.03)^a^	<0.001/64.0	–	–	<0.001/79.6	–	0.95 (0.72, 1.26)^a^	<0.001/69.6	–	**0.84 (0.73, 0.96)** **^a^**	**<0.001/70.1**	0.997
Compliant	79 (41,000/44,367)	1.05 (0.99, 1.11)^a^	<0.001/57.4	–	1.10 (0.99, 1.22)^a^	<0.001/65.4	–	**1.06 (1.00, 1.12)** **^a^**	**<0.001/66.3**	1.000	1.06 (0.98, 1.15)^a^	<0.001/54.1	–	**1.05 (1.00, 1.10)** **^a^**	**<0.001/71.6**	1.000
Source of control																
PB	36 (26,360/31,229)	1.04 (0.99, 1.10)^a^	0.037/31.7	–	0.97 (0.90, 1.06)^a^	0.096/24.4	–	1.03 (0.98, 1.08)^a^	0.045/30.4	–	0.95 (0.90, 1.01)	0.143/20.3	–	1.00 (0.97, 1.03)	0.128/21.6	–
BV	19 (5,882/4,298)	1.00 (0.88, 1.14)^a^	0.024/43.3	–	**1.26 (1.03, 1.54)** **^a^**	**0.050/37.6**	0.998	1.04 (0.92, 1.19)^a^	0.007/49.8	–	**1.23 (1.06, 1.42)**	**0.195/21.4**	0.993	1.08 (0.97, 1.19)^a^	0.003/53.2	–
HB	19 (8,537/9,460)	–	<0.001/76.7	–	1.08 (0.85, 1.36)^a^	<0.001/73.9	–	–	<0.001/81.4	–	1.09 (0.92, 1.30)^a^	0.001/57.9	–	–	<0.001/81.4	–
NR	25 (3,172/3,492)	–	<0.001/84.1	–	–	<0.001/81.2	–	–	<0.001/84.8	–	–	<0.001/77.9	–	–	<0.001/85.7	–
Ethnicity																
African	2 (56/69)	0.86 (0.28, 2.64)	0.240/27.6	–	0.45 (0.07, 3.13)	0.886/0.0	–	0.80 (0.27, 2.40)	0.299/7.5	–	1.27 (0.41, 3.99)	0.250/24.4	–	1.00 (0.60, 1.67)	0.793/0.0	–
Asian	18 (7,180/7,539)	1.08 (0.95, 1.22)^a^	0.001/57.1	–	1.02 (0.84, 1.23)^a^	<0.001/68.0	–	1.06 (0.93, 1.22)^a^	<0.001/65.6	–	1.00 (0.87, 1.14)^a^	0.007/50.6	–	1.03 (0.94, 1.13)^a^	<0.001/67.7	–
Caucasian	60 (31,341/33,177)	0.98 (0.92, 1.06)^a^	<0.001/66.1	–	1.06 (0.93, 1.20)^a^	<0001/66.4	–	1.00 (0.93, 1.07)^a^	<0.001/69.9	–	1.08 (0.96, 1.21)^a^	<0.001/63.0	–	1.02 (0.96, 1.08)^a^	<0.001/74.1	–
Indian	12 (2,107/3,308)	–	<0.001/85.0	–	1.22 (0.85, 1.73)^a^	<0.001/73.0	–	–	<0.001/83.6	–	1.14 (0.87, 1.48)^a^	0.001/65.4	–	–	<0.001/77.7	–
Mixed	7 (3,267/4,386)	0.88 (0.70, 1.10)^a^	0.001/74.0	–	1.03 (0.86, 1.23)	0.112/41.8	–	–	<0.001/76.6	–	1.03 (0.87, 1.23)	0.466/0.0	–	0.91 (0.77, 1.07)^a^	0.002/71.5	–
Geographic region																
Africa	5 (479/505)	–	0.001/78.1	–	–	<0.001/89.9	–	–	<0.001/89.3	–	–	<0.001/84.7	–	–	<0.001/93.2	–
Asia	46 (11,532/13,047)	1.00 (0.88, 1.14)^a^	<0.001/74.4	–	0.99 (0.84, 1.16)^a^	<0.001/71.2	–	–	<0.001/75.4	–	0.99 (0.87, 1.12)^a^	<0.001/63.7	–	1.00 (0.92, 1.08)^a^	<0.001/73.3	–
Europe	37 (26,921/28,502)	1.00 (0.93, 1.07)^a^	<0.001/62.0	–	1.03 (0.96, 1.10)	0.246/13.1	–	1.01 (0.95, 1.08)^a^	<0.001/58.2	–	1.04 (0.97, 1.11)	0.406/3.7	–	1.02 (0.98, 1.07)^a^	0.002/44.5	–
North America	8 (4,641/5,928)	1.03 (0.95, 1.12)	0.243/23.4	–	1.01 (0.87, 1.19)	0.289/17.9	–	1.03 (0.95, 1.11)	0.203/28.2	–	0.98 (0.85, 1.14)	0.447/0.0	–	1.02 (0.96, 1.08)	0.227/25.3	–
South America	3 (378/497)	0.60 (0.34, 1.05)^a^	0.027/72.3	–	0.52 (0.23, 1.20)^a^	0.095/57.6	–	–	0.013/76.8	–	0.74 (0.48, 1.15)	0.360/2.1	–	0.68 (0.45, 1.03)^a^	0.025/73.0	–
ER status																
Negative	14 (911/2981)	0.85 (0.65, 1.12)^a^	0.006/56.5	–	0.97 (0.75, 1.25)	0.111/33.9	–	0.89 (0.70, 1.12)^a^	0.019/50.5	–	1.20 (0.85, 1.71)^a^	0.026/47.3	–	0.96 (0.83, 1.12)^a^	0.088/36.9	–
Positive	14 (2378/2981)	0.95 (0.74, 1.24)^a^	<0.001/72.1	–	1.05 (0.76, 1.47)^a^	0.008/55.6	–	0.99 (0.78, 1.26)^a^	<0.001/70.4	–	1.13 (0.81, 1.57)^a^	0.001/61.5	–	1.03 (0.88, 1.20)^a^	0.001/64.4	–
PR status																
Negative	9 (700/1936)	**0.67 (0.45, 0.99)** **^a^**	**0.002/69.5**	0.998	1.00 (0.62, 1.63)^a^	0.075/45.7	–	0.76 (0.54, 1.06)^a^	0.009/62.9	–	1.40 (0.84, 2.32)^a^	0.014/58.5	–	0.92 (0.74, 1.13)^a^	0.052/49.8	–
Positive	9 (1138/1936)	–	<0.001/82.2	–	0.90 (0.57, 1.42)^a^	0.049/50.4	–	–	<0.001/81.6	–	1.08 (0.73, 1.60)^a^	0.063/46.0	–	0.92 (0.71, 1.18)^a^	0.001/72.5	–
HER-2 status																
Negative	7 (608/1137)	–	<0.001/82.1	–	1.08 (0.51, 2.29)^a^	0.003/70.2	–	–	<0.001/82.7	–	1.11 (0.56, 2.17)^a^	0.005/67.6	–	–	<0.001/81.1	–
Positive	7 (322/1137)	1.01 (0.76, 1.34)	0.357/9.4	–	1.37 (0.74, 2.55)^a^	0.057/50.9	–	1.10 (0.84, 1.44)	0.448/0.0	–	1.36 (0.71, 2.61)^a^	0.016/61.5	–	1.12 (0.93, 1.35)	0.179/32.7	–
Tumor stage																
Stage 0 or I	9 (281/1382)	–	<0.001/84.0	–	0.81 (0.50, 1.33)	0.374/7.4	–	–	<0.001/79.1	–	0.86 (0.41, 1.79)^a^	0.076/43.8	–	0.93 (0.69, 1.26)^a^	0.056/47.2	–
Stage II	9 (482/1382)	–	<0.001/87.2	–	**0.59 (0.40, 0.87)**	**0.116/37.9**	0.992	–	<0.001/84.6	–	0.65 (0.37, 1.15)^a^	0.057/47.0		0.88 (0.66, 1.17)^a^	0.001/69.5	–
Stage III or Ⅳ	14 (442/2104)	0.94 (0.74, 1.18)	0.161/27.5	–	0.82 (0.58, 1.16)	0.200/23.4	–	0.90 (0.72, 1.12)	0.119/32.1	–	0.81 (0.59, 1.12)	0.494/0.0	–	0.90 (0.77, 1.05)	0.115/32.5	–
Tumor grade																
Grade I	11 (436/2642)	**1.30 (1.04, 1.64)**	**0.106/37.9**	0.998	**1.74 (1.22, 2.49)**	**0.592/0.0**	0.981	**1.38 (1.11, 1.71)**	**0.212/25.1**	0.988	**1.68 (1.22, 2.30)**	**0.295/15.6**	0.966	**1.34 (1.14, 1.57)**	**0.468/0.0**	0.919
Grade II	11 (995/2642)	–	<0.001/86.4	–	1.21 (0.78, 1.88)^a^	0.021/53.8	–	–	<0.001/83.2	–	1.40 (0.94, 2.07)^a^	0.022/52.2	–	1.08 (0.85, 1.37)^a^	<0.001/71.7	–
Grade III	12 (645/2860)	0.91 (0.75, 1.10)	0.215/24.0	–	1.06 (0.78, 1.44)	0.278/17.4	–	0.94 (0.79, 1.13)	0.391/5.5	–	1.22 (0.92, 1.62)	0.197/25.1	–	1.00 (0.87, 1.15)	0.381/6.6	–
Tumor size																
T1	5 (276/839)	0.92 (0.68, 1.24)	0.130/43.8	–	1.07 (0.68, 1.69)	0.280/21.2	–	0.97 (0.73, 1.28)	0.102/48.2	–	1.20 (0.78, 1.85)	0.571/0.0	–	1.03 (0.83, 1.27)	0.165/38.5	–
T2	5 (345/839)	1.09 (0.63, 1.89)^a^	0.006/72.3	–	1.29 (0.89, 1.89)	0.110/46.9	–	1.21 (0.76, 1.95)^a^	0.012/69.0	–	1.32 (0.93, 1.87)	0.160/39.2	–	1.25 (0.91, 1.71)^a^	0.027/63.7	–
T3 or T4	5 (101/800)	1.00 (0.63, 1.59)	0.998/0.0	–	1.14 (0.61, 2.12)	0.187/35.1	–	1.04 (0.67, 1.60)	0.807/0.0	–	1.13 (0.64, 1.99)	0.168/38.0	–	1.05 (0.77, 1.43)	0.223/29.8	–
Menopausal status																
Post-menopausal	13 (1748/2418)	–	<0.001/78.2	–	1.28 (0.88, 1.85)^a^	0.004/60.0	–	–	<0.001/76.2	–	1.42 (0.99, 2.04)^a^	<0.001/67.1	–	1.10 (0.90, 1.34)^a^	<0.001/71.2	–
Premenopausal	12 (1187/1952)	0.93 (0.70, 1.23)^a^	0.011/56.3	–	1.37 (0.91, 2.08)^a^	0.019/53.1	–	1.01 (0.77, 1.33)^a^	0.007/58.8	–	**1.43 (1.01, 2.03)** **^a^**	**0.044/45.2**	0.998	1.09 (0.89, 1.34)^a^	0.005/60.6	–
Age																
<40 years	4 (104/644)	1.19 (0.74, 1.91)	0.695/0.0	–	0.87 (0.26, 2.99)^a^	0.102/51.7	–	1.14 (0.72, 1.79)	0.991/0.0	–	0.78 (0.18, 3.40)^a^	0.020/69.5	–	0.97 (0.71, 1.32)	0.627/0.0	–
≥40 years	4 (548/644)	1.07 (0.83, 1.37)	0.774/0.0	–	–	<0.001/82.6	–	1.14 (0.90, 1.44)	0.657/0.0	–	–	<0.001/86.1	–	1.15 (0.84, 1.56)^a^	0.019/69.8	–
<45 years	3 (242/359)	–	<0.001/92.7	–	1.38 (0.56, 3.41)^a^	0.047/67.2	–	–	<0.001/91.9	–	0.85 (0.54, 1.35)	0.443/0.0	–	–	0.001/86.2	–
≥45 years	3 (218/359)	–	<0.001/90.7	–	–	0.009/78.7	–	–	<0.001/88.9	–	–	0.014/76.7	–	–	0.003/83.3	–
<50 years	6 (691/1070)	–	0.001/77.5	–	**0.56 (0.36, 0.86)**	**0.137/42.7**	0.992	–	<0.001/80.7	–	0.68 (0.45, 1.02)	0.493/0.0	–	–	0.003/75.5	–
≥50 years	6 (1328/1438)	–	<0.001/84.1	–	0.91 (0.66, 1.25)	0.193/34.3	–	–	0.001/79.5	–	1.36 (0.70, 2.63)^a^	0.006/69.6	–	0.80 (0.61, 1.05)^a^	0.049/58.1	–
Localization																
Left	2 (150/275)	0.72 (0.47, 1.13)	0.177/45.2	–	0.84 (0.48, 1.48)	0.374/0.0	–	0.76 (0.51, 1.15)	0.568/0.0	–	1.09 (0.47, 2.50)^a^	0.114/60.0	–	0.88 (0.66, 1.17)	0.603/0.0	–
Right	2 (145/275)	**0.61 (0.39, 0.95)**	**0.620/0.0**	0.997	0.74 (0.42, 1.30)	0.259/21.5	–	**0.64 (0.43, 0.97)**	**0.875/0.0**	0.998	1.02 (0.47, 2.25)^a^	0.142/53.5	–	0.79 (0.59, 1.06)	0.325/0.0	–
Histological subtype																
Ductal	7 (1511/1847)	0.98 (0.84, 1.13)	0.118/41.0	–	0.95 (0.64, 1.42)^a^	0.033/56.2	–	0.95 (0.75, 1.20)^a^	0.022/59.6	–	0.96 (0.76, 1.22)	0.131/39.1	–	0.98 (0.80, 1.19)^a^	0.007/66.1	–
Lobular	7 (235/1847)	0.81 (0.47, 1.39)^a^	0.056/51.0	–	0.85 (0.28, 2.52)^a^	0.035/55.8	–	0.66 (0.34, 1.30)^a^	0.003/69.7	–	1.36 (0.86, 2.13)	0.202/29.6	–	–	<0.001/75.6	–
Lymph node																
Negative	6 (534/1045)	–	<0.001/77.7	–	1.32 (0.70, 2.50)^a^	0.009/67.5	–	–	<0.001/82.2	–	1.26 (0.91, 1.73)	0.182/34.0	–	–	<0.001/81.1	–
Positive	8 (370/1403)	1.05 (0.75, 1.49)^a^	0.074/45.8	–	1.04 (0.62, 1.75)^a^	0.085/44.1	–	1.05 (0.75, 1.48)^a^	0.047/50.9	–	0.99 (0.70, 1.40)	0.166/32.8	–	1.03 (0.80, 1.33)^a^	0.033/54.0	–
Distant metastases																
Negative	2 (205/226)	1.59 (0.75, 3.39)^a^	0.075/68.5	–	**2.34 (1.32, 4.17)**	**0.805/0.0**	0.988	**1.74 (1.18, 2.56)**	**0.161/49.1**	0.989	**1.87 (1.10, 3.20)**	**0.574/0.0**	0.996	**1.59 (1.20, 2.11)**	**0.642/0.0**	0.969
Positive	2 (15/226)	0.65 (0.16, 2.73)	0.213/35.5	–	4.80 (0.51, 44.83)^a^	0.117/59.3	–	1.59 (0.20, 12.78)^a^	0.107/61.6	–	**5.09 (1.68, 15.38)**	**0.216/34.7**	0.994	2.32 (0.48, 11.33)^a^	0.050/74.0	–
Sensitivity analysis																
Overall	31 (30,675/34,821)	1.02 (0.97, 1.08)^a^	<0.001/51.8	–	1.01 (0.92, 1.10)^a^	0.002/47.8	–	1.02 (0.97, 1.08)^a^	<0.001/58.4	–	1.00 (0.94, 1.05)	0.139/21.9	–	1.01 (0.97, 1.05)^a^	<0.001/57.4	–
Ethnicity																
Asian	7 (3,923/4,732)	–	<0.001/75.1	–	–	<0.001/83.4	–	–	<0.001/82.5	–	0.92 (0.75, 1.13)^a^	0.003/69.3	–	–	<0.001/84.2	–
Caucasian	21 (23,972/26,425)	1.00 (0.95, 1.06)^a^	0.085/31.4	–	1.00 (0.93, 1.07)	0.525/0.0	–	1.00 (0.95, 1.06)^a^	0.065/34.1	–	1.00 (0.93, 1.07)	0.709/0.0	–	1.00 (0.97, 1.03)	0.107/28.9	–
Mixed	3 (2,780/3,664)	1.06 (0.88, 1.29)^a^	0.060/64.4	–	1.14 (0.93, 1.39)	0.654/0.0	–	1.06 (0.90, 1.25)^a^	0.103/56.1	–	1.11 (0.91, 1.35)	0.556/0.0	–	1.06 (0.97, 1.14)	0.275/22.5	–
Geographic region																
Asia	7 (3,923/4,732)	–	<0.001/75.1	–	–	<0.001/83.4	–	–	<0.001/82.5	–	0.92 (0.75, 1.13)^a^	0.003/69.3	–	–	<0.001/84.2	–
Europe	20 (22,319/24,571)	1.00 (0.95, 1.06)^a^	0.066/34.4	–	1.01 (0.94, 1.08)	0.525/0.0	–	1.00 (0.95, 1.06)^a^	0.048/37.3	–	1.01 (0.94, 1.08)	0.728/0.0	–	1.00 (0.96, 1.05)^a^	0.084/32.1	–
North America	4 (4,433/5,518)	1.04 (0.96, 1.13)	0.129/47.0	–	1.04 (0.88, 1.22)	0.363/6.0	–	1.04 (0.96, 1.13)	0.175/39.4	–	1.02 (0.87, 1.19)	0.330/12.5	–	1.03 (0.96, 1.10)	0.283/21.3	–

a Random-effects model was used in the pooled data.

Note: The bold values indicate significant results. CC, wild-type; CG, heterozygotes; GG, homozygous mutant; HWE, the Hardy-Weinberg equilibrium; PB, population-based; BV, blood donors or volunteers; HB, hospital-based; NR, not reported.

However, none of the above significant results could be considered robust after the BFDP test. For significant heterogeneity observed in this study, a meta-regression analysis was conducted to explore the source of heterogeneity from 13 items (including geographic region, ethnicity, sample size, source of controls, source of cases, ascertainment of cancer, ascertainment of control, matching, source of genotyping material of case, whether genotyping was done blindly and/or quality control, HWE, association assessment with appropriate statistics, and adjustment for confounders and quality score). The source of heterogeneity was only found in the geographic region of *p53* codon 72 polymorphism (CG vs. CC: *p* = 0.017, GG vs. CC: *p* = 0.034, GG + CG vs. CC: *p* = 0.005, G vs. C: *p* = 0.004). In other subgroup analyses (blinding and/or quality control, different ethnicity, various geographic region, ER status, HER-2 status, any tumor size, histological subtypes, and lymph node metastasis) and final sensitivity analyses (Figure 2), we found no association between *p53* codon 72 polymorphism and BC risk. Sensitivity analysis on clinical presentations could not be performed due to the lack of high-quality studies. There was no significant publication bias confirmed by Egger’s test and Begg’s funnel plot (Figure 7).

#### IVS3 16 bp Polymorphism

**FIGURE 3:**
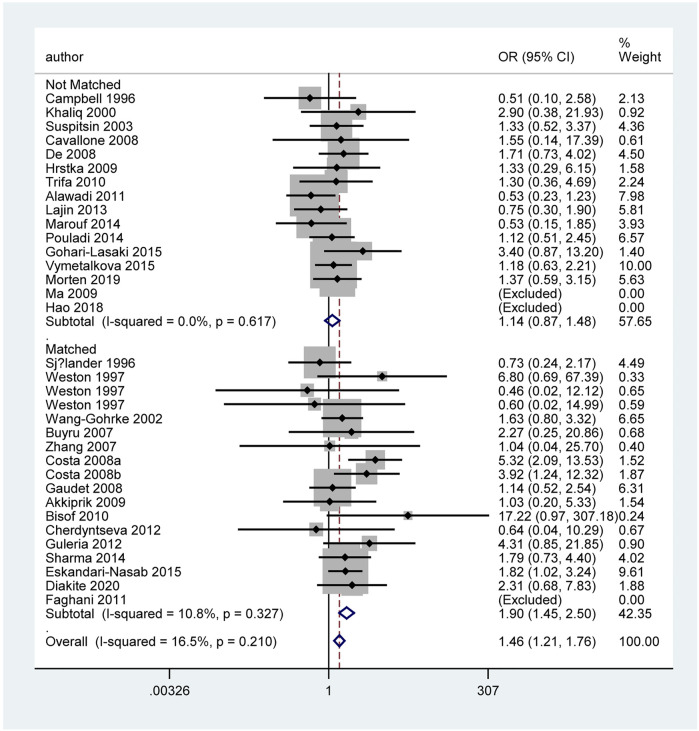
Forest plot of the association between *TP53* IVS3 16 bp polymorphism and BC risk in stratification analysis (GG vs. CC).

**FIGURE 4:**
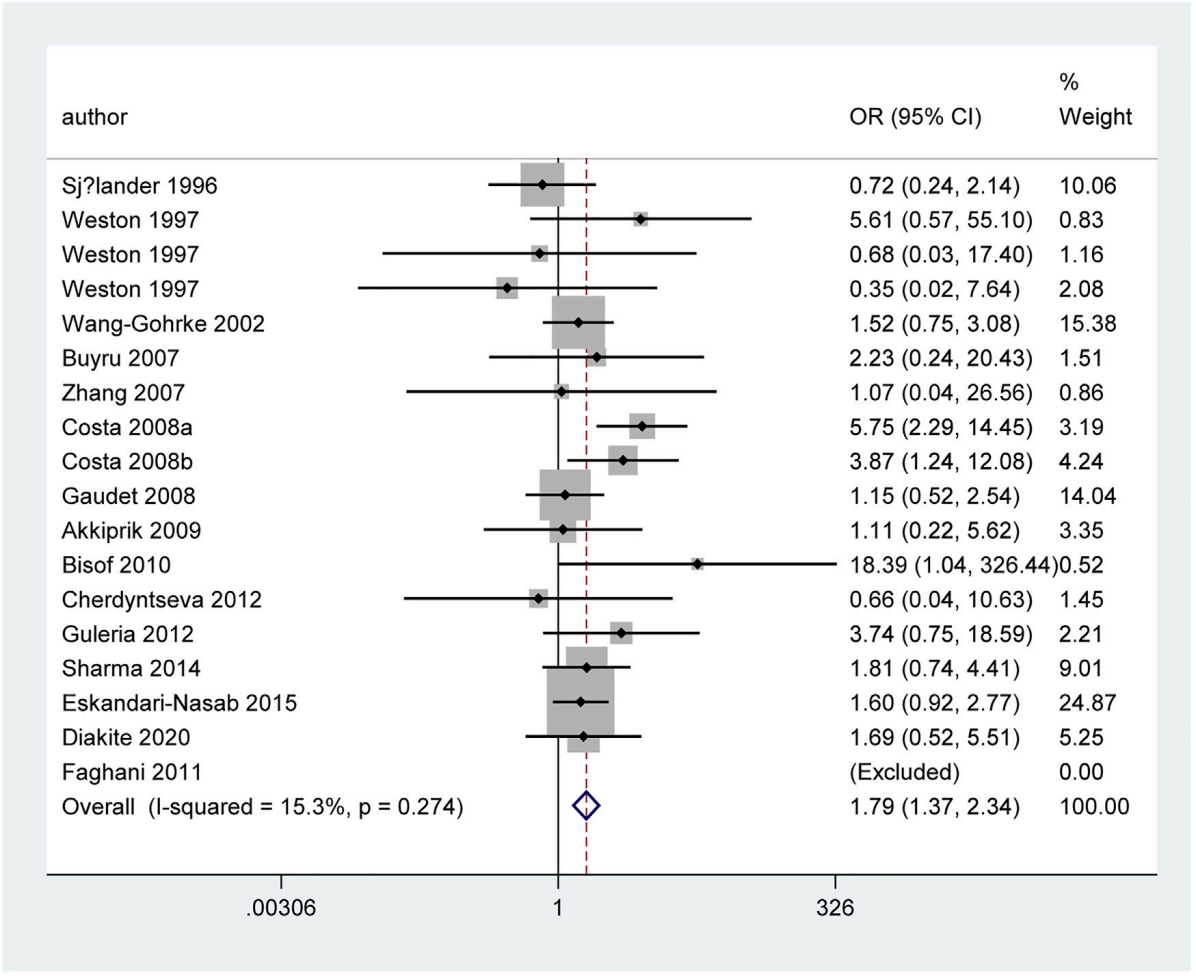
Forest plot of the association between *TP53* IVS3 16 bp polymorphism and BC risk in matched subgroup analysis (GG vs. CC + CG).

**FIGURE 5:**
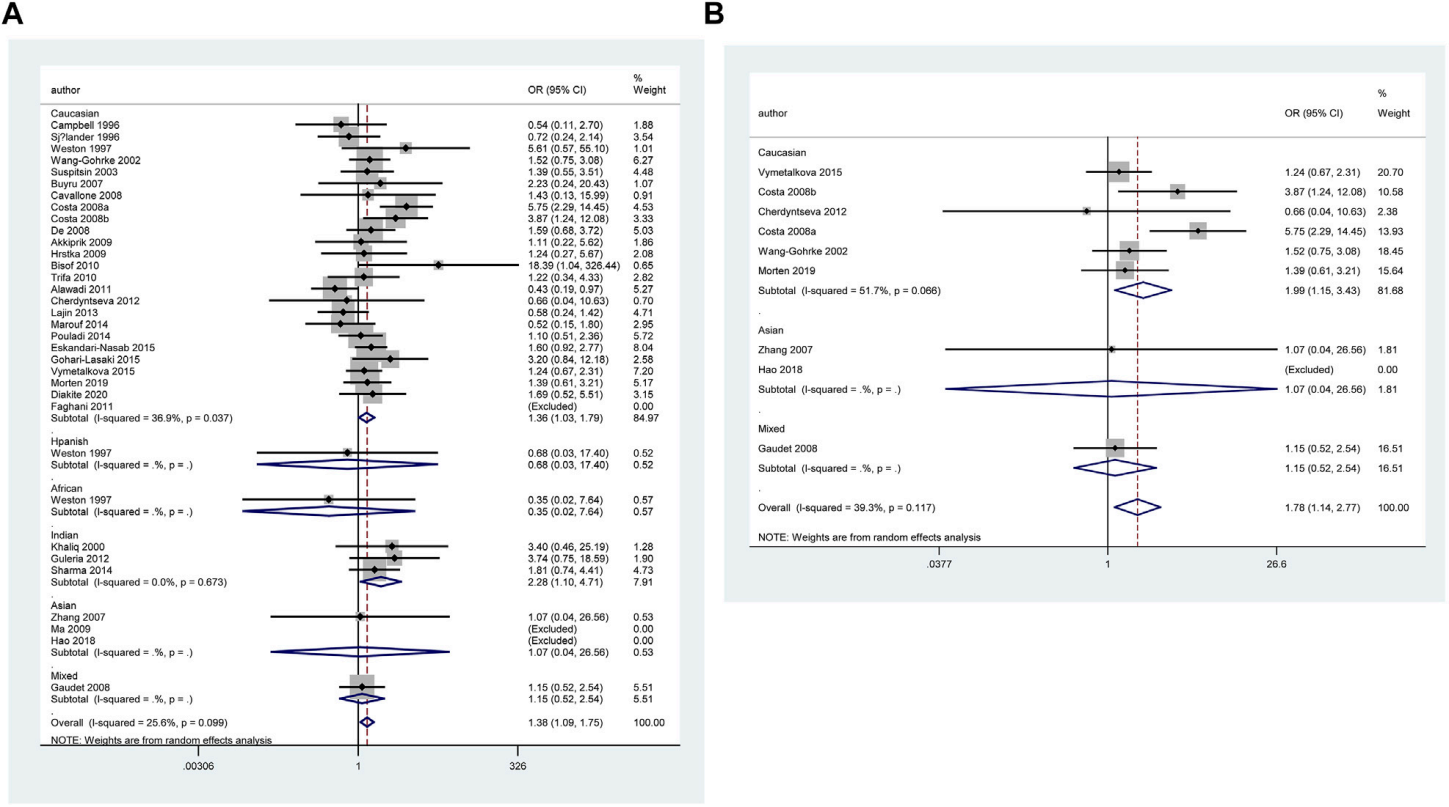
Forest plot of the association between *TP53* IVS3 16 bp polymorphism and BC risk in ethnicity subgroup analysis (GG vs. CC + CG) [**(A)**: Overall analysis; **(B)**: Sensitivity analysis].

Table 3 and Figure 5 show the association between *TP53* IVS3 16 bp polymorphism and BC risk and the distribution of this polymorphism in different ethnic groups. It is indicated that *TP53* IVS3 16 bp polymorphism increased BC risk in overall population (Figure 3). This polymorphism significantly increased BC risk as shown in the quantitative syntheses of the following subgroup analyses: matched studies (Figures 3, 4), neither blinding nor quality control, blinding and/or quality control, HWE-compliant studies, controls of population-based (PB) source, controls of BV source, controls of not reported (NR) source, Caucasians, Indian, women in Asia, women in Europe, tumor stage 0 or I, tumor stage II, tumor stage III or Ⅳ, tumor grade I, tumor size below 2 cm, and ductal carcinoma subtype.

**TABLE 3:** Pooled results on the association between the *TP53* IVS3 16bp (rs17878362) polymorphism and BC risk.

Variable		n (Cases/Controls)		CG vs. CC			GG vs. CC			GG + CG vs. CC			GG vs. CC + CG			G vs. C		
				OR (95%CI)	*P*_h_/*I* ^2^ (%)	BFDP	OR (95%CI)	*P*_h_/*I* ^2^ (%)	BFDP	OR (95%CI)	*P*_h_/*I* ^2^ (%)	BFDP	OR (95%CI)	*P*_h_/*I* ^2^ (%)	BFDP	OR (95%CI)	*P*_h_/*I* ^2^ (%)	BFDP
Overall		35 (8,705/7,516)		1.13 (0.99, 1.27)^a^	<0.001/52.0	–	**1.46 (1.21, 1.76)**	**0.210/16.5**	0.738	**1.16 (1.04, 1.29)** **^a^**	**0.001/48.9**	0.996	**1.38 (1.09, 1.75)** **^a^**	**0.099/25.6**	0.994	**1.15 (1.06, 1.26)** **^a^**	**0.015/37.8**	0.993
Matching																		
No/NR		17 (5,585/3,616)		1.07 (0.93, 1.23)^a^	0.097/33.1	–	1.14 (0.87, 1.48)	0.617/0.0	–	1.05 (0.95, 1.16)	0.301/13.1	–	1.09 (0.84, 1.41)	0.353/9.1	–	1.05 (0.96, 1.14)	0.475/0.0	–
Yes (include age)		18 (3,120/3,900)		1.17 (0.95, 1.43)^a^	<0.001/61.8	–	**1.90 (1.45, 2.50)**	**0.327/10.8**	0.173	**1.25 (1.03, 1.52)** **^a^**	**<0.001/61.4**	0.998	**1.79 (1.38, 2.34)**	**0.274/15.3**	0.447	**1.25 (1.09, 1.44)** **^a^**	**0.018/45.7**	0.986
Blinding and/or quality control																		
No		24 (4,813/4,283)		**1.23 (1.04, 1.45)** **^a^**	**<0.001/56.8**	0.997	**1.34 (1.06, 1.69)**	**0.355/8.0**	0.996	**1.25 (1.06, 1.46)** **^a^**	**0.001/55.3**	0.993	1.23 (0.98, 1.55)	0.245/16.5	–	**1.18 (1.06, 1.32)** **^a^**	**0.031/38.1**	0.994
Yes		11 (3,892/3,233)		0.93 (0.83, 1.05)	0.759/0.0	–	**1.71 (1.24, 2.35)**	**0.202/26.2**	0.959	1.00 (0.89, 1.11)	0.636/0.0	–	**1.73 (1.26, 2.38)**	**0.188/27.8**	0.950	1.05 (0.95, 1.17)	0.165/30.5	–
HWE																		
In violation		5 (1,009/1,438)		–	<0.001/83.8	–	1.03 (0.46, 2.31)^a^	0.113/54.1	–	–	<0.001/82.4	–	1.18 (0.76, 1.83)	0.164/44.8	–	1.26 (0.91, 1.73)^a^	0.018/70.3	–
Compliant		30 (7,696/6,078)		1.07 (0.96, 1.19)^a^	0.043/32.9	–	**1.50 (1.22, 1.85)**	**0.258/13.8**	0.839	**1.11 (1.00, 1.23)** **^a^**	**0.074/28.7**	0.999	**1.44 (1.17, 1.76)**	**0.109/25.6**	0.923	**1.13 (1.03, 1.23)** **^a^**	**0.080/27.9**	0.996
Source of control																		
PB		17 (4,978/3,820)		1.13 (0.96, 1.33)^a^	0.037/42.5	–	1.23 (0.93, 1.63)	0.435/1.4	–	**1.11 (1.00, 1.23)**	**0.109/31.0**	0.999	1.13 (0.86, 1.49)	0.261/17.2	–	**1.10 (1.00, 1.21)**	**0.184/23.9**	0.999
BV		8 (1,767/1,951)		1.01 (0.87, 1.18)	0.488/0.0	–	**1.76 (1.19, 2.62)**	**0.101/41.6**	0.990	1.07 (0.93, 1.25)	0.520/0.0	–	1.73 (0.98, 3.06)^a^	0.066/47.2	–	1.12 (0.99, 1.28)	0.249/22.7	–
HB		3 (924/879)		–	<0.001/93.5	–	1.18 (0.63, 2.21)	–	–	–	<0.001/93.3	–	1.24 (0.67, 2.31)	–	–	–	0.001/86.3	–
NR		7 (1,036/866)		1.08 (0.88, 1.32)	0.628/0.0	–	**1.85 (1.24, 2.76)**	**0.574/0.0**	0.981	1.16 (0.94, 1.43)	0.337/12.1	–	**1.73 (1.17, 2.55)**	**0.658/0.0**	0.990	**1.22 (1.04, 1.43)**	**0.202/29.7**	0.997
Ethnicity																		
African		1 (16/30)		2.60 (0.67, 10.07)	–	–	0.60 (0.02, 14.99)	–	–	2.29 (0.60, 8.78)	–	–	0.35 (0.02, 7.64)	–	–	1.30 (0.53, 3.18)	–	–
Asian		3 (454/644)		1.14 (0.45, 2.89)^a^	0.064/63.6	–	1.04 (0.04, 25.70)	–	–	1.12 (0.43, 2.90)^a^	0.057/65.2	–	1.07 (0.04, 26.56)	–	–	1.10 (0.43, 2.79)^a^	0.056/65.2	–
Caucasian		27 (6,687/6,131)		1.14 (0.99, 1.31)^a^	<0.001/56.8	–	**1.43 (1.17, 1.75)**	**0.108/26.9**	0.942	**1.17 (1.03, 1.32)** **^a^**	**0.001/53.7**	0.997	**1.35 (1.03, 1.78)** **^a^**	**0.048/34.5**	0.998	**1.16 (1.05, 1.27)** **^a^**	**0.012/42.8**	0.986
Indian		3 (970/321)		0.98 (0.71, 1.35)	0.250/27.9	–	**2.35 (1.14, 4.87)**	**0.627/0.0**	0.997	1.11 (0.82, 1.51)	0.248/28.4	–	**2.38 (1.16, 4.89)**	**0.673/0.0**	0.996	1.22 (0.95, 1.58)	0.321/12.1	–
Mixed		1 (578/390)		0.98 (0.73, 1.31)	–	–	1.15 (0.52, 2.54)	–	–	1.00 (0.75, 1.31)	–	–	1.15 (0.52, 2.54)	–	–	1.01 (0.79, 1.29)	–	–
Geographic region																		
Africa		3 (274/306)		1.32 (0.92, 1.89)	0.317/13.0	–	1.16 (0.58, 2.33)	0.252/27.4	–	1.28 (0.91, 1.80)	0.182/41.3	–	1.03 (0.52, 2.04)	0.386/0.0	–	1.21 (0.80, 1.83)^a^	0.132/50.7	–
Asia		14 (2,689/2,025)		1.28 (0.99, 1.65)^a^	0.001/62.5	–	**1.39 (1.03, 1.88)**	**0.251/20.2**	0.998	**1.32 (1.04, 1.66)** **^a^**	**0.003/59.0**	0.997	1.26 (0.94, 1.69)	0.104/36.9	–	**1.24 (1.11, 1.39)**	**0.126/31.2**	0.919
Europe		13 (3,761/4,174)		1.01 (0.90, 1.12)	0.306/14.2	–	**1.65 (1.23, 2.21)**	**0.116/34.3**	0.952	1.05 (0.95, 1.17)	0.414/3.3	–	**1.66 (1.10, 2.51)** **^a^**	**0.092/37.4**	0.996	1.10 (0.99, 1.20)	0.156/29.5	–
North America		4 (677/575)		1.16 (0.58, 2.36)^a^	0.025/67.9	–	1.28 (0.64, 2.56)	0.440/0.0	–	1.17 (0.56, 2.42)^a^	0.015/71.4	–	1.23 (0.62, 2.44)	0.475/0.0	–	1.12 (0.62, 2.02)^a^	0.020/69.5	–
Oceania		1 (1,304/436)		0.92 (0.71, 1.18)	–	–	1.37 (0.59, 3.15)	–	–	0.94 (0.74, 1.21)	–	–	1.39 (0.61, 3.21)	–	–	0.98 (0.78, 1.23)	–	–
ER status																		
Negative			5 (902/1056)	1.13 (0.76, 1.68)^a^	0.089/53.9	–	1.94 (0.80, 4.73)^a^	0.085/54.7	–	1.28 (0.80, 2.07)^a^	0.007/71.7	–	**2.19 (1.39, 3.46)**	**0.204/34.7**	0.955	–	<0.001/84.8	–
Positive			5 (1079/1056)	1.00 (0.81, 1.23)	0.230/30.4	–	–	0.002/79.6	–	–	0.001/78.6	–	–	0.006/75.8	–	–	<0.001/91.7	–
PR status																		
Negative			4 (849/860)	1.11 (0.88, 1.41)	0.238/30.4	–	2.00 (0.68, 5.90)^a^	0.023/73.4	–	1.45 (0.91, 2.30)^a^	0.028/66.9	–	1.85 (0.67, 5.13)^a^	0.030/71.5	–	–	<0.001/88.7	–
Positive			3 (275/424)	–	0.035/77.4	–	–	0.005/87.6	–	–	0.001/85.9	–	–	0.015/83.2	–	–	<0.001/93.7	–
HER-2 status																		
Negative			2 (796/639)	1.17 (0.75, 1.81)^a^	0.124/57.8	–	–	0.025/80.1	–	–	0.001/91.1	–	2.75 (0.99, 7.58)^a^	0.052/73.4	–	–	<0.001/95.3	–
Positive			1 (160/203)	**1.81 (1.12, 2.93)**	**–**	0.995	**4.64 (2.56, 8.38)**	**–**	0.084	**2.54 (1.65, 3.90)**	**–**	0.510	**3.56 (2.06, 6.15)**	**–**	0.357	**2.50 (1.84, 3.40)**	**–**	0.001
Tumor stage																		
Stage 0 or I			3 (159/453)	**1.81 (1.19, 2.77)**	**0.384/0.0**	0.991	**–**	0.018/75.3	–	**2.39 (1.05, 5.44)** **^a^**	**0.035/70.2**	0.998	**3.12 (1.00, 9.72)** **^a^**	**0.048/67.0**	0.998	–	0.003/83.1	–
Stage II			2 (143/283)	1.40 (0.90, 2.20)	0.866/0.0	–	**3.03 (1.62, 5.69)**	**0.920/0.0**	0.958	**1.73 (1.15, 2.60)**	**0.632/0.0**	0.993	**2.62 (1.45, 4.73)**	**0.877/0.0**	0.976	**1.79 (1.31, 2.43)**	**0.547/0.0**	0.853
Stage III or Ⅳ			5 (249/736)	**1.40 (1.00, 1.97)**	**0.314/15.8**	0.998	**3.76 (1.63, 8.69)** **^a^**	**0.040/60.0**	0.988	**1.98 (1.19, 3.31)** **^a^**	**0.054/57.0**	0.993	**3.18 (1.60, 6.33)** **^a^**	**0.089/50.5**	0.975	**2.04 (1.24, 3.33)** **^a^**	**0.003/74.5**	0.988
Tumor grade																		
Grade I			5 (203/1549)	1.05 (0.72, 1.53)	0.309/16.4	–	**2.31 (1.25, 4.25)**	**0.538/0.0**	0.992	1.14 (0.83, 1.58)	0.135/43.0	–	**2.08 (1.16, 3.74)**	**0.680/0.0**	0.995	1.19 (0.68, 2.09)^a^	0.031/66.1	–
Grade II			5 (612/1549)	1.12 (0.75, 1.66)^a^	0.086/54.5	–	–	0.002/79.7	–	–	<0.001/82.7	–	1.68 (0.57, 4.92)^a^	0.010/73.7	–	–	<0.001/91.6	–
Grade III			5 (1034/1549)	0.97 (0.77, 1.23)	0.827/0.0	–	1.28 (0.45, 3.66)^a^	0.095/52.9	–	1.31 (0.82, 2.10)^a^	0.017/66.9	–	1.27 (0.46, 3.52)^a^	0.097/52.5	–	1.07 (0.71, 1.63)^a^	0.038/64.4	–
Tumor size																		
T1			2 (129/317)	**1.76 (1.07, 2.88)**	**0.552/0.0**	0.997	**4.64 (2.57, 8.39)**	**0.171/46.6**	0.088	**2.45 (1.58, 3.80)**	**0.197/39.9**	0.730	**3.63 (2.10, 6.28)**	**0.229/30.9**	0.311	1.99 (0.88, 4.53)^a^	0.093/64.6	–
T2			2 (235/317)	1.07 (0.43, 2.68)^a^	0.055/72.8	–	–	0.008/85.7	–	–	0.002/89.8	–	–	0.021/81.3	–	–	<0.001/93.1	–
T3 or T4			2 (21/317)	1.10 (0.41, 2.97)	0.661/0.0	–	0.54 (0.07, 4.40)	0.453/0.0	–	0.85 (0.32, 2.27)	0.643/0.0	–	0.57 (0.07, 4.51)	0.315/0.9	–	0.71 (0.29, 1.73)	0.827/0.0	–
Menopausal status																		
Post-menopausal			3 (279/321)	0.86 (0.52, 1.42)	0.948/0.0	–	0.29 (0.04, 2.07)	0.639/0.0	–	0.84 (0.57, 1.23)	0.892/0.0	–	0.30 (0.04, 2.13)	0.638/0.0	–	0.75 (0.48, 1.17)	0.453/0.0	–
Pre-menopausal			3 (208/315)	1.03 (0.66, 1.62)	0.671/0.0	–	0.77 (0.22, 2.67)	–	–	0.97 (0.66, 1.44)	0.920/0.0	–	0.74 (0.21, 2.54)	–	–	0.97 (0.66, 1.42)	0.928/0.0	–
Histological subtype																		
Ductal			2 (288/317)	**1.48 (1.02, 2.13)**	**0.283/13.4**	0.998	–	0.004/87.7	–	–	0.018/82.1	–	–	0.009/85.3	–	–	0.001/90.6	–
Lobular			1 (4/114)	0.93 (0.09, 9.30)	–	–	1.32 (0.06, 27.76)	–	–	0.72 (0.07, 7.19)	–	–	1.39 (0.07, 28.08)	–	–	0.60 (0.07, 4.98)	–	–
Lymph node																		
Negative			3 (190/487)	1.21 (0.57, 2.57)^a^	0.052/66.1	–	–	0.002/83.9	–	–	0.001/86.4	–	–	0.008/79.4	–	–	<0.001/91.6	–
Positive			3 (374/487)	1.21 (0.89, 1.65)	0.229/32.1	–	–	0.007/80.1	–	–	0.009/78.7	–	1.35 (0.46, 4.01)^a^	0.019/74.9	–	–	<0.001/87.6	–
Sensitivity analysis																		
Overall		9 (4,001/3,359)		0.96 (0.86, 1.08)	0.494/0.0	–	**1.63 (1.18, 2.25)**	**0.145/35.5**	0.984	1.01 (0.90, 1.13)	0.421/1.6	–	**1.64 (1.19, 2.26)**	**0.117/39.3**	0.981	1.06 (0.96, 1.17)	0.167/31.4	–
Ethnicity																		
Asian		2 (337/521)		0.82 (0.48, 1.41)	0.314/1.5	–	1.04 (0.04, 25.7)	–	–	0.81 (0.48, 1.39)	0.275/16.0	–	1.07 (0.04, 26.56)	–	–	0.81 (0.48, 1.36)	0.244/26.3	–
Caucasian		6 (3,086/2,448)		0.97 (0.85, 1.10)	0.290/19.0	–	**1.95 (1.15, 3.31)** **^a^**	**0.084/48.5**	0.995	1.02 (0.90, 1.16)	0.263/22.7	–	**1.99 (1.15, 3.43)** **^a^**	**0.066/51.7**	0.995	1.10 (0.94, 1.28)^a^	0.096/46.4	–
Mixed		1 (578/390)		0.98 (0.73, 1.31)	–	–	1.15 (0.52, 2.54)	–	–	1.00 (0.75, 1.31)	–	–	1.15 (0.52, 2.54)	–	–	1.01 (0.79, 1.29)	–	–
Geographic region																		
Asia		2 (337/521)		0.82 (0.48, 1.41)	0.314/1.5	–	1.04 (0.04, 25.7)	–	–	0.81 (0.48, 1.39)	0.275/16.0	–	1.07 (0.04, 26.56)	–	–	0.81 (0.48, 1.36)	0.244/26.3	–
Europe		5 (1,782/2,012)		0.99 (0.85, 1.15)	0.204/32.6	–	**2.15 (1.11, 4.14)** **^a^**	**0.057/56.5**	0.996	1.05 (0.91, 1.21)	0.203/32.7	–	**2.18 (1.11, 4.29)** **^a^**	**0.043/59.4**	0.997	1.13 (0.93, 1.38)^a^	0.078/52.4	–
North America		1 (578/390)		0.98 (0.73, 1.31)	–	–	1.15 (0.52, 2.54)	–	–	1.00 (0.75, 1.31)	–	–	1.15 (0.52, 2.54)	–	–	1.01 (0.79, 1.29)	–	–
Oceania		1 (1,304/436)		0.92 (0.71, 1.18)	–	–	1.37 (0.59, 3.15)	–	–	0.94 (0.74, 1.21)	–	–	1.39 (0.61, 3.21)	–	–	0.98 (0.78, 1.23)	–	–
ER status																		
Negative	2 (722/632)			0.93 (0.71, 1.21)	0.408/0.0	–	1.47 (0.62, 3.47)	0.761/0.0	–	0.95 (0.74, 1.23)	0.329/0.0	–	1.49 (0.63, 3.51)	0.793/0.0	–	0.99 (0.79, 1.25)	0.282/13.5	–
Positive	2 (789/632)			0.88 (0.69, 1.14)	0.715/0.0	–	1.11 (0.46, 2.68)	0.566/0.0	–	0.90 (0.70, 1.15)	0.831/0.0	–	1.15 (0.48, 2.76)	0.556/0.0	–	0.92 (0.74, 1.15)	0.977/0.0	–

a Random-effects model was used in the pooled data.

Note: The bold values indicate significant results.

However, when we used BFDP to redress the above significant results, an association remained significant only in overall analysis (GG vs. CC: BFDP = 0.738), matched studies (GG vs. CC: BFDP = 0.173; GG vs. CC + CG: BFDP = 0.447), and tumor size below 2 cm (GG vs. CC: BFDP = 0.088; GG + CG vs. CC: BFDP = 0.730; GG vs. CC + CG: BFDP = 0.311). No source of heterogeneity was found in the 13 items mentioned above by meta-regression analysis. Then, in sensitivity analyses (Figure 5), an association with BC risk was found again in overall analysis (GG vs. CC: OR = 1.63, 95% CI = 1.18–2.25; GG vs. CC + CG: OR = 1.64, 95% CI = 1.19–2.26), Caucasians subgroup (GG vs. CC: OR = 1.95, 95% CI = 1.15–3.31; GG vs. CC + CG: OR = 1.99, 95% CI = 1.15–3.43), and women in Europe subgroup (GG vs. CC: OR = 2.15, 95% CI = 1.11–4.14; GG vs. CC + CG: OR = 2.18, 95% CI = 1.11–4.29), but the BFDP of these results were higher than 0.8. Egger’s test and Begg’s funnel plot confirmed that there was no publication bias in these polymorphism analyses (Figure 7).

#### IVS6+62A > G Polymorphism

**FIGURE 6:**
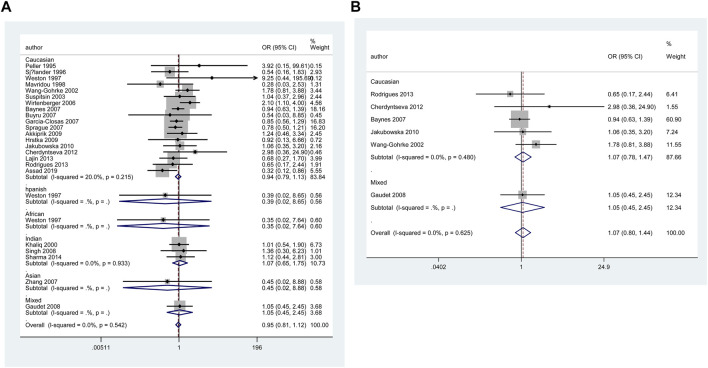
Forest plot of the association between *TP53* IVS6+62A > G polymorphism and BC risk in ethnicity subgroup analysis (GG vs. CC + CG) [**(A)**: Overall analysis; **(B)**: Sensitivity analysis].

**FIGURE 7:**
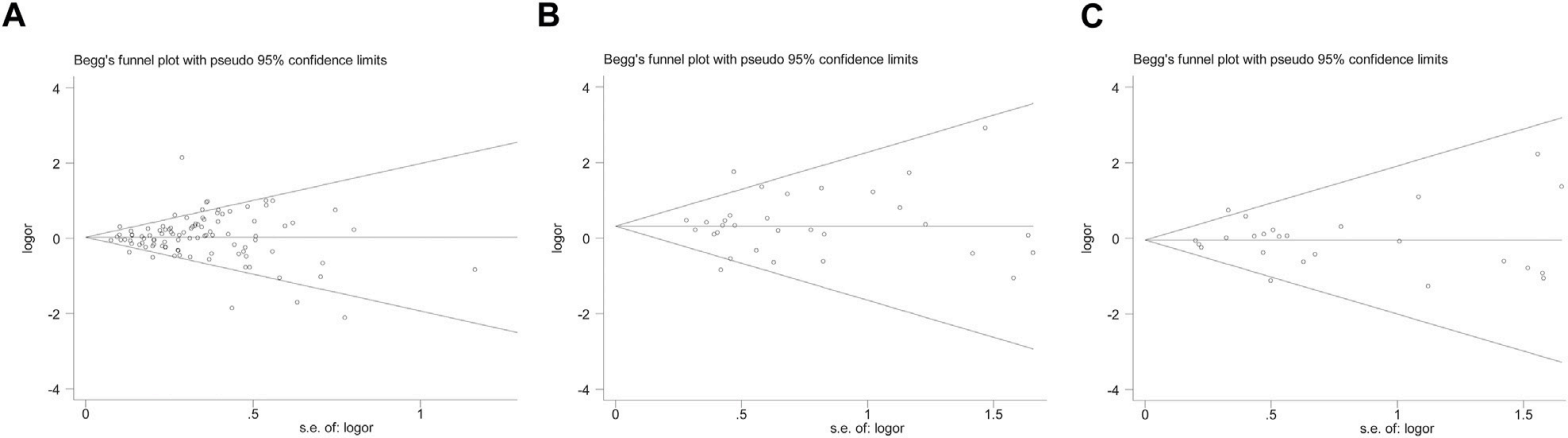
Begg’s funnel plot to assess publication bias on the combined effects of *TP53* polymorphisms with BC risk in overall population (GG vs. CC + CG) [**(A)**: codon 72 polymorphism; **(B)**: IVS3 16 bp polymorphism; **(C)**: IVS6+62A > G polymorphism].

Table 4 and Figure 6 show the association between *TP53* IVS6+62A > G polymorphism and BC risk and the distribution of this polymorphism in different ethnic groups. The quantitative syntheses showed that IVS6 + 62 polymorphism was not associated with BC risk in all analyses, except in controls of BV source subgroup (GG vs. CC: OR = 1.74, 95% CI = 1.05–2.91; GG vs. CC + CG: OR = 1.73, 95% CI = 1.04–2.88), which the correction results showed BFDP >0.8. In the analysis of relevant clinical features, some significant results emerged, such as ER negative, ER positive, and premenopausal status, but the quality of these pooled studies makes it hard to trust the statistical results of their data. The results of meta-regression analysis showed that the source of heterogeneity did not come from the 13 items (mentioned above), and Egger’s test and Begg’s funnel plot showed no publication bias here (Figure 7).

**TABLE 4:** Pooled results on the association between the *TP53* IVS6+62A > G (rs1625895) polymorphism and BC risk.

Variable		n (Cases/Controls)	CG vs. CC			GG vs. CC			GG + CG vs. CC			GG vs. CC + CG			G vs. C		
			OR (95%CI)	*P*_h_/*I* ^2^ (%)	BFDP	OR (95%CI)	*P*_h_/*I* ^2^ (%)	BFDP	OR (95%CI)	*P*_h_/*I* ^2^ (%)	BFDP	OR (95%CI)	*P*_h_/*I* ^2^ (%)	BFDP	OR (95%CI)	*P*_h_/*I* ^2^ (%)	BFDP
Overall		25 (12,222/12,895)	1.01 (0.89, 1.14)^a^	<0.001/61.6	–	0.93 (0.79, 1.11)	0.180/20.4	–	1.00 (0.89, 1.14)^a^	<0.001/65.0	–	0.95 (0.81, 1.12)	0.542/0.0	–	1.00 (0.90, 1.11)^a^	<0.001/62.7	–
Matching																	
No/NR		12 (5,551/5,024)	0.91 (0.72, 1.14)^a^	<0.001/73.7	–	0.84 (0.55, 1.29)^a^	0.045/45.0	–	–	<0.001/75.6	–	0.96 (0.76, 1.22)	0.267/18.0	–	0.92 (0.77, 1.10)^a^	<0.001/73.0	–
Yes (include Age)		13 (6,671/7,871)	1.06 (0.98, 1.15)	0.139/30.6	–	0.96 (0.76, 1.21)	0.604/0.0	–	1.08 (0.95, 1.23)^a^	0.057/41.7	–	0.94 (0.75, 1.19)	0.689/0.0	–	1.06 (0.94, 1.20)^a^	0.043/44.2	–
Blinding and/or quality control																	
No		14 (2,908/2,658)	0.87 (0.64, 1.18)^a^	<0.001/71.4	–	0.80 (0.49, 1.31)^a^	0.085/36.4	–	0.86 (0.63, 1.17)^a^	<0.001/74.1	–	0.90 (0.67, 1.22)	0.381/6.4	–	0.89 (0.70, 1.13)^a^	<0.001/71.8	–
Yes		11 (9,314/10,237)	1.03 (0.96, 1.11)	0.104/36.9	–	0.98 (0.80, 1.20)	0.514/0.0	–	1.05 (0.95, 1.16)^a^	0.078/40.7	–	0.97 (0.80, 1.19)	0.565/0.0	–	1.04 (0.95, 1.14)^a^	0.078/40.6	–
HWE																	
In violation		4 (4,624/6,185)	1.03 (0.94, 1.14)	0.128/47.3	–	0.79 (0.59, 1.05)	0.869/0.0	–	1.01 (0.92, 1.11)	0.115/49.4	–	0.79 (0.59, 1.05)	0.890/0.0	–	0.99 (0.91, 1.08)	0.131/46.7	–
Compliant		21 (7,598/6,710)	1.00 (0.85, 1.18)^a^	<0.001/64.6	–	1.02 (0.83, 1.26)	0.123/27.1	–	1.00 (0.85, 1.18)^a^	<0.001/68.0	–	1.05 (0.86, 1.29)	0.482/0.0	–	1.00 (0.87, 1.15)^a^	<0.001/65.9	–
Source of control																	
PB		13 (6,801/6,958)	1.06 (0.92, 1.21)^a^	0.044/44.2	–	0.90 (0.72, 1.13)	0.725/0.0	–	1.05 (0.91, 1.21)^a^	0.028/47.7	–	0.92 (0.74, 1.14)	0.783/0.0	–	1.02 (0.91, 1.15)^a^	0.051/42.7	–
BV		4 (1,882/1,692)	1.04 (0.73, 1.46)^a^	0.011/73.1	–	**1.74 (1.05, 2.91)**	**0.549/0.0**	0.997	1.07 (0.76, 1.51)^a^	0.009/74.1	–	**1.73 (1.04, 2.88)**	**0.598/0.0**	0.997	1.08 (0.80, 1.46)^a^	0.013/72.2	–
HB		3 (2,871/3,583)	–	<0.001/92.0	–	–	0.010/78.1	–	–	<0.001/93.2	–	0.75 (0.52, 1.08)	0.154/46.5	–	–	<0.001/92.8	–
NR		5 (668/662)	1.09 (0.83, 1.41)	0.424/0.0	–	1.08 (0.59, 1.99)	0.670/0.0	–	1.08 (0.84, 1.39)	0.454/0.0	–	1.03 (0.57, 1.88)	0.669/0.0	–	1.06 (0.85, 1.32)	0.531/0.0	–
Ethnicity																	
African		1 (16/30)	3.25 (0.75, 14.02)	–	–	0.71 (0.03, 18.60)	–	–	2.89 (0.68, 12.35)	–	–	0.35 (0.02, 7.64)	–	–	1.37 (0.56, 3.32)	–	–
Asian		1 (83/268)	1.02 (0.44, 2.37)	–	–	0.46 (0.02, 8.91)	–	–	0.91 (0.40, 2.09)	–	–	0.45 (0.02, 8.88)	–	–	0.83 (0.37, 1.83)	–	–
Caucasian		19 (10,552/11,861)	1.01 (0.88, 1.15)^a^	<0.001/68.0	–	0.94 (0.70, 1.25)^a^	0.051/37.4	–	1.00 (0.87, 1.15)^a^	<0.001/71.4	–	0.94 (0.78, 1.13)	0.253/16.5	–	1.00 (0.88, 1.12)^a^	<0.001/70.8	–
Indian		3 (993/346)	0.99 (0.70, 1.40)	0.144/48.5	–	0.96 (0.49, 1.89)	0.604/0.0	–	1.01 (0.73, 1.40)	0.187/40.3	–	1.07 (0.66, 1.75)	0.933/0.0	–	1.02 (0.80, 1.32)	0.390/0.0	–
Mixed		1 (578/390)	1.05 (0.78, 1.41)	–	–	1.07 (0.46, 2.49)	–	–	1.05 (0.79, 1.40)	–	–	1.05 (0.45, 2.45)	–	–	1.05 (0.81, 1.35)	–	–
Geographic region																	
Asia		9 (1,528/1,057)	0.76 (0.48, 1.22)^a^	<0.001/72.8	–	0.71 (0.47, 1.06)	0.141/34.6	–	–	<0.001/75.1	–	0.84 (0.59, 1.20)	0.578/0.0	–	0.82 (0.59, 1.15)^a^	<0.001/71.6	–
Europe		11 (8,369/9,417)	1.05 (0.94, 1.18)^a^	0.036/48.3	–	1.05 (0.84, 1.30)	0.305/14.6	–	1.05 (0.94, 1.18)^a^	0.035/48.5	–	1.04 (0.83, 1.29)	0.326/12.4	–	1.05 (0.95, 1.16)^a^	0.045/46.3	–
North America		5 (2,325/2,421)	1.09 (0.75, 1.58)^a^	0.023/64.7	–	0.85 (0.58, 1.23)	0.447/0.0	–	1.09 (0.74, 1.62)^a^	0.010/70.1	–	0.85 (0.59, 1.24)	0.508/0.0	–	1.06 (0.76, 1.49)^a^	0.010/69.7	–
ER status																	
Negative	2 (121/300)		0.72 (0.39, 1.32)	–	–	3.59 (0.32, 40.14)	–	–	0.78 (0.43, 1.41)	–	–	**8.60 (2.97, 24.91)**	**0.430/0.0**	0.961	0.87 (0.51, 1.49)	–	–
Positive	2 (243/300)		1.23 (0.78, 1.94)	–	–	3.13 (0.32, 30.42)	–	–	1.27 (0.81, 2.00)	–	–	**10.32 (4.31, 24.72)**	**0.224/32.4**	0.265	1.27 (0.84, 1.92)	–	–
Tumor grade																	
Grade I	2 (79/796)		1.23 (0.65, 2.31)	–	–	0.71 (0.09, 5.46)	–	–	1.17 (0.63, 2.17)	–	–	–	0.009/85.5	–	1.10 (0.63, 1.90)	–	–
Grade II	2 (95/796)		0.92 (0.48, 1.78)	–	–	0.30 (0.02, 5.04)	–	–	0.82 (0.42, 1.58)	–	–	–	0.002/89.4	–	0.74 (0.40, 1.38)	–	–
Grade III	2 (80/796)		0.94 (0.47, 1.88)	–	–	0.69 (0.09, 5.33)	–	–	0.92 (0.47, 1.78)	–	–	–	0.025/80.1	–	0.90 (0.49, 1.64)	–	–
Menopausal status																	
Postmenopausal	3 (382/240)		0.65 (0.30, 1.41)^a^	0.139/54.3	–	0.98 (0.19, 5.15)	0.833/0.0	–	0.68 (0.33, 1.39)^a^	0.156/50.3	–	3.27 (0.47, 22.62)^a^	0.035/70.2	–	0.79 (0.52, 1.19)	0.219/33.9	–
Premenopausal	3 (201/270)		1.20 (0.72, 2.01)	0.631/0.0	–	3.00 (0.59, 15.30)	0.624/0.0	–	1.30 (0.79, 2.15)	0.678/0.0	–	**7.30 (1.54, 34.58)** **^a^**	**0.134/50.3**	0.998	1.36 (0.87, 2.14)	0.730/0.0	–
Age																	
<50 years	2 (513/528)		**0.57 (0.41, 0.78)**	–	0.924	0.62 (0.26, 1.53)	–	–	**0.58 (0.42, 0.78)**	–	0.900	–	<0.001/95.0	–	**0.62 (0.47, 0.82)**	**–**	0.954
≥50 years	2 (1058/961)		1.02 (0.82, 1.27)	–	–	1.05 (0.57, 1.94)	–	–	1.02 (0.82, 1.26)	–	–	–	<0.001/95.1	–	1.02 (0.84, 1.23)	–	–
Sensitivity analysis																	
Overall		6 (4,341/3,983)	1.01 (0.91, 1.11)	0.338/12.0	–	1.07 (0.79, 1.44)	0.580/0.0	–	1.01 (0.92, 1.12)	0.282/20.1	–	1.07 (0.80, 1.44)	0.625/0.0	–	1.01 (0.93, 1.11)	0.268/22.1	–
Ethnicity																	
Caucasian		5 (3,763/3,593)	1.00 (0.90, 1.11)	0.233/28.4	–	1.07 (0.78, 1.47)	0.435/0.0	–	1.00 (0.90, 1.12)	0.187/35.2	–	1.07 (0.78, 1.47)	0.480/0.0	–	1.01 (0.92, 1.11)	0.174/37.0	–
Mixed		1 (578/390)	1.05 (0.78, 1.41)	–	–	1.07 (0.46, 2.49)	–	–	1.05 (0.79, 1.40)	–		1.05 (0.45, 2.45)	–	–	1.05 (0.81, 1.35)	–	–
Geographic region																	
Europe		5 (3,763/3,593)	1.00 (0.90, 1.11)	0.233/28.4	–	1.07 (0.78, 1.47)	0.435/0.0	–	1.00 (0.90, 1.12)	0.187/35.2	–	1.07 (0.78, 1.47)	0.480/0.0	–	1.01 (0.92, 1.11)	0.174/37.0	–
North America		1 (578/390)	1.05 (0.78, 1.41)	–	–	1.07 (0.46, 2.49)	–	–	1.05 (0.79, 1.40)	–	–	1.05 (0.45, 2.45)	–	–	1.05 (0.81, 1.35)	–	–

a Random-effects model was used in the pooled data.

Note: The bold values indicate significant results.

### Results of Published Meta-Analyses

The results of the published meta-analyses on the association between *TP53* codon 72, IVS3 16 bp, and IVS6+62A > G polymorphisms and BC risk in different ethnic groups are shown in Supplementary Table S5 and the cells highlighted with red color indicated significant results. Two studies (Dunning et al., 1999; Diakite et al., 2020b) showed that the codon 72 polymorphism increased BC risk in overall analysis. In the ethnic subgroup analyses of the association between codon 72 polymorphism and BC risk, one study (Gonçalves et al., 2014) found that the polymorphism significantly increased BC risk in Asians, and another study (Diakite et al., 2020b) indicated an obviously increased BC risk in Caucasians. With respect to the *TP53* IVS3 16 bp polymorphism, 6 studies (Dunning et al., 1999; Hu et al., 2010a; Hu et al., 2010b; He et al., 2011; Sagne et al., 2013; Wu et al., 2013; Diakite et al., 2020a) found an obviously increased BC risk in overall analysis. No published meta-analyses found significant association between IVS6+62A > G polymorphism and BC risk. However, when we used BFDP correction, the association of BC risk remained significant only in the overall analysis (GG vs. CC: BFDP = 0.738), matching subgroup (GG vs. CC: BFDP = 0.173; GG vs. CC + CG: BFDP = 0.447), and tumor size below 2 cm subgroup (GG vs. CC: BFDP = 0.088; GG + CG vs. CC: BFDP = 0.730; GG vs. CC + CG: BFDP = 0.311) of *TP53* IVS3 16 bp polymorphism. More importantly, none of these published meta-analyses pooled clinical data of the patients and the polymorphisms for more deeper insights into BC risk were still unknown.

## Discussion

*TP53* was first discovered in 1979, and was initially thought to be an oncogene (DeLeo et al., 1979; Bishop, 1985; Finlay et al., 1989). It was later realized that *TP53* was one of the tumor-suppressor genes most associated with human tumorigenesis so far (Donehower et al., 2019). In brief, when DNA damage was detected, *TP53* slowed down the cell cycle to allow the cell to repair the damage, and it induced the cell apoptosis when the damage was too severe. An experiment using 16 distinct genetically engineered mouse models of BC found that the loss of *TP53* in cancer cells induced the secretion of WNT ligand, which in turn stimulated the increase in IL-1β produced by tumor-associated macrophages, and further drove systemic inflammation and ultimately promoted the progression of metastasis (Wellenstein et al., 2019). These new studies demonstrated the important role of the *TP53* gene in BC suppression. At this point, with a total of 99 articles filtered out, we performed the current study and provided some evidence for the association between *TP53* codon 72, IVS3 16 bp, and IVS6+62A > G polymorphisms and BC risk.

In total, when analyzing *TP53* codon 72 polymorphism, the overall analysis showed that codon 72 polymorphism did not affect BC risk. In subgroup analyses, the combined results of studies in HWE-compliant and HWE in violation groups showed opposite conclusions. The matched studies subgroup, HWE-compliant subgroup, and studies with controls of BV source subgroup showed that codon 72 polymorphism significantly increased BC risk, whereas no significant results were found in other subgroup analyses as well as final sensitivity analyses, indicating that pooled data of relatively high-quality studies tend to suggest that codon 72 polymorphism increased BC risk. We did not find any susceptibility of BC with codon 72 polymorphism in any ethnicity or region. Although 99 studies have described the association between codon 72 polymorphism and BC risk, more high-quality studies are needed to draw a more convincing conclusion. For example, significant heterogeneity was found in the analysis of studies with controls of NR source subgroup (including 25 studies), with *I* ^*2*^ > 75% in all five genetic models, which indicated that quantitative synthesis mixed with low-quality data would affect the credibility of the final result. In overall analysis, *TP53* IVS3 16 bp polymorphism significantly increased BC risk, especially in Caucasians, Indians, and women living in Asia and Europe. Additionally, in stratified analyses of the subgroups, matched studies, neither blinding nor quality control, blinding and/or quality control, HWE-compliant studies, controls of PB source, controls of BV source, and controls of NR source once again proved the association between IVS3 16 bp polymorphism and BC risk. In further sensitivity analysis, IVS3 16 bp polymorphism was again found to be associated with BC risk in the overall analysis as well as Caucasians and women in European subgroups. For *TP53* IVS6+62A > G polymorphism, there were no significant results to demonstrate an association between IVS6+62A > G polymorphism and BC risk, except that a significantly increased BC risk was found in controls of BV source subgroup analysis.

In the current meta-analysis, it was carried out with data collection and statistics for *TP53* polymorphisms on the clinical characteristics of BC (Tables 2–4 and Supplementary Table S7). The codon 72 polymorphism was relatively less expressed in PR-negative patients, but did not show a significant association for the patients with different statuses of ER and HER2. Reduced mutation frequency of codon 72 polymorphism was found in the patients with age less than 50 years and tumor localized in right. Codon 72 polymorphism was significantly increased in patients with negative distant metastasis, suggesting that codon 72 polymorphism may inhibit distant metastasis of tumor. The expression of *TP53* codon 72 polymorphism and IVS3 16 bp polymorphism was significantly increased in BC grade I. Increased expression of *TP53* IVS3 16 bp polymorphism was seen in all tumor stages. The expression of *TP53* IVS3 16 bp polymorphism was significantly improved in tumor size smaller than 2 cm, in ductal carcinoma subtypes, and in ER-negative BC, respectively. Only one study (Eskandari-Nasab et al., 2015), about IVS3 16 bp polymorphism in different statuses of HER2, reported that IVS3 16 bp polymorphism was frequent in HER2-positive patients; however, this conclusion is not affirmative because the control group of the sample did not comply with HWE as well as other limitations in the studied procedures. Owing to the limited number of clinical studies on IVS6+62A > G polymorphism, the pooled result of three studies showed increased IVS6+62A > G polymorphism in premenopausal women, while the data from one study (Sprague et al., 2007) suggested IVS6+62A > G polymorphism associated with low risk in women younger than 50 years of age.

Given the vast amount of genomic data currently being generated, Wakefield. (2007) proposed an accurate Bayesian approach to measure false-positive reports in genetic epidemiology studies. Using BFDP for the correction, of all the significant results we found above, only the association between *TP53* IVS3 16 bp polymorphism and BC risk was observed in overall analysis (GG vs. CC: BFDP = 0.738), matched studies subgroup (GG vs. CC: BFDP = 0.173; GG vs. CC + CG: BFDP = 0.447), and tumor size below 2 cm (GG vs. CC: BFDP = 0.088; GG + CG vs. CC: BFDP = 0.730; GG vs. CC + CG: BFDP = 0.311). However, further correcting the significant results of the sensitivity analysis with BFDP, there was no significant correlation in overall analysis and any subgroup analyses. On account of the limitation of sufficient number of studies, we could not carry out the sensitivity analysis on clinical characteristics and the results should only be interpreted as an indication that is ,the results of *TP53* codon 72, IVS3 16 bp and IVS6+62A > G polymorphisms on BC clinical presentations. Hence, there are no reliable results to prove the association between *TP53* codon 72, IVS3 16 bp, and IVS6+62A > G polymorphisms and BC risk.

Supplementary Table S5 shows the published meta-analysis results of the association between *TP53* codon 72, IVS3 16 bp and IVS6+62A > G polymorphisms and BC risk. Published meta-analyses (Dunning et al., 1999; Gonçalves et al., 2014; Diakite et al., 2020b) indicated that the *TP53* codon 72 polymorphism significantly increased BC risk in overall analysis, Asians, Caucasians (cells highlighted with red color in Supplementary Table S5). Previous meta-analyses (Dunning et al., 1999; Hu et al., 2010a; Hu et al., 2010b; He et al., 2011; Sagne et al., 2013; Wu et al., 2013; Diakite et al., 2020a) had only reported *TP53* IVS3 16 bp polymorphism with an increased BC risk in overall analysis and only one study (Wu et al., 2013) performed the analyses stratified by ethnicity. Three published meta-analyses (Dunning et al., 1999; Hu et al., 2010b; He et al., 2011) showed no significant association between IVS6+62A > G polymorphism and BC susceptibility. It could be seen that there were significant inconsistencies in ethnic classification in published meta-analyses in studies from the United States, India, Egypt, Pakistan, Tunisia, Iran, Arabia, and Brazil (cells colored in red in Supplementary Tables 2–4). In addition, published meta-analyses involved some studies with overlapping data and many unnecessary data. Moreover, no study adjusted positive results for multiple comparisons by using correction tools, such as BFDP.

Published meta-analyses with largest sample size were performed in 2013 to detect the relationship of *TP53* codon 72 (59 studies including 29,801 cases and 35,436 controls) and IVS3 16 bp (19 studies including 4,479 cases and 4,683 controls), and in 2011 for IVS6+62A > G (14 studies including 8,787 cases and 9,869 controls) with BC risk (He et al., 2011; Dahabreh et al., 2013; Wu et al., 2013). The number of studies and sample size in the current meta-analysis (99 studies including 43,951 BC cases and 48,479 controls for codon 72, 35 studies including 8,705 BC cases and 7,516 controls for IVS3 16 bp, 25 studies including 12,222 BC cases and 12,895 controls for IVS6+62A > G) were larger than published meta-analyses. Compared to the current study, previous studies had several limitations. First, except for two articles (Francisco et al., 2011; Dahabreh et al., 2013) that established the epidemiological design criteria, none of the previous meta-analyses carried out literature quality assessment. Second, three meta-analyses (Dunning et al., 1999; Suspitsin et al., 2003; Gonçalves et al., 2014) did not report HWE of the included studies, and only four pieces (Zhuo et al., 2009; He et al., 2011; Ma et al., 2011; Cheng et al., 2012) excluded the studies that HWE in violation. Furthermore, only four previous meta-analyses (He et al., 2011; Cheng et al., 2012; Dahabreh et al., 2013; Hou et al., 2013) checked the duplicate of studies. Third, all previous meta-analyses (Dunning et al., 1999; Suspitsin et al., 2003; Zhuo et al., 2009; Hu et al., 2010a; Hu et al., 2010b; Zhang et al., 2010; Francisco et al., 2011; He et al., 2011; Ma et al., 2011; Cheng et al., 2012; Dahabreh et al., 2013; Hou et al., 2013; Sagne et al., 2013; Wu et al., 2013; Gonçalves et al., 2014; Diakite et al., 2020a; Diakite et al., 2020b) did not adjust positive results for multiple comparisons and did not conduct subgroup analysis with clinical presentations. Fourth, some published meta-analyses did not perform sensitivity analyses. In addition, included studies in published meta-analyses were incomplete due to improper retrieval strategies and a large number of updated original studies (Supplementary Tables 2–4). Finally, there were significant inconsistencies in the ethnic classification, especially for those from the United States, India, Brazil (the blue cells in Supplementary Tables 2–4).

Therefore, we performed an updated meta-analysis to further explore the association between *TP53* codon 72, IVS3 16 bp and IVS6+62A > G polymorphisms and BC risk. In the current meta-analysis, we used a larger sample size. In addition, we carried out a quality assessment of the relevant studies and considered the epidemiological characteristics of the included studies in this meta-analysis (Table 1 and Supplementary Tables 6, 7). Moreover, meta-regression analysis has been used to explore the source of heterogeneity from 13 items (including in geographic region, ethnicity, sample size, source of controls, source of cases, ascertainment of cancer, ascertainment of control, matching, source of genotyping material of case, whether genotyping was done blindly and/or quality control, HWE, association assessment with appropriate statistics and adjustment for confounders and quality score). Furthermore, we performed stratified analyses based on the epidemiological characteristics of the studies, especially sensitivity analyses that selected high-quality studies after comprehensive consideration of the processes of studies, with high composite scores and HWE-compliant (to avoid random errors and confounding bias that may distort the results of molecular epidemiological studies). In addition, subgroup analysis with clinical presentation was carried out in the present meta-analysis. Finally, the BFDP method has been used to correct significant results.

Although multiple strategies were used to ameliorate the issues of previous studies, there were still some limitations in this study. First, only published articles were browsed, so the omission of some research results may be inevitable. Second, there were less studies involved in some subgroup analyses, for example, there are only two studies for *TP53* codon 72 polymorphism and BC risk in African and only three studies on IVS3 16 bp polymorphism and BC risk in Asian, and there was only one study on IVS6+62A > G polymorphism and BC risk in African and Asian, respectively. In addition, high-quality studies were inadequate for sensitivity analysis of clinical presentations in this study. Third, the rating scales of research characteristics (Supplementary Table S1) that were used in this meta-analysis still have some defects. Although this assessment criteria had been used before (Klug et al., 2009; Thakkinstian et al., 2011), this rating scale may not be detailed enough, and the formulating score was simply calculated without weighted according to the importance of each item. Fourth, Table 1 displays the characters proportion of included studies in this meta-analysis included some of low-quality studies with small samples accounted for a certain proportion, which may affect the results of the overall analysis. Therefore, more accurate analysis should be carried out when sufficient data become available in the future.

## Conclusion

In summary, there are no robust significant results to confirm the association between *TP53* codon 72, IVS3 16 bp and IVS6+62A > G polymorphisms and BC risk. Other evidence about the increased BC risk associated with these three polymorphisms may most likely be due to false-positive results. Significant associations should be explained with caution and it is crucial that future analyses should be based on the quality of studies to effectively identify the impact of genetic variants on BC risk, particularly for the combined effects, such as gene–body status and gene–environment. The clinical findings presented in this meta-analysis are suggestive simply for the clinic, and more reliable and meaningful conclusions will depend on the development of more high-quality original studies and the statistical analysis for additional data in the future.

## Data Availability

The datasets presented in this study can be found in online repositories. The names of the repository/repositories and accession number(s) can be found in the article/Supplementary Material.

## Abbreviations

BC, breast cancer; BFDP, the Bayesian false-discovery probability; BV, blood donors or volunteers; CIs, confidence intervals; CNKI, China national knowledge infrastructure; ER, estrogen receptor; HB, hospital-based; HER2, human epidermal growth factor receptor 2; HWE, the Hardy-Weinberg equilibrium; IARC, international agency for research on cancer; NR, not reported; ORs, odds ratios; PB, population-based; PR, progesterone receptor; PRISMA, the preferred reporting items for systematic reviews and meta-analyses guidelines; SNPs, since single nucleotide polymorphisms.
