# Supplementary material for: Association Between the TP53 Polymorphisms and Breast Cancer Risk: An Updated Meta-Analysis
Source: Front Genet. 2022 Apr 27;13:807466. doi: 10.3389/fgene.2022.807466 (PMC9091657; doi:10.3389/fgene.2022.807466)
Supplement: Supplementary file 1 [file DataSheet7.PDF]

| Supplemental Table 7. Data on *TP53* polymorphisms based on clinicopathological features of BC patients within the present meta-analyses | | | | | | | | | | | | | | |
|---|---|---|---|---|---|---|---|---|---|---|---|---|---|---|
| No. | First Author/Year | Country | Geographic region | Ethnicity | Sample size (case/control) | Source of controls | Genotypes distribution of TP53 codon 72 | | | | | | HWE | Quality score |
| | | | | | | | Cases | | | Controls | | | | |
| | | | | | | | CC | CG | GG | CC | CG | GG | | |
| **ER status** | | | | | | | | | | | | | | |
| 1 | Icen-Taskin 2020 | Turkey | Asia | Caucasian | 46/96 | Positive | 20 | 14 | 12 | 32 | 47 | 17 | 0.9713 | 11 |
| 2 | Icen-Taskin 2020 | Turkey | Asia | Caucasian | 37/96 | Negative | 15 | 10 | 12 | 32 | 47 | 17 | 0.9713 | 11 |
| 3 | Ayoubi 2018 | Morocco | Africa | Caucasian | 25/126 | Positive | 14 | 6 | 5 | 65 | 46 | 15 | 0.1344 | 13 |
| 4 | Ayoubi 2018 | Morocco | Africa | Caucasian | 16/126 | Negative | 5 | 6 | 5 | 65 | 46 | 15 | 0.1344 | 13 |
| 5 | Hossain 2017 | Bangladesh | Asia | Indian | 68/125 | Positive | 27 | 23 | 18 | 61 | 51 | 13 | 0.6318 | 15 |
| 6 | Hossain 2017 | Bangladesh | Asia | Indian | 45/125 | Negative | 23 | 15 | 7 | 61 | 51 | 13 | 0.6318 | 15 |
| 7 | Almeida 2016 | Brazil | South America | Mixed | 132/205 | Positive | 50 | 62 | 20 | 85 | 87 | 33 | 0.1837 | 9 |
| 8 | Almeida 2016 | Brazil | South America | Mixed | 53/205 | Negative | 26 | 18 | 9 | 85 | 87 | 33 | 0.1837 | 9 |
| 9 | Proestling 2012 | Austria | Europe | Caucasian | 196/220 | Positive | 86 | 95 | 15 | 125 | 87 | 8 | 0.1272 | 13 |
| 10 | Proestling 2012 | Austria | Europe | Caucasian | 58/220 | Negative | 30 | 24 | 4 | 125 | 87 | 8 | 0.1272 | 13 |
| 11 | Cherdyntseva 2012 | Russia | Europe | Caucasian | 187/275 | Positive | 92 | 83 | 12 | 148 | 100 | 27 | 0.1037 | 17 |
| 12 | Cherdyntseva 2012 | Russia | Europe | Caucasian | 102/275 | Negative | 49 | 43 | 10 | 148 | 100 | 27 | 0.1037 | 17 |
| 13 | Yoshimoto 2011 | Japan | Asia | Asian | 621/258 | Positive | 253 | 290 | 78 | 111 | 106 | 41 | 0.0695 | 14 |
| 14 | Yoshimoto 2011 | Japan | Asia | Asian | 140/258 | Negative | 51 | 66 | 23 | 111 | 106 | 41 | 0.0695 | 14 |
| 15 | Alawadi 2011 | Arabia | Asia | Caucasian | 76/188 | Positive | 14 | 60 | 2 | 50 | 112 | 26 | 0.0038 | 10 |
| 16 | Alawadi 2011 | Arabia | Asia | Caucasian | 85/188 | Negative | 26 | 57 | 2 | 50 | 112 | 26 | 0.0038 | 10 |
| 17 | Kara 2010 | Turkey | Asia | Caucasian | 92/169 | Positive | 41 | 44 | 7 | 72 | 80 | 17 | 0.4439 | 14 |
| 18 | Kara 2010 | Turkey | Asia | Caucasian | 38/169 | Negative | 20 | 16 | 2 | 72 | 80 | 17 | 0.4439 | 14 |
| 19 | Akkiprik 2009 | Turkey | Asia | Caucasian | 45/107 | Positive | 36 | | 9 | 46 | 49 | 12 | 0.8462 | 12 |
| 20 | Akkiprik 2009 | Turkey | Asia | Caucasian | 19/107 | Negative | 13 | | 6 | 46 | 49 | 12 | 0.8462 | 12 |
| 21 | Damin 2006 | Brazil | South America | Mixed | 82/202 | Positive | 48 | 32 | 2 | 70 | 111 | 21 | 0.0171 | 10 |
| 22 | Damin 2006 | Brazil | South America | Mixed | 36/202 | Negative | 16 | 16 | 4 | 70 | 111 | 21 | 0.0171 | 10 |
| 23 | Tommiska 2005 | Finland | Europe | Caucasian | 671/733 | Positive | 347 | 276 | 48 | 403 | 278 | 52 | 0.6689 | 15 |
| 24 | Tommiska 2005 | Finland | Europe | Caucasian | 181/733 | Negative | 104 | 62 | 15 | 403 | 278 | 52 | 0.6689 | 15 |
| 25 | Noma 2004 | Japan | Asia | Asian | 107/218 | Positive | 48 | 35 | 24 | 111 | 76 | 31 | 0.0041 | 14 |
| 26 | Noma 2005 | Japan | Asia | Asian | 75/218 | Negative | 40 | 30 | 5 | 111 | 76 | 31 | 0.0041 | 15 |
| 27 | Papadakis 2000 | Greece | Europe | Caucasian | 30/59 | Positive | 18 | 6 | 6 | 12 | 41 | 6 | 0.0019 | 2 |
| 28 | Papadakis 2000 | Greece | Europe | Caucasian | 26/59 | Negative | 17 | 4 | 5 | 12 | 41 | 6 | 0.0019 | 2 |
| **PR status** | | | | | | | | | | | | | | |
| 1 | Icen-Taskin 2020 | Turkey | Asia | Caucasian | 44/96 | Positive | 22 | 14 | 8 | 32 | 47 | 17 | 0.9713 | 11 |
| 2 | Icen-Taskin 2020 | Turkey | Asia | Caucasian | 39/96 | Negative | 13 | 10 | 16 | 32 | 47 | 17 | 0.9713 | 11 |
| 3 | Ayoubi 2018 | Morocco | Africa | Caucasian | 42/126 | Positive | 23 | 13 | 6 | 65 | 46 | 15 | 0.1344 | 13 |
| 4 | Ayoubi 2018 | Morocco | Africa | Caucasian | 30/126 | Negative | 18 | 6 | 6 | 65 | 46 | 15 | 0.1344 | 13 |
| 5 | Almeida 2016 | Brazil | South America | Mixed | 111/205 | Positive | 42 | 52 | 17 | 85 | 87 | 33 | 0.1837 | 9 |
| 6 | Almeida 2016 | Brazil | South America | Mixed | 72/205 | Negative | 34 | 26 | 12 | 85 | 87 | 33 | 0.1837 | 9 |
| 7 | Proestling 2012 | Austria | Europe | Caucasian | 137/220 | Positive | 56 | 70 | 11 | 125 | 87 | 8 | 0.1272 | 13 |
| 8 | Proestling 2012 | Austria | Europe | Caucasian | 117/220 | Negative | 60 | 49 | 8 | 125 | 87 | 8 | 0.1272 | 13 |
| 9 | Alawadi 2011 | Arabia | Asia | Caucasian | 78/188 | Positive | 17 | 58 | 3 | 50 | 112 | 26 | 0.0038 | 10 |
| 10 | Alawadi 2011 | Arabia | Asia | Caucasian | 79/188 | Negative | 21 | 57 | 1 | 50 | 112 | 26 | 0.0038 | 10 |
| 11 | Akkiprik 2009 | Turkey | Asia | Caucasian | 41/107 | Positive | 32 | | 9 | 46 | 49 | 12 | 0.8462 | 12 |
| 12 | Akkiprik 2009 | Turkey | Asia | Caucasian | 21/107 | Negative | 15 | | 6 | 46 | 49 | 12 | 0.8462 | 12 |
| 13 | Damin 2006 | Brazil | South America | Mixed | 74/202 | Positive | 42 | 29 | 3 | 70 | 111 | 21 | 0.0171 | 10 |
| 14 | Damin 2006 | Brazil | South America | Mixed | 44/202 | Negative | 22 | 19 | 3 | 70 | 111 | 21 | 0.0171 | 10 |
| 15 | Tommiska 2005 | Finland | Europe | Caucasian | 580/733 | Positive | 293 | 244 | 43 | 403 | 278 | 52 | 0.6689 | 15 |
| 16 | Tommiska 2005 | Finland | Europe | Caucasian | 273/733 | Negative | 158 | 95 | 20 | 403 | 278 | 52 | 0.6689 | 15 |
| 17 | Papadakis 2000 | Greece | Europe | Caucasian | 31/59 | Positive | 18 | 7 | 6 | 12 | 41 | 6 | 0.0019 | 2 |
| 18 | Papadakis 2000 | Greece | Europe | Caucasian | 25/59 | Negative | 17 | 3 | 5 | 12 | 41 | 6 | 0.0019 | 2 |
| **HER-2 status** | | | | | | | | | | | | | | |
| 1 | Icen-Taskin 2020 | Turkey | Asia | Caucasian | 42/96 | Positive | 17 | 13 | 12 | 32 | 47 | 17 | 0.9713 | 11 |
| 2 | Icen-Taskin 2020 | Turkey | Asia | Caucasian | 41/96 | Negative | 18 | 11 | 12 | 32 | 47 | 17 | 0.9713 | 11 |
| 3 | Ayoubi 2018 | Morocco | Africa | Caucasian | 19/126 | Positive | 11 | 5 | 3 | 65 | 46 | 15 | 0.1344 | 13 |
| 4 | Ayoubi 2018 | Morocco | Africa | Caucasian | 20/126 | Negative | 8 | 5 | 7 | 65 | 46 | 15 | 0.1344 | 13 |
| 5 | Yadav 2016 | India | Asia | Indian | 54/100 | Positive | 16 | 23 | 15 | 47 | 42 | 11 | 0.7269 | 13 |
| 6 | Yadav 2016 | India | Asia | Indian | 46/100 | Negative | 10 | 32 | 4 | 47 | 42 | 11 | 0.7269 | 13 |
| 7 | Almeida 2016 | Brazil | South America | Mixed | 30/205 | Positive | 11 | 12 | 7 | 85 | 87 | 33 | 0.1837 | 9 |
| 8 | Almeida 2016 | Brazil | South America | Mixed | 155/205 | Negative | 65 | 68 | 22 | 85 | 87 | 33 | 0.1837 | 9 |
| 9 | Proestling 2012 | Austria | Europe | Caucasian | 51/220 | Positive | 30 | 16 | 5 | 125 | 87 | 8 | 0.1272 | 13 |
| 10 | Proestling 2012 | Austria | Europe | Caucasian | 200/220 | Negative | 85 | 101 | 14 | 125 | 87 | 8 | 0.1272 | 13 |
| 11 | Alawadi 2011 | Arabia | Asia | Caucasian | 94/188 | Positive | 21 | 69 | 4 | 50 | 112 | 26 | 0.0038 | 10 |
| 12 | Alawadi 2011 | Arabia | Asia | Caucasian | 60/188 | Negative | 17 | 43 | 0 | 50 | 112 | 26 | 0.0038 | 10 |
| 13 | Damin 2006 | Brazil | South America | Mixed | 32/202 | Positive | 12 | 18 | 2 | 70 | 111 | 21 | 0.0171 | 10 |
| 14 | Damin 2006 | Brazil | South America | Mixed | 86/202 | Negative | 52 | 30 | 4 | 70 | 111 | 21 | 0.0171 | 10 |
| **Tumor stage** | | | | | | | | | | | | | | |
| 1 | Papadakis 2000 | Greece | Europe | Caucasian | 24/59 | Stage I | 15 | 3 | 6 | 12 | 41 | 6 | 0.0019 | 2 |
| 2 | Papadakis 2000 | Greece | Europe | Caucasian | 25/59 | Stage II | 14 | 6 | 5 | 12 | 41 | 6 | 0.0019 | 2 |
| 3 | Papadakis 2000 | Greece | Europe | Caucasian | 7/59 | Stage III | 6 | 1 | 0 | 12 | 41 | 6 | 0.0019 | 2 |
| 4 | Damin 2006 | Brazil | South America | Mixed | 33/202 | Stage 0 or I | 20 | 12 | 1 | 70 | 111 | 21 | 0.0171 | 10 |
| 5 | Damin 2006 | Brazil | South America | Mixed | 52/202 | Stage II | 29 | 22 | 1 | 70 | 111 | 21 | 0.0171 | 10 |
| 6 | Damin 2006 | Brazil | South America | Mixed | 33/202 | Stage III or IV | 15 | 14 | 4 | 70 | 111 | 21 | 0.0171 | 10 |
| 7 | Kara 2010 | Turkey | Asia | Caucasian | 14/169 | Stage I | 3 | 11 | 0 | 72 | 80 | 17 | 0.4439 | 14 |
| 8 | Kara 2010 | Turkey | Asia | Caucasian | 50/169 | Stage II | 32 | 16 | 2 | 72 | 80 | 17 | 0.4439 | 14 |
| 9 | Kara 2010 | Turkey | Asia | Caucasian | 61/169 | Stage III | 31 | 24 | 6 | 72 | 80 | 17 | 0.4439 | 14 |
| 10 | Kara 2010 | Turkey | Asia | Caucasian | 5/169 | Stage IV | 2 | 3 | 0 | 72 | 80 | 17 | 0.4439 | 14 |
| 11 | Alawadi 2011 | Arabia | Asia | Caucasian | 9/188 | Stage I | 1 | 8 | 0 | 50 | 112 | 26 | 0.0038 | 10 |
| 12 | Alawadi 2011 | Arabia | Asia | Caucasian | 71/188 | Stage II | 14 | 56 | 1 | 50 | 112 | 26 | 0.0038 | 10 |
| 13 | Alawadi 2011 | Arabia | Asia | Caucasian | 75/188 | Stage III | 22 | 53 | 0 | 50 | 112 | 26 | 0.0038 | 10 |
| 14 | Alawadi 2011 | Arabia | Asia | Caucasian | 30/188 | Stage IV | 9 | 18 | 3 | 50 | 112 | 26 | 0.0038 | 10 |
| 15 | Guleria 2012 | India | Asia | Indian | 7/80 | Stage I | 1 | 6 | 0 | 27 | 32 | 21 | 0.0804 | 14 |
| 16 | Guleria 2012 | India | Asia | Indian | 42/80 | Stage II | 7 | 25 | 10 | 27 | 32 | 21 | 0.0804 | 14 |
| 17 | Guleria 2012 | India | Asia | Indian | 25/80 | Stage III | 3 | 12 | 10 | 27 | 32 | 21 | 0.0804 | 14 |
| 18 | Guleria 2012 | India | Asia | Indian | 6/80 | Stage IV | 0 | 4 | 2 | 27 | 32 | 21 | 0.0804 | 14 |
| 19 | Proestling 2012 | Austria | Europe | Caucasian | 112/220 | Stage 0 or I | 54 | 49 | 9 | 125 | 87 | 8 | 0.1272 | 13 |
| 20 | Proestling 2012 | Austria | Europe | Caucasian | 63/220 | Stage II | 25 | 35 | 3 | 125 | 87 | 8 | 0.1272 | 13 |
| 21 | Proestling 2012 | Austria | Europe | Caucasian | 20/220 | Stage III or IV | 11 | 7 | 2 | 125 | 87 | 8 | 0.1272 | 13 |
| 22 | Almeida 2016 | Brazil | South America | Mixed | 22/205 | Stage I | 9 | 10 | 3 | 85 | 87 | 33 | 0.1837 | 9 |
| 23 | Almeida 2016 | Brazil | South America | Mixed | 80/205 | Stage II | 40 | 28 | 12 | 85 | 87 | 33 | 0.1837 | 9 |
| 24 | Almeida 2016 | Brazil | South America | Mixed | 75/205 | Stage III | 26 | 36 | 13 | 85 | 87 | 33 | 0.1837 | 9 |

Supplemental Table 7 continued

| | | | | | | | | | | | | | | |
|---|---|---|---|---|---|---|---|---|---|---|---|---|---|---|
| 25 | Almeida 2016 | Brazil | South America | Mixed | 8/205 | Stage IV | 3 | 5 | 0 | 85 | 87 | 33 | 0.1837 | 9 |
| 26 | Golmohammadi 2016 | Iran | Asia | Caucasian | 31/80 | Stage I | 9 | 22 | 0 | 51 | 15 | 14 | 0.0000 | 6 |
| 27 | Golmohammadi 2016 | Iran | Asia | Caucasian | 33/80 | Stage II | 7 | 26 | 0 | 51 | 15 | 14 | 0.0000 | 6 |
| 28 | Golmohammadi 2016 | Iran | Asia | Caucasian | 10/80 | Stage III | 8 | 1 | 1 | 51 | 15 | 14 | 0.0000 | 6 |
| 29 | Golmohammadi 2016 | Iran | Asia | Caucasian | 6/80 | Stage IV | 5 | 0 | 1 | 51 | 15 | 14 | 0.0000 | 6 |
| 30 | Pouladi 2020 | Iran | Asia | Caucasian | 29/179 | Stage I | 14 | 9 | 6 | 64 | 81 | 34 | 0.3572 | 9 |
| 31 | Pouladi 2020 | Iran | Asia | Caucasian | 66/179 | Stage II | 28 | 29 | 9 | 64 | 81 | 34 | 0.3572 | 9 |
| 32 | Pouladi 2020 | Iran | Asia | Caucasian | 81/179 | Stage III | 33 | 37 | 11 | 64 | 81 | 34 | 0.3572 | 9 |
| **Tumor grade** | | | | | | | | | | | | | | |
| 1 | Icen-Taskin 2020 | Turkey | Asia | Caucasian | 12/96 | Grade I | 3 | 6 | 3 | 32 | 47 | 17 | 0.9713 | 11 |
| 2 | Icen-Taskin 2020 | Turkey | Asia | Caucasian | 41/96 | Grade II | 17 | 11 | 13 | 32 | 47 | 17 | 0.9713 | 11 |
| 3 | Icen-Taskin 2020 | Turkey | Asia | Caucasian | 30/96 | Grade III | 14 | 9 | 7 | 32 | 47 | 17 | 0.9713 | 11 |
| 4 | Ayoubi 2018 | Morocco | Africa | Caucasian | 7/126 | Grade I | 2 | 3 | 2 | 65 | 46 | 15 | 0.1344 | 13 |
| 5 | Ayoubi 2018 | Morocco | Africa | Caucasian | 75/126 | Grade II | 34 | 25 | 16 | 65 | 46 | 15 | 0.1344 | 13 |
| 6 | Ayoubi 2018 | Morocco | Africa | Caucasian | 41/126 | Grade III | 19 | 12 | 10 | 65 | 46 | 15 | 0.1344 | 13 |
| 7 | Hossain 2017 | Bangladesh | Asia | Indian | 24/125 | Grade I | 10 | 8 | 6 | 61 | 51 | 13 | 0.6318 | 15 |
| 8 | Hossain 2017 | Bangladesh | Asia | Indian | 66/125 | Grade II | 30 | 21 | 15 | 61 | 51 | 13 | 0.6318 | 15 |
| 9 | Hossain 2017 | Bangladesh | Asia | Indian | 35/125 | Grade III | 14 | 13 | 8 | 61 | 51 | 13 | 0.6318 | 15 |
| 10 | Golmohammadi 2016 | Iran | Asia | Caucasian | 13/80 | Grade I | 6 | 7 | 0 | 51 | 15 | 14 | 0.0000 | 6 |
| 11 | Golmohammadi 2016 | Iran | Asia | Caucasian | 45/80 | Grade II | 11 | 34 | 0 | 51 | 15 | 14 | 0.0000 | 6 |
| 12 | Golmohammadi 2016 | Iran | Asia | Caucasian | 22/80 | Grade III | 12 | 8 | 2 | 51 | 15 | 14 | 0.0000 | 6 |
| 13 | Almeida 2016 | Brazil | South America | Mixed | 17/205 | Grade I | 3 | 8 | 6 | 85 | 87 | 33 | 0.1837 | 9 |
| 14 | Almeida 2016 | Brazil | South America | Mixed | 121/205 | Grade II | 49 | 56 | 16 | 85 | 87 | 33 | 0.1837 | 9 |
| 15 | Almeida 2016 | Brazil | South America | Mixed | 47/205 | Grade III | 25 | 15 | 7 | 85 | 87 | 33 | 0.1837 | 9 |
| 16 | Proestling 2012 | Austria | Europe | Caucasian | 43/220 | Grade I | 19 | 23 | 1 | 125 | 87 | 8 | 0.1272 | 13 |
| 17 | Proestling 2012 | Austria | Europe | Caucasian | 114/220 | Grade II | 47 | 55 | 12 | 125 | 87 | 8 | 0.1272 | 13 |
| 18 | Proestling 2012 | Austria | Europe | Caucasian | 88/220 | Grade III | 49 | 34 | 5 | 125 | 87 | 8 | 0.1272 | 13 |
| 19 | Akkiprik 2009 | Turkey | Asia | Caucasian | 23/107 | Grade I | 17 | | 6 | 46 | 49 | 12 | 0.8462 | 12 |
| 20 | Akkiprik 2009 | Turkey | Asia | Caucasian | 36/107 | Grade II | 28 | | 8 | 46 | 49 | 12 | 0.8462 | 12 |
| 21 | Akkiprik 2009 | Turkey | Asia | Caucasian | 25/107 | Grade III | 19 | | 6 | 46 | 49 | 12 | 0.8462 | 12 |
| 22 | Damin 2006 | Brazil | South America | Mixed | 12/202 | Grade I | 6 | 6 | 0 | 70 | 111 | 21 | 0.0171 | 10 |
| 23 | Damin 2006 | Brazil | South America | Mixed | 47/202 | Grade II | 24 | 21 | 2 | 70 | 111 | 21 | 0.0171 | 10 |
| 24 | Damin 2006 | Brazil | South America | Mixed | 31/202 | Grade III | 17 | 11 | 3 | 70 | 111 | 21 | 0.0171 | 10 |
| 25 | Tommiska 2005 | Finland | Europe | Caucasian | 221/733 | Grade I | 110 | 88 | 23 | 403 | 278 | 52 | 0.6689 | 15 |
| 26 | Tommiska 2005 | Finland | Europe | Caucasian | 349/733 | Grade II | 198 | 124 | 27 | 403 | 278 | 52 | 0.6689 | 15 |
| 27 | Tommiska 2005 | Finland | Europe | Caucasian | 239/733 | Grade III | 129 | 98 | 12 | 403 | 278 | 52 | 0.6689 | 15 |
| 28 | Noma 2004 | Japan | Asia | Asian | 152/218 | Grade I and II | 73 | 53 | 26 | 111 | 76 | 31 | 0.0041 | 14 |
| 29 | Noma 2004 | Japan | Asia | Asian | 27/218 | Grade III | 15 | 10 | 2 | 111 | 76 | 31 | 0.0041 | 14 |
| 30 | Papadakis 2000 | Greece | Europe | Caucasian | 8/59 | Grade I | 4 | 0 | 4 | 12 | 41 | 6 | 0.0019 | 2 |
| 31 | Papadakis 2000 | Greece | Europe | Caucasian | 42/59 | Grade II | 29 | 7 | 6 | 12 | 41 | 6 | 0.0019 | 2 |
| 32 | Papadakis 2000 | Greece | Europe | Caucasian | 6/59 | Grade III | 2 | 3 | 1 | 12 | 41 | 6 | 0.0019 | 2 |
| 33 | Själander 1996 | Sweden | Europe | Caucasian | 56/689 | Grade I | 22 | 27 | 7 | 375 | 253 | 61 | 0.0550 | 18 |
| 34 | Själander 1996 | Sweden | Europe | Caucasian | 59/689 | Grade II | 26 | 28 | 5 | 375 | 253 | 61 | 0.0550 | 18 |
| 35 | Själander 1996 | Sweden | Europe | Caucasian | 54/689 | Grade III | 30 | 15 | 9 | 375 | 253 | 61 | 0.0550 | 18 |
| **Tumor size** | | | | | | | | | | | | | | |
| 1 | Pouladi 2020 | Iran | Asia | Caucasian | 43/179 | T1 | 20 | 14 | 9 | 64 | 81 | 34 | 0.3572 | 9 |
| 2 | Pouladi 2020 | Iran | Asia | Caucasian | 93/179 | T2 | 39 | 41 | 13 | 64 | 81 | 34 | 0.3572 | 9 |
| 3 | Pouladi 2020 | Iran | Asia | Caucasian | 31/179 | T3 | 13 | 15 | 3 | 64 | 81 | 34 | 0.3572 | 9 |
| 4 | Pouladi 2020 | Iran | Asia | Caucasian | 9/179 | T4 | 4 | 5 | 0 | 64 | 81 | 34 | 0.3572 | 9 |
| 5 | Icen-Taskin 2020 | Turkey | Asia | Caucasian | 27/96 | T1 | 14 | 9 | 4 | 32 | 47 | 17 | 0.9713 | 11 |
| 6 | Icen-Taskin 2020 | Turkey | Asia | Caucasian | 37/96 | T2 | 17 | 8 | 12 | 32 | 47 | 17 | 0.9713 | 11 |
| 7 | Icen-Taskin 2020 | Turkey | Asia | Caucasian | 23/96 | T3 | 6 | 10 | 7 | 32 | 47 | 17 | 0.9713 | 11 |
| 8 | Ayoubi 2018 | Morocco | Africa | Caucasian | 10/126 | T1 | 4 | 5 | 1 | 65 | 46 | 15 | 0.1344 | 13 |
| 9 | Ayoubi 2018 | Morocco | Africa | Caucasian | 48/126 | T2 | 19 | 18 | 11 | 65 | 46 | 15 | 0.1344 | 13 |
| 10 | Ayoubi 2018 | Morocco | Africa | Caucasian | 27/126 | T3 | 12 | 9 | 6 | 65 | 46 | 15 | 0.1344 | 13 |
| 11 | Proestling 2012 | Austria | Europe | Caucasian | 132/220 | T1 | 64 | 57 | 11 | 125 | 87 | 8 | 0.1272 | 13 |
| 12 | Proestling 2012 | Austria | Europe | Caucasian | 56/220 | T2 | 19 | 33 | 4 | 125 | 87 | 8 | 0.1272 | 13 |
| 13 | Proestling 2012 | Austria | Europe | Caucasian | 11/220 | T3 and T4 | 6 | 4 | 1 | 125 | 87 | 8 | 0.1272 | 13 |
| 14 | Noma 2004 | Japan | Asia | Asian | 64/218 | T1 | 35 | 20 | 9 | 111 | 76 | 31 | 0.0041 | 14 |
| 15 | Noma 2004 | Japan | Asia | Asian | 111/218 | T2 | 49 | 43 | 19 | 111 | 76 | 31 | 0.0041 | 14 |
| **Menopausal status** | | | | | | | | | | | | | | |
| 1 | Noma 2004 | Japan | Asia | Asian | 58/88 | Pre | 32 | 17 | 9 | 46 | 31 | 11 | 0.1261 | 11 |
| 2 | Noma 2004 | Japan | Asia | Asian | 49/130 | Post | 16 | 18 | 15 | 65 | 45 | 20 | 0.0149 | 9 |
| 3 | Damin 2006 | Brazil | South America | Mixed | 50/202 | Pre | 26 | 23 | 1 | 70 | 111 | 21 | 0.0171 | 10 |
| 4 | Damin 2006 | Brazil | South America | Mixed | 63/202 | Post | 35 | 24 | 4 | 70 | 111 | 21 | 0.0171 | 10 |
| 5 | Singh 2008 | India | Asia | Indian | 34/69 | Pre | 16 | 13 | 5 | 22 | 39 | 8 | 0.1371 | 11 |
| 6 | Singh 2008 | India | Asia | Indian | 70/36 | Post | 30 | 32 | 8 | 6 | 26 | 4 | 0.0071 | 9 |
| 7 | Akkiprik 2009 | Turkey | Asia | Caucasian | 23/107 | Pre | 19 | | 4 | 46 | 49 | 12 | 0.8462 | 12 |
| 8 | Akkiprik 2009 | Turkey | Asia | Caucasian | 63/107 | Post | 45 | | 18 | 46 | 49 | 12 | 0.8462 | 12 |
| 9 | Song 2009 | China | Asia | Asian | 534/516 | Pre | 175 | 258 | 101 | 174 | 239 | 103 | 0.2052 | 15 |
| 10 | Song 2009 | China | Asia | Asian | 570/561 | Post | 164 | 286 | 120 | 175 | 269 | 117 | 0.4681 | 15 |
| 11 | Alawadi 2011 | Arabia | Asia | Caucasian | 51/188 | Pre | 15 | 33 | 3 | 50 | 112 | 26 | 0.0038 | 10 |
| 12 | Alawadi 2011 | Arabia | Asia | Caucasian | 27/188 | Post | 6 | 19 | 2 | 50 | 112 | 26 | 0.0038 | 10 |
| 13 | Koh 2011 | Singapore | Asia | Asian | 259/447 | Post | 71 | 141 | 47 | 130 | 212 | 105 | 0.3054 | 15 |
| 14 | Cherdyntseva 2012 | Russia | Europe | Caucasian | 140/142 | Pre | 59 | 63 | 18 | 78 | 54 | 10 | 0.8757 | 15 |
| 15 | Cherdyntseva 2012 | Russia | Europe | Caucasian | 247/127 | Post | 124 | 99 | 24 | 65 | 45 | 17 | 0.0509 | 15 |
| 16 | Proestling 2012 | Austria | Europe | Caucasian | 61/220 | Pre | 30 | 25 | 6 | 125 | 87 | 8 | 0.1272 | 13 |
| 17 | Proestling 2012 | Austria | Europe | Caucasian | 172/220 | Post | 78 | 83 | 11 | 125 | 87 | 8 | 0.1272 | 13 |
| 18 | Yadav 2016 | India | Asia | Indian | 44/100 | Pre | 11 | 25 | 8 | 47 | 42 | 11 | 0.7269 | 13 |
| 19 | Yadav 2016 | India | Asia | Indian | 56/100 | Post | 15 | 30 | 11 | 47 | 42 | 11 | 0.7269 | 13 |
| 20 | Hossain 2017 | Bangladesh | Asia | Indian | 68/125 | Pre | 32 | 24 | 12 | 61 | 51 | 13 | 0.6318 | 15 |
| 21 | Hossain 2018 | Bangladesh | Asia | Indian | 57/128 | Post | 22 | 18 | 17 | 62 | 52 | 14 | 0.6319 | 16 |
| 22 | Ayoubi 2018 | Morocco | Africa | Caucasian | 55/126 | Pre | 24 | 16 | 15 | 65 | 46 | 15 | 0.1344 | 13 |
| 23 | Ayoubi 2018 | Morocco | Africa | Caucasian | 69/126 | Post | 31 | 25 | 13 | 65 | 46 | 15 | 0.1344 | 13 |
| 24 | Akhter 2021 | India | Asia | Indian | 69/69 | Pre | 35 | 19 | 15 | 23 | 37 | 9 | 0.3249 | 9 |
| 25 | Akhter 2021 | India | Asia | Indian | 46/46 | Post | 26 | 6 | 14 | 8 | 34 | 4 | 0.0009 | 9 |
| **Age** | | | | | | | | | | | | | | |
| 1 | Pouladi 2020 | Iran | Asia | Caucasian | 96/179 | ≥45 years | 42 | 40 | 14 | 64 | 81 | 34 | 0.3572 | 9 |
| 2 | Pouladi 2020 | Iran | Asia | Caucasian | 104/179 | <45 years | 45 | 44 | 15 | 64 | 81 | 34 | 0.3572 | 9 |
| 3 | Ayoubi 2018 | Morocco | Africa | Caucasian | 113/126 | ≥40 years | 49 | 39 | 25 | 65 | 46 | 15 | 0.1344 | 13 |
| 4 | Ayoubi 2018 | Morocco | Africa | Caucasian | 12/126 | <40 years | 6 | 3 | 3 | 65 | 46 | 15 | 0.1344 | 13 |
| 5 | Hossain 2017 | Bangladesh | Asia | Indian | 104/125 | ≥40 years | 44 | 35 | 25 | 61 | 51 | 13 | 0.6318 | 15 |

Supplemental Table 7 continued

| | | | | | | | | | | | | | | |
|---|---|---|---|---|---|---|---|---|---|---|---|---|---|---|
| 6 | Hossain 2017 | Bangladesh | Asia | Indian | 21/125 | <40 years | 10 | 7 | 4 | 61 | 51 | 13 | 0.6318 | 15 |
| 7 | Yadav 2016 | India | Asia | Indian | 49/100 | ≥45 years | 11 | 27 | 11 | 47 | 42 | 11 | 0.7269 | 13 |
| 8 | Yadav 2016 | India | Asia | Indian | 51/100 | <45 years | 15 | 28 | 8 | 47 | 42 | 11 | 0.7269 | 13 |
| 9 | Golmohammadi 2016 | Iran | Asia | Caucasian | 73/80 | ≥45 years | 24 | 45 | 4 | 51 | 15 | 14 | 0.0000 | 6 |
| 10 | Golmohammadi 2016 | Iran | Asia | Caucasian | 87/80 | <45 years | 20 | 55 | 12 | 51 | 15 | 14 | 0.0000 | 6 |
| 11 | Almeida 2016 | Brazil | South America | Mixed | 171/205 | ≥40 years | 73 | 70 | 28 | 85 | 87 | 33 | 0.1837 | 9 |
| 12 | Almeida 2016 | Brazil | South America | Mixed | 17/205 | <40 years | 6 | 10 | 1 | 85 | 87 | 33 | 0.1837 | 9 |
| 13 | Alawadi 2011 | Arabia | Asia | Caucasian | 160/188 | ≥40 years | 40 | 116 | 4 | 50 | 112 | 26 | 0.0038 | 10 |
| 14 | Alawadi 2011 | Arabia | Asia | Caucasian | 54/188 | <40 years | 13 | 40 | 1 | 50 | 112 | 26 | 0.0038 | 10 |
| 15 | Kara 2010 | Turkey | Asia | Caucasian | 85/169 | ≥51 years | 46 | 35 | 4 | 72 | 80 | 17 | 0.4439 | 14 |
| 16 | Kara 2010 | Turkey | Asia | Caucasian | 93/169 | <51 years | 46 | 40 | 7 | 72 | 80 | 17 | 0.4439 | 14 |
| 17 | Akkiprik 2009 | Turkey | Asia | Caucasian | 64/107 | ≥51 years | 44 | | 20 | 46 | 49 | 12 | 0.8462 | 12 |
| 18 | Akkiprik 2009 | Turkey | Asia | Caucasian | 30/107 | <51 years | 26 | | 4 | 46 | 49 | 12 | 0.8462 | 12 |
| 19 | Sprague 2007 | USA | North America | Caucasian | 993/858 | ≥50 years | 545 | 382 | 66 | 480 | 326 | 52 | 0.7332 | 19 |
| 20 | Sprague 2007 | USA | North America | Caucasian | 489/420 | <50 years | 278 | 188 | 23 | 225 | 164 | 31 | 0.8821 | 19 |
| 21 | Franeková 2007 | Slovakia | Europe | Caucasian | 64/43 | ≥50 years | 36 | 23 | 5 | 21 | 20 | 2 | 0.3066 | 13 |
| 22 | Franeková 2007 | Slovakia | Europe | Caucasian | 27/113 | <50 years | 13 | 11 | 3 | 71 | 35 | 7 | 0.3497 | 13 |
| 23 | Damin 2006 | Brazil | South America | Mixed | 80/202 | ≥50 years | 41 | 34 | 5 | 70 | 111 | 21 | 0.0171 | 10 |
| 24 | Damin 2006 | Brazil | South America | Mixed | 38/202 | <50 years | 23 | 14 | 1 | 70 | 111 | 21 | 0.0171 | 10 |
| 25 | Papadakis 2000 | Greece | Europe | Caucasian | 42/59 | ≥51 years | 23 | 7 | 12 | 12 | 41 | 6 | 0.0019 | 2 |
| 26 | Papadakis 2000 | Greece | Europe | Caucasian | 14/59 | <51 years | 11 | 3 | 0 | 12 | 41 | 6 | 0.0019 | 2 |
| **Localization** | | | | | | | | | | | | | | |
| 1 | Pouladi 2020 | Iran | Asia | Caucasian | 102/179 | Left | 41 | 46 | 15 | 64 | 81 | 34 | 0.3572 | 9 |
| 2 | Pouladi 2020 | Iran | Asia | Caucasian | 98/179 | Right | 46 | 38 | 14 | 64 | 81 | 34 | 0.3572 | 9 |
| 3 | Icen-Taskin 2020 | Turkey | Asia | Caucasian | 47/96 | Right | 20 | 15 | 12 | 32 | 47 | 17 | 0.9713 | 11 |
| 4 | Icen-Taskin 2020 | Turkey | Asia | Caucasian | 48/96 | Left | 21 | 14 | 13 | 32 | 47 | 17 | 0.9713 | 11 |
| 5 | Icen-Taskin 2020 | Turkey | Asia | Caucasian | 1/96 | Right and left | 1 | 0 | 0 | 32 | 47 | 17 | 0.9713 | 11 |
| **Histological subtype** | | | | | | | | | | | | | | |
| 1 | Pouladi 2020 | Iran | Asia | Caucasian | 175/179 | IDC | 75 | 73 | 27 | 64 | 81 | 34 | 0.3572 | 9 |
| 2 | Pouladi 2020 | Iran | Asia | Caucasian | 7/179 | DCIS | 3 | 3 | 1 | 64 | 81 | 34 | 0.3572 | 9 |
| 3 | Pouladi 2020 | Iran | Asia | Caucasian | 9/179 | ILC | 5 | 4 | 0 | 64 | 81 | 34 | 0.3572 | 9 |
| 4 | Pouladi 2020 | Iran | Asia | Caucasian | 1/179 | Fibroadenoma | 1 | 0 | 0 | 64 | 81 | 34 | 0.3572 | 9 |
| 5 | Ayoubi 2018 | Morocco | Africa | Caucasian | 105/126 | IDC | 45 | 37 | 23 | 65 | 46 | 15 | 0.1344 | 13 |
| 6 | Ayoubi 2018 | Morocco | Africa | Caucasian | 8/126 | ILC | 7 | 1 | 0 | 65 | 46 | 15 | 0.1344 | 13 |
| 7 | Proestling 2012 | Austria | Europe | Caucasian | 148/220 | Ductal | 74 | 65 | 9 | 125 | 87 | 8 | 0.1272 | 13 |
| 8 | Proestling 2012 | Austria | Europe | Caucasian | 47/220 | Lobular | 18 | 24 | 5 | 125 | 87 | 8 | 0.1272 | 13 |
| 9 | Kara 2010 | Turkey | Asia | Caucasian | 150/169 | Ductal | 73 | 67 | 10 | 72 | 80 | 17 | 0.4439 | 14 |
| 10 | Kara 2010 | Turkey | Asia | Caucasian | 11/169 | Lobular | 7 | 4 | 0 | 72 | 80 | 17 | 0.4439 | 14 |
| 11 | Damin 2006 | Brazil | South America | Mixed | 90/202 | Ductal | 47 | 38 | 5 | 70 | 111 | 21 | 0.0171 | 10 |
| 12 | Damin 2006 | Brazil | South America | Mixed | 14/202 | Lobular | 9 | 5 | 0 | 70 | 111 | 21 | 0.0171 | 10 |
| 13 | Tommiska 2005 | Finland | Europe | Caucasian | 664/733 | Ductal | 361 | 263 | 40 | 403 | 278 | 52 | 0.6689 | 15 |
| 14 | Tommiska 2005 | Finland | Europe | Caucasian | 138/733 | Lobular | 65 | 55 | 18 | 403 | 278 | 52 | 0.6689 | 15 |
| 15 | Tommiska 2005 | Finland | Europe | Caucasian | 13/733 | Medullary | 8 | 3 | 2 | 403 | 278 | 52 | 0.6689 | 15 |
| 16 | Noma 2004 | Japan | Asia | Asian | 8/218 | DCIS | 5 | 3 | 0 | 111 | 76 | 31 | 0.0041 | 14 |
| 17 | Noma 2004 | Japan | Asia | Asian | 164/218 | IDC | 76 | 60 | 28 | 111 | 76 | 31 | 0.0041 | 14 |
| 18 | Noma 2004 | Japan | Asia | Asian | 8/218 | ILC | 6 | 2 | 0 | 111 | 76 | 31 | 0.0041 | 14 |
| **Lymph node** | | | | | | | | | | | | | | |
| 1 | Pouladi 2020 | Iran | Asia | Caucasian | 78/179 | N0 | 35 | 29 | 14 | 64 | 81 | 34 | 0.3572 | 9 |
| 2 | Pouladi 2020 | Iran | Asia | Caucasian | 40/179 | N1 | 16 | 21 | 3 | 64 | 81 | 34 | 0.3572 | 9 |
| 3 | Pouladi 2020 | Iran | Asia | Caucasian | 39/179 | N2 | 20 | 13 | 6 | 64 | 81 | 34 | 0.3572 | 9 |
| 4 | Pouladi 2020 | Iran | Asia | Caucasian | 32/179 | N3 | 9 | 19 | 4 | 64 | 81 | 34 | 0.3572 | 9 |
| 5 | Ayoubi 2018 | Morocco | Africa | Caucasian | 45/126 | Positive | 24 | 11 | 10 | 65 | 46 | 15 | 0.1344 | 13 |
| 6 | Ayoubi 2018 | Morocco | Africa | Caucasian | 78/126 | Negative | 31 | 29 | 18 | 65 | 46 | 15 | 0.1344 | 13 |
| 7 | Yadav 2016 | India | Asia | Indian | 59/100 | Positive | 17 | 30 | 12 | 47 | 42 | 11 | 0.7269 | 13 |
| 8 | Yadav 2016 | India | Asia | Indian | 41/100 | Negative | 9 | 25 | 7 | 47 | 42 | 11 | 0.7269 | 13 |
| 9 | Proestling 2012 | Austria | Europe | Caucasian | 53/220 | Positive | 23 | 27 | 3 | 125 | 87 | 8 | 0.1272 | 13 |
| 10 | Proestling 2012 | Austria | Europe | Caucasian | 143/220 | Negative | 68 | 65 | 10 | 125 | 87 | 8 | 0.1272 | 13 |
| 11 | Damin 2006 | Brazil | South America | Mixed | 48/202 | Positive | 22 | 23 | 3 | 70 | 111 | 21 | 0.0171 | 10 |
| 12 | Damin 2006 | Brazil | South America | Mixed | 70/202 | Negative | 42 | 25 | 3 | 70 | 111 | 21 | 0.0171 | 10 |
| 13 | Noma 2004 | Japan | Asia | Asian | 54/218 | Positive | 26 | 20 | 8 | 111 | 76 | 31 | 0.0041 | 14 |
| 14 | Noma 2004 | Japan | Asia | Asian | 124/218 | Negative | 59 | 44 | 21 | 111 | 76 | 31 | 0.0041 | 14 |
| **Distant metastases** | | | | | | | | | | | | | | |
| 1 | Ayoubi 2018 | Morocco | Africa | Caucasian | 8/126 | Positive | 5 | 1 | 2 | 65 | 46 | 15 | 0.1344 | 13 |
| 2 | Ayoubi 2018 | Morocco | Africa | Caucasian | 112/126 | Negative | 49 | 38 | 25 | 65 | 46 | 15 | 0.1344 | 13 |
| 3 | Yadav 2016 | India | Asia | Indian | 7/100 | Positive | 1 | 2 | 4 | 47 | 42 | 11 | 0.7269 | 13 |
| 4 | Yadav 2016 | India | Asia | Indian | 93/100 | Negative | 25 | 53 | 15 | 47 | 42 | 11 | 0.7269 | 13 |

| No. | First Author/Year | Country | Geographic region | Ethnicity | Sample size (case/control) | Source of controls | Genotypes distribution of TP53 IVS3 16bp (rs17878362) | | | | | | HWE | Quality score |
|---|---|---|---|---|---|---|---|---|---|---|---|---|---|---|
| | | | | | | | Cases | | | Controls | | | | |
| | | | | | | | CC | CG | GG | CC | CG | GG | | |
| **ER status** | | | | | | | | | | | | | | |
| 1 | Morten 2019 | Australia | Oceania | Caucasian | 648/436 | Positive | 498 | 137 | 13 | 325 | 104 | 7 | 0.6872 | 19 |
| 2 | Eskandari-Nasab 2015 | Iran | Asia | Caucasian | 180/203 | Positive | 60 | 55 | 65 | 113 | 67 | 23 | 0.0110 | 9 |
| 3 | Eskandari-Nasab 2015 | Iran | Asia | Caucasian | 120/203 | Negative | 40 | 45 | 35 | 113 | 67 | 23 | 0.0110 | 9 |
| 4 | Marouf 2014 | Morocco | Africa | Caucasian | 64/114 | Positive | 46 | 16 | 2 | 78 | 28 | 8 | 0.0240 | 11 |
| 5 | Marouf 2014 | Morocco | Africa | Caucasian | 41/114 | Negative | 27 | 12 | 2 | 78 | 28 | 8 | 0.0240 | 11 |
| 6 | Cherdyntseva 2012 | Russia | Europe | Caucasian | 141/196 | Positive | 106 | 35 | 0 | 145 | 50 | 1 | 0.1284 | 17 |
| 7 | Cherdyntseva 2012 | Russia | Europe | Caucasian | 66/196 | Negative | 53 | 13 | 0 | 145 | 50 | 1 | 0.1284 | 17 |
| 8 | Akkiprik 2009 | Turkey | Asia | Caucasian | 46/107 | Positive | 27 | 19 | | 61 | 43 | 3 | 0.1530 | 12 |
| 9 | Akkiprik 2009 | Turkey | Asia | Caucasian | 19/107 | Negative | 8 | 11 | | 61 | 43 | 3 | 0.1530 | 12 |
| 10 | Morten 2019 | Australia | Oceania | Caucasian | 656/436 | Negative | 488 | 152 | 16 | 325 | 104 | 7 | 0.6872 | 19 |
| **PR status** | | | | | | | | | | | | | | |
| 1 | Eskandari-Nasab 2015 | Iran | Asia | Caucasian | 176/203 | Positive | 56 | 62 | 58 | 113 | 67 | 23 | 0.0110 | 9 |
| 2 | Eskandari-Nasab 2015 | Iran | Asia | Caucasian | 124/203 | Negative | 44 | 38 | 42 | 113 | 67 | 23 | 0.0110 | 9 |
| 3 | Marouf 2014 | Morocco | Africa | Caucasian | 57/114 | Positive | 44 | 11 | 2 | 78 | 28 | 8 | 0.0240 | 11 |
| 4 | Marouf 2014 | Morocco | Africa | Caucasian | 48/114 | Negative | 29 | 17 | 2 | 78 | 28 | 8 | 0.0240 | 11 |
| 5 | Akkiprik 2009 | Turkey | Asia | Caucasian | 42/107 | Positive | 25 | 17 | | 61 | 43 | 3 | 0.1530 | 12 |
| 6 | Akkiprik 2009 | Turkey | Asia | Caucasian | 21/107 | Negative | 10 | 11 | | 61 | 43 | 3 | 0.1530 | 12 |
| 7 | Morten 2019 | Australia | Oceania | Caucasian | 656/436 | Negative | 488 | 152 | 16 | 325 | 104 | 7 | 0.6872 | 19 |
| **HER-2 status** | | | | | | | | | | | | | | |
| 1 | Eskandari-Nasab 2015 | Iran | Asia | Caucasian | 160/203 | Positive | 53 | 57 | 50 | 113 | 67 | 23 | 0.0110 | 9 |
| 2 | Eskandari-Nasab 2015 | Iran | Asia | Caucasian | 140/203 | Negative | 47 | 43 | 50 | 113 | 67 | 23 | 0.0110 | 9 |
| 3 | Morten 2019 | Australia | Oceania | Caucasian | 656/436 | Negative | 488 | 152 | 16 | 325 | 104 | 7 | 0.6872 | 19 |

Supplemental Table 7 continued

| | | | | | | | | | | | | | | |
|---|---|---|---|---|---|---|---|---|---|---|---|---|---|---|
| **Tumor stage** | | | | | | | | | | | | | | |
| 1 | Guleria 2012 | India | Asia | Indian | 7/80 | Stage I | 3 | 3 | 1 | 53 | 25 | 2 | 0.6360 | 14 |
| 2 | Guleria 2012 | India | Asia | Indian | 42/80 | Stage II | 24 | 15 | 3 | 53 | 25 | 2 | 0.6360 | 14 |
| 3 | Guleria 2012 | India | Asia | Indian | 25/80 | Stage III | 14 | 8 | 3 | 53 | 25 | 2 | 0.6360 | 14 |
| 4 | Guleria 2012 | India | Asia | Indian | 6/80 | Stage IV | 2 | 4 | 0 | 53 | 25 | 2 | 0.6360 | 14 |
| 5 | Pouladi 2014 | Iran | Asia | Caucasian | 80/170 | Stage I or II | 44 | 29 | 7 | 107 | 51 | 12 | 0.0963 | 13 |
| 6 | Pouladi 2014 | Iran | Asia | Caucasian | 90/170 | Stage III | 55 | 29 | 6 | 107 | 51 | 12 | 0.0963 | 13 |
| 7 | Eskandari-Nasab 2015 | Iran | Asia | Caucasian | 72/203 | Stage I | 17 | 26 | 29 | 113 | 67 | 23 | 0.0110 | 9 |
| 8 | Eskandari-Nasab 2015 | Iran | Asia | Caucasian | 101/203 | Stage II | 41 | 35 | 25 | 113 | 67 | 23 | 0.0110 | 9 |
| 9 | Eskandari-Nasab 2015 | Iran | Asia | Caucasian | 82/203 | Stage III | 31 | 22 | 29 | 113 | 67 | 23 | 0.0110 | 9 |
| 10 | Eskandari-Nasab 2015 | Iran | Asia | Caucasian | 46/203 | Stage IV | 11 | 17 | 18 | 113 | 67 | 23 | 0.0110 | 9 |
| **Tumour grade** | | | | | | | | | | | | | | |
| 1 | Morten 2019 | Australia | Oceania | Caucasian | 53/436 | Grade I | 44 | 9 | 0 | 325 | 104 | 7 | 0.6872 | 19 |
| 2 | Morten 2019 | Australia | Oceania | Caucasian | 256/436 | Grade II | 193 | 55 | 8 | 325 | 104 | 7 | 0.6872 | 19 |
| 3 | Morten 2019 | Australia | Oceania | Caucasian | 861/436 | Grade III | 652 | 193 | 16 | 325 | 104 | 7 | 0.6872 | 19 |
| 4 | Eskandari-Nasab 2015 | Iran | Asia | Caucasian | 65/203 | Grade I | 25 | 24 | 16 | 113 | 67 | 23 | 0.0110 | 9 |
| 5 | Eskandari-Nasab 2015 | Iran | Asia | Caucasian | 190/203 | Grade II | 56 | 62 | 72 | 113 | 67 | 23 | 0.0110 | 9 |
| 6 | Eskandari-Nasab 2015 | Iran | Asia | Caucasian | 43/203 | Grade III | 18 | 13 | 12 | 113 | 67 | 23 | 0.0110 | 9 |
| 7 | Marouf 2014 | Morocco | Africa | Caucasian | 6/114 | Grade I | 4 | 1 | 1 | 78 | 28 | 8 | 0.0240 | 11 |
| 8 | Marouf 2014 | Morocco | Africa | Caucasian | 71/114 | Grade II | 50 | 18 | 3 | 78 | 28 | 8 | 0.0240 | 11 |
| 9 | Marouf 2014 | Morocco | Africa | Caucasian | 28/114 | Grade III | 19 | 9 | 0 | 78 | 28 | 8 | 0.0240 | 11 |
| 10 | Akkiprik 2009 | Turkey | Asia | Caucasian | 23/107 | Grade I | 14 | 9 | | 61 | 43 | 3 | 0.1530 | 12 |
| 11 | Akkiprik 2009 | Turkey | Asia | Caucasian | 36/107 | Grade II | 23 | 13 | | 61 | 43 | 3 | 0.1530 | 12 |
| 12 | Akkiprik 2009 | Turkey | Asia | Caucasian | 48/107 | Grade III | 14 | 34 | | 61 | 43 | 3 | 0.1530 | 12 |
| 13 | Själander 1996 | Sweden | Europe | Caucasian | 56/689 | Grade I | 42 | 12 | 2 | 529 | 142 | 18 | 0.0276 | 16 |
| 14 | Själander 1996 | Sweden | Europe | Caucasian | 59/689 | Grade II | 47 | 11 | 1 | 529 | 142 | 18 | 0.0276 | 16 |
| 15 | Själander 1996 | Sweden | Europe | Caucasian | 54/689 | Grade III | 43 | 11 | 0 | 529 | 142 | 18 | 0.0276 | 16 |
| **Tumor size** | | | | | | | | | | | | | | |
| 1 | Eskandari-Nasab 2015 | Iran | Asia | Caucasian | 115/203 | T1 | 36 | 40 | 39 | 113 | 67 | 23 | 0.0110 | 9 |
| 2 | Eskandari-Nasab 2015 | Iran | Asia | Caucasian | 184/203 | T2 | 63 | 60 | 61 | 113 | 67 | 23 | 0.0110 | 9 |
| 3 | Eskandari-Nasab 2015 | Iran | Asia | Caucasian | 1/203 | T3 | 1 | 0 | 0 | 113 | 67 | 23 | 0.0110 | 9 |
| 4 | Marouf 2014 | Morocco | Africa | Caucasian | 14/114 | T1 | 9 | 4 | 1 | 78 | 28 | 8 | 0.0240 | 11 |
| 5 | Marouf 2014 | Morocco | Africa | Caucasian | 51/114 | T2 | 40 | 9 | 2 | 78 | 28 | 8 | 0.0240 | 11 |
| 6 | Marouf 2014 | Morocco | Africa | Caucasian | 20/114 | T3 | 14 | 6 | 0 | 78 | 28 | 8 | 0.0240 | 11 |
| **Menopausal status** | | | | | | | | | | | | | | |
| 1 | Akkiprik 2009 | Turkey | Asia | Caucasian | 23/107 | Pre | 14 | 9 | | 61 | 43 | 3 | 0.1530 | 12 |
| 2 | Akkiprik 2009 | Turkey | Asia | Caucasian | 64/107 | Post | 38 | 26 | | 61 | 43 | 3 | 0.1530 | 12 |
| 3 | Cherdyntseva 2012 | Russia | Europe | Caucasian | 109/94 | Pre | 79 | 30 | 0 | 67 | 27 | 0 | 0.1040 | 14 |
| 4 | Cherdyntseva 2012 | Russia | Europe | Caucasian | 186/100 | Post | 147 | 38 | 1 | 76 | 23 | 1 | 0.6071 | 14 |
| 5 | Marouf 2014 | Morocco | Africa | Caucasian | 76/114 | Pre | 51 | 21 | 4 | 78 | 28 | 8 | 0.0240 | 11 |
| 6 | Marouf 2014 | Morocco | Africa | Caucasian | 29/114 | Post | 22 | 7 | 0 | 78 | 28 | 8 | 0.0240 | 11 |
| **Age** | | | | | | | | | | | | | | |
| 1 | Pouladi 2014 | Iran | Asia | Caucasian | 108/170 | ≥45 years | 64 | 34 | 10 | 107 | 51 | 12 | 0.0963 | 13 |
| 2 | Pouladi 2014 | Iran | Asia | Caucasian | 105/170 | <45 years | 65 | 33 | 7 | 107 | 51 | 12 | 0.0963 | 13 |
| 3 | Marouf 2014 | Morocco | Africa | Caucasian | 46/114 | ≥40 years | 30 | 14 | 2 | 78 | 28 | 8 | 0.0240 | 11 |
| 4 | Marouf 2014 | Morocco | Africa | Caucasian | 59/114 | <40 years | 43 | 14 | 2 | 78 | 28 | 8 | 0.0240 | 11 |
| 5 | Akkiprik 2009 | Turkey | Asia | Caucasian | 66/107 | ≥51 years | 40 | 26 | | 61 | 43 | 3 | 0.1530 | 12 |
| 6 | Akkiprik 2009 | Turkey | Asia | Caucasian | 30/107 | <51 years | 18 | 12 | | 61 | 43 | 3 | 0.1530 | 12 |
| **Localization** | | | | | | | | | | | | | | |
| 1 | Pouladi 2014 | Iran | Asia | Caucasian | 102/170 | Right | 63 | 32 | 7 | 107 | 51 | 12 | 0.0963 | 13 |
| 2 | Pouladi 2014 | Iran | Asia | Caucasian | 107/170 | Left | 65 | 33 | 9 | 107 | 51 | 12 | 0.0963 | 13 |
| 3 | Pouladi 2014 | Iran | Asia | Caucasian | 4/170 | Right and left | 3 | 0 | 1 | 107 | 51 | 12 | 0.0963 | 13 |
| **Histological subtype** | | | | | | | | | | | | | | |
| 1 | Morten 2019 | Australia | Oceania | Caucasian | 656/436 | TNBC | 488 | 152 | 16 | 325 | 104 | 7 | 0.6872 | 19 |
| 2 | Eskandari-Nasab 2015 | Iran | Asia | Caucasian | 190/203 | Ductal carcinoma | 66 | 67 | 57 | 113 | 67 | 23 | 0.0110 | 9 |
| 3 | Marouf 2014 | Morocco | Africa | Caucasian | 98/114 | IDC | 67 | 27 | 4 | 78 | 28 | 8 | 0.0240 | 11 |
| 4 | Marouf 2014 | Morocco | Africa | Caucasian | 4/114 | ILC | 3 | 1 | 0 | 78 | 28 | 8 | 0.0240 | 11 |
| **Lymph node** | | | | | | | | | | | | | | |
| 1 | Eskandari-Nasab 2015 | Iran | Asia | Caucasian | 204/203 | Positive | 75 | 71 | 58 | 113 | 67 | 23 | 0.0110 | 9 |
| 2 | Eskandari-Nasab 2015 | Iran | Asia | Caucasian | 96/203 | Negative | 24 | 29 | 43 | 113 | 67 | 23 | 0.0110 | 9 |
| 3 | Pouladi 2014 | Iran | Asia | Caucasian | 108/170 | Positive | 68 | 32 | 8 | 107 | 51 | 12 | 0.0963 | 13 |
| 4 | Pouladi 2014 | Iran | Asia | Caucasian | 61/170 | Negative | 33 | 22 | 6 | 107 | 51 | 12 | 0.0963 | 13 |
| 5 | Marouf 2014 | Morocco | Africa | Caucasian | 62/114 | Positive | 46 | 14 | 2 | 78 | 28 | 8 | 0.0240 | 11 |
| 6 | Marouf 2014 | Morocco | Africa | Caucasian | 33/114 | Negative | 27 | 4 | 2 | 78 | 28 | 8 | 0.0240 | 11 |

| No. | First Author/Year | Country | Geographic region | Ethnicity | Sample size (case/control) | Source of controls | Genotypes distribution of TP53 IVS6+62A>G (rs1625895) | | | | | | HWE | Quality score |
|---|---|---|---|---|---|---|---|---|---|---|---|---|---|---|
| | | | | | | | Cases | | | Controls | | | | |
| | | | | | | | CC | CG | GG | CC | CG | GG | | |
| **ER status** | | | | | | | | | | | | | | |
| 1 | Cherdyntseva 2012 | Russia | Europe | Caucasian | 197/193 | Positive | 141 | 53 | 3 | 147 | 45 | 1 | 0.2102 | 17 |
| 2 | Cherdyntseva 2012 | Russia | Europe | Caucasian | 102/193 | Negative | 82 | 18 | 2 | 147 | 45 | 1 | 0.2102 | 17 |
| 3 | Akkiprik 2009 | Turkey | Asia | Caucasian | 46/107 | Positive | 22 | | 24 | 61 | 38 | 8 | 0.5431 | 12 |
| 4 | Akkiprik 2009 | Turkey | Asia | Caucasian | 19/107 | Negative | 10 | | 9 | 61 | 38 | 8 | 0.5431 | 12 |
| **PR status** | | | | | | | | | | | | | | |
| 1 | Akkiprik 2009 | Turkey | Asia | Caucasian | 42/107 | Positive | 20 | | 22 | 61 | 38 | 8 | 0.5431 | 12 |
| 2 | Akkiprik 2009 | Turkey | Asia | Caucasian | 21/107 | Negative | 10 | | 11 | 61 | 38 | 8 | 0.5431 | 12 |
| **Tumour grade** | | | | | | | | | | | | | | |
| 1 | Akkiprik 2009 | Turkey | Asia | Caucasian | 23/107 | Grade I | 11 | | 12 | 61 | 38 | 8 | 0.5431 | 12 |
| 2 | Akkiprik 2009 | Turkey | Asia | Caucasian | 36/107 | Grade II | 13 | | 23 | 61 | 38 | 8 | 0.5431 | 12 |
| 3 | Akkiprik 2009 | Turkey | Asia | Caucasian | 26/107 | Grade III | 15 | | 11 | 61 | 38 | 8 | 0.5431 | 12 |
| 4 | Själander 1996 | Sweden | Europe | Caucasian | 56/689 | Grade I | 41 | 14 | 1 | 525 | 146 | 18 | 0.0468 | 16 |
| 5 | Själander 1996 | Sweden | Europe | Caucasian | 59/689 | Grade II | 47 | 12 | 0 | 525 | 146 | 18 | 0.0468 | 16 |
| 6 | Själander 1996 | Sweden | Europe | Caucasian | 54/689 | Grade III | 42 | 11 | 1 | 525 | 146 | 18 | 0.0468 | 16 |
| **Menopausal status** | | | | | | | | | | | | | | |
| 1 | Singh 2008 | India | Asia | Indian | 34/69 | Pre | 25 | 7 | 2 | 52 | 15 | 2 | 0.4829 | 11 |
| 2 | Singh 2008 | India | Asia | Indian | 70/36 | Post | 55 | 13 | 2 | 22 | 13 | 1 | 0.5697 | 11 |
| 3 | Akkiprik 2009 | Turkey | Asia | Caucasian | 23/107 | Pre | 9 | | 14 | 61 | 38 | 8 | 0.5431 | 12 |
| 4 | Akkiprik 2009 | Turkey | Asia | Caucasian | 64/107 | Post | 31 | | 33 | 61 | 38 | 8 | 0.5431 | 12 |
| 5 | Cherdyntseva 2012 | Russia | Europe | Caucasian | 144/94 | Pre | 101 | 40 | 3 | 72 | 22 | 0 | 0.1988 | 15 |
| 6 | Cherdyntseva 2012 | Russia | Europe | Caucasian | 248/97 | Post | 191 | 54 | 3 | 73 | 23 | 1 | 0.5806 | 15 |
| **Age** | | | | | | | | | | | | | | |
| 1 | Akkiprik 2009 | Turkey | Asia | Caucasian | 67/107 | ≥51 years | 34 | | 33 | 61 | 38 | 8 | 0.5431 | 12 |
| 2 | Akkiprik 2009 | Turkey | Asia | Caucasian | 31/107 | <51 years | 14 | | 17 | 61 | 38 | 8 | 0.5431 | 12 |

Supplemental Table 7 continued

| | | | | | | | | | | | | | | |
|---|---|---|---|---|---|---|---|---|---|---|---|---|---|---|
| 3 | Sprague 2007 | USA | North America | Caucasian | 991/854 | ≥50 years | 745 | 223 | 23 | 645 | 190 | 19 | 0.2632 | 19 |
| 4 | Sprague 2007 | USA | North America | Caucasian | 482/421 | <50 years | 387 | 86 | 9 | 295 | 115 | 11 | 0.9585 | 19 |
