# Supplementary material for: Association Between the TP53 Polymorphisms and Breast Cancer Risk: An Updated Meta-Analysis
Source: Front Genet. 2022 Apr 27;13:807466. doi: 10.3389/fgene.2022.807466 (PMC9091657; doi:10.3389/fgene.2022.807466)
Supplement: Supplementary file 2 [file DataSheet2.PDF]

Supplemental Table 2. Included studies of TP53 codon 72 (rs1042522) polymorphism in BC risk within the meta-analyses (A, Asian; I: Indian; Af, African; C, Caucasian; ME, Middle East; H, Hispanic; M, mixed; U, unidentified).

No.	First Author/Year	Country	All studies													
			**This study, 2021**	Diakite 2020	Gonçalves 2014	Hou 2013	Dahabreh 2013	Cheng 2012	Ma 2011	He 2011	Francisco 2011	Zhang 2010	Hu 2010(2)	Zhuo 2009	Suspitsin 2003	Dunning 1999
1	Kawajiri 1993	Japan	A	–	–	A	–	–	–	A	A	U	A	–	Japan	Japanese
2	Själander 1996	Sweden	C	–	–	C	–	C	C	C	C	U	European	Sweden	Sweden	White Swedish
3	Weston 1997a	USA	C	–	–	C	–	C	C	C	C	U	European	USA	USA (Caucasians)	–
4	Weston 1997b	USA	H	–	–	U	–	H	H	H	M	–			–	–
5	Weston 1997c	USA	Af	–	–	U	–	Af	Af	Af	Af	–			–	–
6	Helland 1998	USA	M	–	–	U	–	–	–	U	–	–	–	–	Norway	–
7	Wang-Gohrke 1998	Germany	Exclude	–	–	C	–	–	–	C	C	U	–	–	Germany	U
8	Khaliq 2000	Pakistan	I	–	–	A	–	–	–	A	I	U	–	–	–	–
9	Papadakis 2000	Greece	C(not in HWE)	–	–	C	–	–	–	C(not in HWE)	C	U (not in HWE)	European	Excluded (not in HWE)	–	–
10	Li 2002	China	A	A	Asia	A	–	A	A	A	–	U	–	China	–	–
11	Wang-Gohrke 2002	Germany	Exclude	C	Europe	C	–	C	C	C	C	U	European	Germany	Germany	–
12	Buyru 2003	Turkey	Exclude	C	Europe	C	U	C	–	C	M	U	–	Turkey	–	–
13	Huang 2003	Japan	A	–	Asia	A	U	A	A	A	A	U	A	China	–	–
14	Katiyar 2003	India	I	A	Asia	A	U	A	A	A	I	–	A	India	–	–
15	Mabrouk 2003	Tunisia	C	–	Africa	U	–	Af	Af	Af	M	U	European	Tunisia	–	–
16	Suspitsin 2003	Russia	C	–	Europe	C	–	–	–	C	C	U	European	–	Russia	–
17	Mahasneh 2004	Jordan	C(not in HWE)	–	–	A	–	–	–	A	–	U	–	–	–	–
18	Menzel 2004a	Czech	C	C	Europe	C	–	–	–	C	–	–	European	–	–	–
19	Menzel 2004b	Czech	C				–	–	–		–	–	European	–	–	–
20	Noma 2004	Japan	A(not in HWE)	–	Asia	A	–	–	–	A	A	U (not in HWE)	A	Excluded (not in HWE)	–	–
21	Kalemi 2005	UK	C	–	Europe	C	U	C	C	C	C	U	European	UK	–	–
22	Ohayon 2005	Israel	C(not in HWE)	–	Europe	C	–	–	–	C	Jew Askhenazi	U	European	Excluded (not in HWE)	–	–
23	Siddique 2005	China	Exclude	–	Asia	A	–	–	–	A	–	U	A	–	–	–
24	Tommiska 2005	Finland	C	–	Europe	C	–	C	C	C	C	U	European	Sweden	–	–
25	BCAC 2006 Copenhagen	Danish	C	–	–	–	U	–	–	–	C	–	–	–	–	–
26	BCAC 2006 GENICA	Germany	C	–	–	–		–	–	–	C	–	–	–	–	–
27	BCAC 2006 GESBC	Germany	C	–	–	–		–	–	–	–	–	–	–	–	–
28	BCAC 2006 HBCS	Finland	Exclude	–	–	–		–	–	–	–	–	–	–	–	–
29	BCAC 2006 IARC-Thai	Thailand	A	–	–	–		–	–	–	M	–	–	–	–	–
30	BCAC 2006 LSHTM	UK	C	–	–	–		–	–	–	–	–	–	–	–	–
31	BCAC 2006 PBSC	Poland	Exclude	–	–	–		–	–	–	C	–	–	–	–	–
32	BCAC 2006 SEARCH	UK	Exclude	–	–	–		–	–	–	–	–	–	–	–	–
33	BCAC 2006 USRTS	USA	M	–	–	–		–	–	–	M	–	–	–	–	–
34	Damin 2006	Brazil	M(not in HWE)	–	America	U	U	–	M	M	M	U	–	Excluded (not in HWE)	–	–
35	Ma 2006	China	A	A	Asia	A	–	–	–	A	A	U	A	–	–	–
36	Baynes 2007	UK	C	–	Europe	C	U	C	C	C	C	U	European	UK	–	–
37	Buyru 2007	Turkey	C	–	Europe	–	U	–	C	–	M	U	European	–	–	–
38	Cox 2007	USA	M	C	America	U	U	C	–	M	M	U	M	–	–	–
39	Franeková 2007	Slovakia	C	–	–	C	U	–	–	C	–	U	–	–	–	–
40	Garcia-Closas 2007	Norway and Poland	C	–	America	C	U	–	–	C	C	U	European	–	–	–
41	Gochhait 2007	India	I	–	Asia	A	–	A	A	A	I	U	A	India	–	–
42	Johnson 2007	UK	C	–	Europe	C	–	–	–	C	C	U	European	–	–	–
43	Khadang 2007	Iran	C	–	Asia	A	–	A	A	A	ME	U	A	Iran	–	–
44	Pharoah 2007	Mixed (Nine European countries)	Exclude	–	–	–	–	–	–	Exclude	–	U	–	–	–	–
45	Samson 2007	India	Exclude	–	–	–	–	–	–	Exclude	I	U	–	–	–	–
46	Schmidt 2007a	Finland	C	–	Europe	C	–	C	C	C	C	U	European	UK	–	–
47	Schmidt 2007b	Germany	C	–			–	C				–	European	–	–	–
48	Schmidt 2007c	Netherlands	C	–			–	C				–	European	–	–	–
49	Schmidt 2007d	UK	C	–			–	C				–	European	–	–	–
50	Schmidt 2007e	UK	C	–			–	C				–	European	–	–	–
51	Sprague 2007	USA	C	C	America	C	–	C	NR	C	M	U	European	USA	–	–
52	Zhang 2007	China	A	A	–	A	–	A	A	A	–	–	–	China	–	–
53	Cavallone 2008	France	C	–	Europe	C	U	–	–	C	C	U	European	–	–	–
54	Costa 2008a	Portugal	C(not in HWE)	C	Europe	C	U	–	–	C	C	U	European	Excluded (not in HWE)	–	–
55	Costa 2008b	Portugal	C					–	–			–			–	–
56	De 2008	Italy	C	–	–	C	U	–	–	C	C	U	–	–	–	–
57	Gaudet 2008	USA	M	–	America	U	U	–	–	M	M	U	M	–	–	–
58	Lum 2008	China	A	–	Asia	A	–	A	–	A	A	U	A	–	–	–
59	Nordgard 2008	Norway	C(not in HWE)	–	–	C	–	–	–	C	–	U	–	–	–	–
60	Rajkumar 2008	India	I(not in HWE)	–	Asia	A	–	–	–	A	–	–	A	–	–	–
61	Singh 2008	India	I(not in HWE)	–	Asia	A	–	–	–	A	I	U (not in HWE)	A	–	–	–
62	Akkiprik 2009	Turkey	C	C	Europe	C	U	C	C	C	–	–	–	Turkey	–	–
63	Aoki 2009	Brazil	M(not in HWE)	–	America	U	U	M	M	M	–	–	–	–	–	–
64	Denisov 2009	Russia	Exclude	C	Europe	C	U	C	–	C	–	–	–	–	–	–
65	Henríquez-Hernández 2009	Spain	C	–	Europe	C	U	C	C	C	–	–	–	–	–	–
66	Hrstka 2009	Czech	C(not in HWE)	–	–	C	U	–	–	C(not in HWE)	–	–	–	–	–	–
67	Kazemi 2009	Iran	C(not in HWE)	–	Asia	A	–	–	A	A	–	–	–	–	–	–
68	Lång 2009	Sweden	C	–	–	C	–	C	–	C	–	–	–	–	–	–
69	Sinilnikova 2009	12 Countries	C	–	Europe	–	–	–	–	–	–	–	–	–	–	–
70	Song 2009	China	A	A	–	A	–	–	–	A	–	–	–	–	–	–
71	Bisof 2010	Croatia	C	–	Europe	C	U	C	–	C	–	–	–	–	–	–
72	Ebner 2010	Germany	C	C	–	C	U	C	–	C	–	–	–	–	–	–
73	Jakubowska 2010	Poland	C	–	–	–	–	–	–	–	–	–	–	–	–	–
74	Kara 2010	Turkey	C	–	Europe	A	U	C	–	C	–	–	–	–	–	–
75	Nan 2010	China	A	–	–	–	–	–	–	–	–	–	–	–	–	–
76	Syeed 2010	Kashmir	Exclude	–	–	–	–	–	–	–	–	–	–	–	–	–
77	Trifa 2010	Tunisia	C	–	Africa	–	–	Af	–	–	–	–	–	–	–	–
78	Alawadi 2011	Arabia	C(not in HWE)	–	Asia	C	U	–	–	–	–	–	–	–	–	–
79	Koh 2011	Singapore	A	–	–	A	–	A	–	–	–	–	–	–	–	–
80	Leu 2011	China	A(not in HWE)	–	–	A	–	–	–	–	–	–	–	–	–	–
81	Yoshimoto 2011	Japan	A	–	–	–	–	–	–	–	–	–	–	–	–	–
82	Alshatwi 2012	Arabia	C	A	–	C	–	C	–	–	–	–	–	–	–	–
83	Cherdyntseva 2012	Russia	C	C	–	–	–	–	–	–	–	–	–	–	–	–
84	Guleria 2012	India	I	–	–	–	–	–	–	–	–	–	–	–	–	–
85	Proestling 2012	Austria	C	–	–	–	–	–	–	–	–	–	–	–	–	–
86	Lajin 2013	Syria	C	–	–	–	–	–	–	–	–	–	–	–	–	–
87	Liu 2013	China	A	–	–	–	–	–	–	–	–	–	–	–	–	–
88	Rodrigues 2013	Spain	C	–	–	–	–	–	–	–	–	–	–	–	–	–
89	Wang 2013	China	A	–	–	–	–	–	–	–	–	–	–	–	–	–
90	Krivokuca 2014	Serbia	C	C	–	–	–	–	–	–	–	–	–	–	–	–
91	Saadatian 2014	Iran	C(not in HWE)	–	–	–	–	–	–	–	–	–	–	–	–	–
92	Sharma 2014	India	I	A	–	–	–	–	–	–	–	–	–	–	–	–
93	Arfaoui 2015	Tunisia	C	–	–	–	–	–	–	–	–	–	–	–	–	–
94	Devi 2015	India	I(not in HWE)	–	–	–	–	–	–	–	–	–	–	–	–	–
95	Gohari-Lasaki 2015	Iran	C	–	–	–	–	–	–	–	–	–	–	–	–	–
96	Vymetalkova 2015	Czech	C	–	–	–	–	–	–	–	–	–	–	–	–	–
97	Almeida 2016	Brazil	M	–	–	–	–	–	–	–	–	–	–	–	–	–
98	Golmohammadi 2016	Iran	C(not in HWE)	–	–	–	–	–	–	–	–	–	–	–	–	–
99	Shabnaz 2016	Bangladesh	I	–	–	–	–	–	–	–	–	–	–	–	–	–
100	Yadav 2016	India	I	–	–	–	–	–	–	–	–	–	–	–	–	–
101	Hossain 2017	Bangladesh	I	A	–	–	–	–	–	–	–	–	–	–	–	–
102	Isakova 2017	Kyrgyzstan	Exclude	A	–	–	–	–	–	–	–	–	–	–	–	–
103	Akhter 2018	India	Exclude	–	–	–	–	–	–	–	–	–	–	–	–	–
104	Ayoubi 2018	Morocco	C	Af	–	–	–	–	–	–	–	–	–	–	–	–
105	Chen 2018	China	A	–	–	–	–	–	–	–	–	–	–	–	–	–
106	Habyarimana 2018	Rwanda	Af(not in HWE)	–	–	–	–	–	–	–	–	–	–	–	–	–
107	Hao 2018	China	A	–	–	–	–	–	–	–	–	–	–	–	–	–
108	Afzaljavan 2020	Iran	C(not in HWE)	–	–	–	–	–	–	–	–	–	–	–	–	–
109	Icen-Taskin 2020	Turkey	C	–	–	–	–	–	–	–	–	–	–	–	–	–
110	Isakova 2020	Kyrgyzstan	A	–	–	–	–	–	–	–	–	–	–	–	–	–
111	Pouladi 2020	Iran	C	–	–	–	–	–	–	–	–	–	–	–	–	–
112	Akhter 2021	India	I(not in HWE)	–	–	–	–	–	–	–	–	–	–	–	–	–

Dunning et al. 1999, Suspitsin et al. 2003, Gonçalves et al. 2014 did not report HWE of collected studys. Zhuo et al. 2009, Hu et al. 2010(1), Hu et al. 2010(2), Zhang et al. 2010, Francisco et al. 2011, He et al. 2011, Ma et al. 2011, Cheng et al. 2012, Dahabreh et al. 2013, Hou et al. 2013, Sagne et al. 2013, Wu et al. 2013, Diakite et al. 2020 reported HWE of included studies. The studys not in HWE were excluded in Meta-analysis by Zhuo et al. 2009, He et al. 2011, Ma et al. 2011, Cheng et al. 2012.

Dunning et al. 1999, Suspitsin et al. 2003, Zhuo et al. 2009, Hu et al. 2010(1), Hu et al. 2010(2), Zhang et al. 2010, Ma et al. 2011, Sagne et al. 2013, Gonçalves et al. 2014, Diakite et al. 2020 did not check the repeatability of data. He et al. 2011, Cheng et al. 2012, Dahabreh et al. 2013, Hou et al. 2013, Wu et al. 2013 had checked the repeatability of data.

Francisco et al. 2011, Dahabreh et al. 2013 established a clear epidemiological design criteria and re-conducted meta-analyses of the data from the original studies that met the criteria, then draw conclusions on this basis.
