# Supplementary material for: Association Between the TP53 Polymorphisms and Breast Cancer Risk: An Updated Meta-Analysis
Source: Front Genet. 2022 Apr 27;13:807466. doi: 10.3389/fgene.2022.807466 (PMC9091657; doi:10.3389/fgene.2022.807466)
Supplement: Supplementary file 3 [file DataSheet4.PDF]

Supplemental Table 4. Included studies of TP53 IVS6+62A>G (rs1625895) polymorphism in BC risk within the meta-analyses (A, Asian; I: Indian; Af, African; C, Caucasian; ME, Middle East; H, Hispanic; M, mixed; U, unidentified).

No	First Author/Year	Country	All studies			
			**This study, 2021**	He 2011	Hu 2010(2)	Dunning 1999
1	Peller 1995	Israel	C	–	–	U
2	Själander 1996	Sweden	C(not in HWE)	C	European	White Swedish
3	Weston 1997	USA	C	C	European	–
4	Weston 1997	USA	H	H		–
5	Weston 1997	USA	Af	Af		–
6	Mavridou 1998	UK	C	–	–	U
7	Wang-Gohrke 1998	Germany	Exclude	–	–	U
8	Khaliq 2000	Pakistan	I	–	–	–
9	Wang-Gohrke 2002	Germany	C	C	European	–
10	Suspitsin 2003	Russia	C	C	European	–
11	Wirtenberger 2006	Germany	C	–	–	–
12	Baynes 2007	UK	C	C	European	–
13	Buyru 2007	Turkey	C	C	European	–
14	Garcia-Closas 2007	Norway and Poland	C(not in HWE)	C	European	–
15	Pharoah 2007	Mixed (Nine European countries)	Exclude	–	–	–
16	Sprague 2007	USA	C(not in HWE)	C	European	–
17	Zhang 2007	China	A(not in HWE)	–	–	–
18	Gaudet 2008	USA	M	M	M	–
19	Singh 2008	India	I	A	A	–
20	Akkiprik 2009	Turkey	C	C	–	–
21	Hrstka 2009	Czech	C	C	–	–
22	Jakubowska 2010	Poland	C	–	–	–
23	Cherdyntseva 2012	Russia	C	–	–	–
24	Lajin 2013	Syria	C	–	–	–
25	Rodrigues 2013	Spain	C	–	–	–
26	Sharma 2014	India	I	–	–	–
27	Assad 2019	Iran	C	–	–	–
