# Supplementary material for: Association Between the TP53 Polymorphisms and Breast Cancer Risk: An Updated Meta-Analysis
Source: Front Genet. 2022 Apr 27;13:807466. doi: 10.3389/fgene.2022.807466 (PMC9091657; doi:10.3389/fgene.2022.807466)
Supplement: Supplementary file 4 [file DataSheet6.PDF]

Supplemental Table 6. Characteristics of the included studies of *TP53* polymorphism in BC risk within the present meta-analyses

No.	First Author/Year	Country	Geographic region	Ethnicity	Sample size (case/control)	Source of controls	Source of cases	Source of genotyping material of case	Type of control	Matching	Adjustments	Genotypes distribution of codon 72						HWE	Quality score	Adjusted OR and 95% CI			
												Cases			Controls					CG vs CC	GG vs CC	GG + CG vs CC	GG vs CC + CG
												CC	CG	GG	CC	CG	GG						
1	Kawajiri 1993	Japan	Asia	Asian	93/347	PB	PB	Peripheral Lymphocytes	Healthy controls	NR	NR	37	51	5	144	165	38	0.3625	14	–	–	–	–
2	Själander 1996	Sweden	Europe	Caucasian	212/689	PB	PB	Whole blood or washed and Homogenized placental tissue	NR	Age and Sex	NR	95	93	24	375	253	61	0.0550	18	–	–	–	–
3	Weston 1997a	USA	North America	Caucasian	65/117	PB	PB	Blood samples	Non-cancer controls	Age, Race and Diagnosis	NR	6	27	32	3	42	72	0.2755	15	–	–	–	–
4	Weston 1997b	USA	North America	Hispanic	18/38	PB	PB	Blood samples	Non-cancer controls	Age, Race and Diagnosis	NR	3	8	7	10	16	12	0.3376	15	–	–	–	–
5	Weston 1997c	USA	North America	African	16/30	PB	PB	Blood samples	Non-cancer controls	Age, Race and Diagnosis	NR	6	9	1	12	14	4	0.9791	15	–	–	–	–
6	Helland 1998	USA	North America	Mixed	109/225	PB	PB	NR	NR	Age	NR	63	40	6	122	90	13	0.4966	13	–	–	–	–
7	Khaliq 2000	Pakistan	Asia	Indian	41/689	PB	NR	Blood samples	NR	NR	NR	10	18	13	191	321	177	0.0750	12	–	–	–	–
8	Papadakis 2000	Greece	Europe	Caucasian	56/59	NR	NR	Tumor tissue	Healthy controls	NR	NR	34	10	12	12	41	6	0.0019	2	–	–	–	–
9	Li 2002	China	Asia	Asian	28/50	BV	HB	Tumor tissue	Healthy controls	Age and Study region	NR	11	11	6	10	26	14	0.7412	11	–	–	–	–
10	Huang 2003	Japan	Asia	Asian	200/282	HB	HB	Blood samples	Healthy controls	NR	Age	64	100	36	114	138	30	0.2139	13	1.30 (0.87, 1.95)	2.10 (1.18, 3.73)	–	–
11	Katiyar 2003	India	Asia	Indian	77/41	HB	HB	Tumor tissue	Healthy controls	Age and without a family history of cancer	NR	20	51	6	9	24	8	0.2723	12	–	–	–	–
12	Mabrouk 2003	Tunisia	Africa	Caucasian	30/49	NR	NR	Blood samples	Healthy controls	NR	NR	18	9	3	19	26	4	0.2314	6	–	–	–	–
13	Suspitsin 2003	Russia	Europe	Caucasian	529/393	BV	HB	Peripheral leukocytes	Healthy controls	NR	NR	284	203	42	207	159	27	0.6347	14	–	–	–	–
14	Mahasneh 2004	Jordan	Asia	Caucasian	43/136	HB	HB	Blood samples	Healthy controls	NR	NR	16	19	8	56	51	29	0.0106	7	–	–	–	–
15	Menzel 2004a	Czech	Europe	Caucasian	207/325	BV	HB	NR	Healthy controls	NR	NR	109	79	19	191	112	22	0.3185	13	–	–	–	–
16	Menzel 2004b	Czech	Europe	Caucasian	95/150	HB	HB	NR	Non-cancer controls	Age	NR	49	35	11	84	58	8	0.6206	13	–	–	–	–
17	Noma 2004	Japan	Asia	Asian	191/218	PB	HB	Blood mononucleated [illegible]	Non-cancer controls	NR	Age, Family history, BMI and Age at first live birth	93	69	29	111	76	31	0.0041	14	1.06 (0.69, 1.65)	1.21 (0.67, 2.20)	–	–
18	Kalemi 2005	UK	Europe	Caucasian	42/51	NR	NR	Tumor tissue	Healthy controls	NR	NR	26	13	3	10	32	9	0.0682	5	–	–	–	–
19	Ohayon 2005	Israel	Asia	Caucasian	132/167	HB	FB	Blood samples	Non-cancer controls	NR	NR	89	40	3	54	94	19	0.0218	9	–	–	–	–
20	Tommiska 2005	Finland	Europe	Caucasian	1551/733	PB	HB	Tumor tissue	Healthy controls	NR	NR	825	617	109	403	278	52	0.6689	15	–	–	–	–
21	BCAC 2006 Copenhagen	Danish	Europe	Caucasian	1155/2308	PB	HB	NR	Non-cancer controls	Age	NR	665	404	86	1247	902	159	0.8120	17	–	–	–	–
22	BCAC 2006 GENICA	Germany	Europe	Caucasian	608/645	PB	HB	NR	Non-cancer controls	Age	BMI(categories <22.5, 22.5)<25, 25)<30, 30 kg/m2), number of mammograms until 2 years prior to interview (continuous), years of OC use (continuous), parity (nulliparous, 1–2 children, 3 children), lifetime cumulative months of breastfeeding (continuous), age at menarche (continuous), age at first full-term pregnancy (nulliparous, <25, 25)<30, 30 years) and age at menopause (pre-menopausal, <45, 45)<50, 50)<55, 55 years)	334	232	42	362	243	40	0.9269	18	–	–	–	–
23	BCAC 2006	Germany	Europe	Caucasian	552/543	PB	PB	Blood samples	Non-cancer	Age and Study region	Variables did not change the estimates	282	221	49	300	203	40	0.4862	20	–	–	–	–
24	BCAC 2006 IARC-Thai	Thailand	Asia	Asian	468/384	PB	HB	NR	Non-cancer controls	Age	Age and Area	134	233	101	97	207	80	0.1159	15	–	–	–	–
25	BCAC2006 LSHTM	UK	Europe	Caucasian	584/598	PB	HB	NR	Non-cancer controls	NR	NR	322	225	37	305	247	46	0.6807	17	–	–	–	–
26	BCAC 2006	USA	North America	Mixed	725/1050	HB	HB	Peripheral	Non-cancer	Age	NR	398	276	51	552	429	69	0.2375	20	–	–	–	–
27	Damin 2006	Brazil	South America	Mixed	118/202	BV	NR	Blood samples	Non-cancer	Ethnicity and	NR	64	48	6	70	111	21	0.0171	10	–	–	–	–
28	Ma 2006	China	Asia	Asian	404/472	PB	HB	Leukocyte Pellets	Non-cancer controls	Age and Residential area	Age, BMI, Family history of cancer, Age at menarche and Menopausal status	149	178	77	150	222	100	0.2906	21	0.79 (0.58, 1.07)	0.77 (0.53, 1.12)	0.78 (0.59, 1.04)	–
29	Baynes 2007	UK	Europe	Caucasian	2023/2197	PB	PB	Blood samples	Non-cancer controls	U	NR	1107	768	148	1177	854	166	0.5201	18	–	–	–	–
30	Buyru 2007	Turkey	Asia	Caucasian	115/63	NR	NR	Blood samples	Healthy controls	Age	NR	64	39	12	26	28	9	0.7430	8	–	–	–	–
31	Cox 2007	USA	North America	Mixed	1477/2224	PB	PB	Blood samples	Non-cancer controls	Age, Menopausal status, Recent postmenopausal hormone (PMH) use, and time and Date of blood draw	Age, Fasting status, Time and date at blood draw, Age at menarche (<12 years, 12 years, 13 years, >13 years), age at first birth (AFB) and Parity (nulliparous, one to two children and AFB £24 years, one to two children and AFB >24 years, >two children and AFB £24 years, >two children and AFB >24 years), Family history of breast cancer (yes/no), Menopausal status at diagnosis (yes, no, unknown), Personal history of benign breast disease (yes/no)	804	569	104	1255	838	131	0.5677	19	1.06 (0.92, 1.22)	1.26 (0.96, 1.67)	–	–
32	Franeková 2007	Slovakia	Europe	Caucasian	91/156	HB	HB	Blood samples	Healthy controls	NR	NR	49	34	8	92	55	9	0.8372	13	–	–	–	–
33	Garcia-Closas 2007	Norway and Poland	Europe	Caucasian	2585/3251	HB	HB	Blood samples	Non-cancer controls	Age	Age and Study population	1368	1021	196	1774	1249	228	0.6865	18	1.07 (0.97, 1.21)	1.13 (0.92, 1.40)	–	–
34	Gochhait 2007	India	Asia	Indian	243/333	NR	NR	Blood samples	Healthy controls	Ethnically and Geographically matched	Age	86	109	48	76	160	97	0.5206	9	–	0.43 (0.28, 0.71)	–	–
35	Johnson 2007	UK	Europe	Caucasian	472/2462	PB	PB	Blood samples	Non-cancer controls	NR	NR	257	185	30	1354	925	183	0.1517	17	–	–	–	–
36	Khadang 2007	Iran	Asia	Caucasian	221/205	NR	HB	Blood samples	Healthy controls	NR	NR	83	109	29	75	90	40	0.1711	9	–	–	–	–
37	Schmidt 2007a	Finland	Europe	Caucasian	580/365	BV	PB	NR	Non-cancer controls	NR	NR	294	235	51	198	141	26	0.8957	13	–	–	–	–
38	Schmidt 2007b	Germany	Europe	Caucasian	1043/506	BV	PB	Blood samples	Non-cancer controls	NR	NR	565	401	77	250	217	39	0.3896	16	–	–	–	–
39	Schmidt 2007c	Netherlands	Europe	Caucasian	1247/263	BV	PB	Normal tissue	Non-cancer controls	NR	NR	668	477	102	141	109	13	0.1622	16	–	–	–	–
40	Schmidt 2007d	UK	Europe	Caucasian	4958/5130	PB	PB	Blood samples	Non-cancer controls	NR	NR	2687	1915	356	2769	1973	388	0.1603	17	–	–	–	–
41	Schmidt 2007e	UK	Europe	Caucasian	517/585	PB	PB	Blood samples	Non-cancer controls	NR	NR	285	200	32	303	237	45	0.8863	17	–	–	–	–
42	Sprague 2007	USA	North America	Caucasian	1653/1854	PB	PB	Buccal sample	Non-cancer controls	Age	NR	909	644	100	1021	704	129	0.6108	19	–	–	–	–
43	Zhang 2007	China	Asia	Asian	83/268	PB	PB	Blood samples	Non-cancer controls	Age	Age, Marriage status, Culture degree and occupation	21	45	17	74	139	55	0.4860	20	0.86 (0.46, 1.60)	0.74 (0.33, 1.64)	–	–
44	Cavallone 2008	France	Europe	Caucasian	157/112	PB	FB	Blood samples	Non-cancer controls	NR	NR	80	67	10	57	46	9	0.9473	14	–	–	–	–
45	Costa 2008a	Portugal	Europe	Caucasian	73/434	BV	FB	Blood samples	Healthy controls	Age	Age	39	25	9	256	142	36	0.0129	16	1.19 (0.68, 2.08)	1.58 (0.68, 3.67)	–	–
46	Costa 2008b	Portugal	Europe	Caucasian	175/212	BV	HB	Blood samples	Healthy controls	Age	Age	98	61	16	124	70	18	0.0819	17	1.26 (0.79, 2.02)	1.35 (0.63, 2.88)	–	–
47	De 2008	Italy	Europe	Caucasian	350/352	BV	FB	NR	Healthy controls	NR	Age	185	150	15	207	131	14	0.2279	12	1.30 (0.91, 1.85)	1.11 (0.47, 2.64)	–	–
48	Gaudet 2008	USA	North America	Mixed	578/390	PB	PB	Blood samples	Non-cancer controls	Age	Age	288	244	46	218	138	34	0.0766	21	1.32 (1.00, 1.74)	1.05 (0.65, 1.70)	–	–
49	Lum 2008	China	Asia	Asian	357/80	BV	NR	Blood samples	Healthy controls	Study region	NR	96	182	79	29	38	13	0.9258	11	–	–	–	–
50	Nordgard 2008	Norway	Europe	Caucasian	109/121	NR	NR	Blood samples	Healthy controls	NR	NR	73	34	14	46	58	5	0.0124	9	–	–	–	–
51	Rajkumar 2008	India	Asia	Indian	250/500	NR	NR	Blood samples	Healthy controls	Age	Religion, Age at menarche, Age at first child birth, Menopausal status and Consanguineous marriage	66	125	59	135	224	141	0.0202	11	–	0.81 (0.51, 1.28)	–	–
52	Singh 2008	India	Asia	Indian	104/105	HB	HB	Blood samples	Healthy controls	Study region	NR	46	45	13	28	65	12	0.0061	9	–	–	–	–

Supplemental Table 6 continued

53	Akkiprik 2009	Turkey	Asia	Caucasian	95/107	NR	HB	Tumor tissue	Healthy controls	Age	NR	25	50	20	46	49	12	0.8462	12	–	–	–	–
54	Aoki 2009	Brazil	South America	Mixed	72/90	NR	PB	Blood samples	Healthy controls	NR	NR	40	29	3	30	53	7	0.0136	7	–	–	–	–
55	Henríquez-Hernández	Spain	Europe	Caucasian	116/246	PB	HB	Blood samples	Healthy controls	Age	NR	66	44	6	145	81	20	0.0783	16	–	–	–	–
56	Hrstka 2009	Czech	Europe	Caucasian	117/108	BV	HB	Tumor tissue	Healthy controls	NR	NR	62	15	40	55	8	45	0.0001	11	–	–	–	–
57	Kazemi 2009	Iran	Asia	Caucasian	42/57	NR	HB	Tumor tissue	Non-cancer controls	Age and Ethnicity	NR	6	30	6	12	45	0	0.0001	9	–	–	–	–
58	Lång 2009	Sweden	Europe	Caucasian	116/142	BV	HB	Normal lymph node tissues	Healthy controls	NR	NR	65	45	6	79	58	5	0.1478	13	–	–	–	–
59	Sinilnikova 2009	12 Countries	Europe	Caucasian	3959/3052	PB	PB	Blood samples	Non-cancer controls	NR	NR	2164	1508	287	1660	1178	214	0.7991	18	–	–	–	–
60	Song 2009	China	Asia	Asian	1110/1097	PB	PB	Blood samples	Non-cancer controls	Age	Age, Duration of breast feeding, Menopause, Oral contraception, Smoking status, Benign breast disease, and Family history of cancer	341	547	222	355	514	228	0.0966	22	1.12 (0.91, 1.38)	1.03 (0.80, 1.34)	–	–
61	Bisof 2010	Croatia	Europe	Caucasian	95/108	BV	HB	Tumor tissue	Healthy controls	Age and Place of birth	NR	61	23	11	61	42	5	0.5074	14	–	–	–	–
62	Ebner 2010	Germany	Europe	Caucasian	263/254	HB	HB	Blood samples	Non-cancer controls	Age, Genetic origin, Status of menopause, and Substitution with hormones or Antagonists	NR	138	108	17	137	103	14	0.3432	16	–	–	–	–
63	Jakubowska 2010	Poland	Europe	Caucasian	318/290	PB	PB	Blood samples	Non-cancer controls	NR	Age at first live birth, Parity, lifetime cumulative months of breastfeeding, Age at menarche, Oral contraceptive use, Smoking, Body mass index (at age of breast cancer diagnosis for cases and at time of interview for controls), Year of birth and BRCA1 mutation	152	142	24	144	113	33	0.1388	18	1.56 (1.04, 2.34)	0.67 (0.34, 1.35)	–	–
64	Kara 2010	Turkey	Asia	Caucasian	203/169	PB	HB	Blood samples	Healthy controls	NR	NR	105	84	14	72	80	17	0.4439	14	–	–	–	–
65	Nan 2010	China	Asia	Asian	90/94	HB	HB	Tumor tissue	Healthy controls	NR	NR	30	28	32	24	42	28	0.3098	10	–	–	–	–
66	Trifa 2010	Tunisia	Africa	Caucasian	109/132	BV	HB	Blood samples	Healthy controls	NR	NR	40	49	20	44	64	24	0.9312	12	–	–	–	–
67	Alawadi 2011	Arabia	Asia	Caucasian	288/188	PB	HB	Blood samples	Healthy controls	NR	NR	81	200	7	50	112	26	0.0038	10	–	–	–	–
68	Koh 2011	Singapore	Asia	Asian	372/643	PB	PB	Blood or Buccal cells	Non-cancer controls	NR	Age at menopause, Age at recruitment, Dialect group, Level of education, Body mass index, Age at first live birth and Soy isoflavone intake	102	197	73	179	319	145	0.8992	19	–	0.87 (0.60, 1.27)	–	0.84 (0.61, 1.16)
69	Leu 2011	China	Asia	Asian	239/321	HB	HB	Blood samples	Healthy controls	NR	Age, Gender, High calorie intake, Vegetarian, and Cardiovascular diseases	71	90	78	104	129	88	0.0005	11	1.03 (0.64, 1.68)	1.1 (0.66, 1.81)	1.06 (0.69, 1.64)	–
70	Yoshimoto 2011	Japan	Asia	Asian	761/258	HB	HB	Blood samples	Non-cancer controls	NR	NR	304	356	101	111	106	41	0.0695	14	–	–	–	–
71	Alshatwi 2012	Arabia	Asia	Caucasian	100/100	BV	HB	Blood samples	Healthy controls	Age	NR	22	52	26	32	51	17	0.6637	15	–	–	–	–
72	Cherdyntseva 2012	Russia	Europe	Caucasian	388/275	BV	HB	Blood samples	Non-cancer controls	Age and Ethnicity	NR	184	162	42	148	100	27	0.1037	17	–	–	–	–
73	Guleria 2012	India	Asia	Indian	80/80	NR	HB	Blood samples	Healthy controls	Age and Gender	NR	11	47	22	27	32	21	0.0804	14	–	–	–	–
74	Proestling 2012	Austria	Europe	Caucasian	267/220	HB	HB	Blood samples	Non-cancer controls	NR	Age and Menopausal status	125	123	19	125	87	8	0.1272	13	1.53 (0.91, 2.58)	3.06 (0.74, 12.67)	1.63 (0.98, 2.71)	2.55 (0.63, 10.37)
75	Lajin 2013	Syria	Asia	Caucasian	122/139	PB	HB	Blood samples	Non-cancer controls	NR	NR	41	68	13	60	55	24	0.0736	14	–	–	–	–
76	Liu 2013	China	Asia	Asian	1100/1400	HB	HB	NR	Non-cancer controls	Age	Age and Family history of breast cancer	296	566	238	489	674	237	0.8545	17	1.42 (1.17, 1.71)	1.29 (1.15, 1.46)	–	–
77	Rodrigues 2013	Spain	Europe	Caucasian	444/454	PB	HB	Blood samples	Non-cancer controls	Sex and Ethnicity	NR	245	177	22	283	155	16	0.3501	16	–	–	–	–
78	Wang 2013	China	Asia	Asian	600/600	HB	PB	Blood samples	Healthy controls	Age and Study region	Age, BMI and Family history of cancer	160	301	139	109	308	183	0.2976	21	–	0.53 (0.37, 0.74)	–	–
79	Krivokuca 2014	Serbia	Europe	Caucasian	155/114	HB	HB	NR	Healthy controls	Age	NR	87	58	10	62	45	7	0.7570	11	–	–	–	–
80	Saadatian 2014	Iran	Asia	Caucasian	100/100	NR	NR	Blood samples	Healthy controls	NR	NR	22	48	30	13	63	24	0.0059	8	–	–	–	–
81	Sharma 2014	India	Asia	Indian	200/200	NR	HB	Blood samples	Healthy controls	Age	NR	47	103	50	67	91	42	0.2853	14	–	–	–	–
82	Arfaoui 2015	Tunisia	Africa	Caucasian	175/159	NR	HB	Blood samples	Healthy controls	Age, Ethnicity and Origin	NR	19	62	94	69	71	19	0.9105	12	–	–	–	–
83	Devi 2015	India	Asia	Indian	462/770	PB	HB	Blood samples	Healthy controls	Age group and Ethnicity	Age, Age at menarche and Age at menopause	79	265	118	153	411	206	0.0439	17	1.34 (0.94, 1.92)	1.16 (0.77, 1.76)	1.28 (0.91, 1.81)	0.93 (0.68, 1.27)
84	Gohari-Lasaki 2015	Iran	Asia	Caucasian	100/100	NR	HB	Blood samples	Healthy controls	NR	NR	31	48	21	31	57	12	0.0677	9	–	–	–	–
85	Vymetalkova 2015	Czech	Europe	Caucasian	700/611	HB	HB	Blood samples	Non-cancer controls	NR	Age	370	275	55	301	260	50	0.5556	16	0.89 (0.70, 1.14)	0.93 (0.61, 1.43）	0.90 (0.71, 1.14)	–
86	Almeida 2016	Brazil	South America	Mixed	188/205	NR	NR	Blood samples	Non-cancer controls	Population, Ethnicity, and Geographic region	NR	79	80	29	85	87	33	0.1837	9	–	–	–	–
87	Golmohammadi 2016	Iran	Asia	Caucasian	80/80	NR	NR	Blood samples	Healthy controls	NR	NR	49	29	2	51	15	14	0.0000	6	–	–	–	–
88	Shabnaz 2016	Bangladesh	Asia	Indian	310/250	HB	HB	Blood samples	Non-cancer controls	NR	NR	97	155	58	110	104	36	0.1636	15	–	–	–	–
89	Yadav 2016	India	Asia	Indian	100/100	NR	HB	Blood samples	Non-cancer controls	Age	NR	26	55	19	47	42	11	0.7269	13	–	–	–	–
90	Hossain 2017	Bangladesh	Asia	Indian	125/125	BV	BV	Blood samples	Healthy controls	Age	NR	54	42	29	61	51	13	0.6318	15	–	–	–	–
91	Ayoubi 2018	Morocco	Africa	Caucasian	125/126	BV	HB	Blood samples	Healthy controls	NR	NR	55	42	28	65	46	15	0.1344	13	–	–	–	–
92	Chen 2018	China	Asia	Asian	727/671	PB	HB	Blood samples	Non-cancer controls	NR	NR	205	386	136	227	327	117	0.9673	15	–	–	–	–
93	Habyarimana 2018	Rwanda	Africa	African	40/39	NR	HB	Blood samples	Healthy controls	NR	NR	2	32	6	0	36	3	0.0000	7	–	–	–	–
94	Hao 2018	China	Asia	Asian	254/252	PB	HB	Blood samples	Healthy controls	NR	Age	66	149	39	82	123	47	0.9417	16	1.43 (0.93, 2.18)	1.11 (0.63, 1.95)	1.34 (0.89, 2.02)	0.88 (0.54, 1.44)
95	Afzaljavan 2020	Iran	Asia	Caucasian	308/314	NR	NR	Blood samples	Healthy controls	NR	NR	187	94	27	189	101	24	0.0486	8	–	–	–	–
96	Icen-Taskin 2020	Turkey	Asia	Caucasian	96/96	NR	HB	Tumor tissue	Non-cancer controls	Age and Ethnicity	NR	42	29	25	32	47	17	0.9713	11	–	–	–	–
97	Isakova 2020	Kyrgyzstan	Asia	Asian	103/102	NR	HB	Blood samples	NR	NR	NR	49	45	9	53	36	13	0.0940	11	–	–	–	–
98	Pouladi 2020	Iran	Asia	Caucasian	200/179	NR	NR	Blood samples	Healthy controls	NR	NR	87	84	29	64	81	34	0.3572	9	–	–	–	–
99	Akhter 2021	India	Asia	Indian	115/115	NR	NR	Blood samples	Healthy controls	Age	NR	61	25	29	31	71	13	0.0044	7	–	–	–	–

No.	First Author/Year	Country	Geographic region	Ethnicity	Sample size (case/control)	Source of controls	Source of cases	Source of genotyping material of case	Type of control	Matching	Adjustments	Genotypes distribution of IVS3 16bp						HWE	Quality score	Adjusted OR and 95% CI			
												Cases			Controls					CG vs CC	GG vs CC	GG + CG vs CC	GG vs CC + CG
												CC	CG	GG	CC	CG	GG						
1	Campbell 1996	UK	Europe	Caucasian	208/113	NR	NR	NR	Non-cancer controls	NR	NR	161	44	3	82	28	3	0.745	4	–	–	–	–
2	Själander 1996	Sweden	Europe	Caucasian	212/689	PB	PB	Whole blood or washed and Homogenized placental tissue	NR	Age and Sex	NR	162	46	4	529	142	18	0.0276	16	–	–	–	–
3	Weston 1997	USA	North America	Caucasian	65/117	PB	PB	Blood samples	Non-cancer controls	Age, Race and Diagnosis	NR	41	21	3	93	23	1	0.7452	15	–	–	–	–
4	Weston 1997	USA	North America	Spanish	18/38	PB	PB	Blood samples	Non-cancer controls	Age, Race and Diagnosis	NR	15	3	0	21	16	1	0.3098	15	–	–	–	–
5	Weston 1997	USA	North America	African	16/30	PB	PB	Blood samples	Non-cancer controls	Age, Race and Diagnosis	NR	4	12	0	13	15	2	0.3949	15	–	–	–	–
6	Khaliq 2000	Pakistan	Asia	Indian	689/41	PB	NR	Blood samples	NR	NR	NR	410	226	54	22	18	1	0.2230	12	–	–	–	–
7	Wang-Gohrke 2002	Germany	Europe	Caucasian	563/549	PB	PB	Blood samples	Non-cancer controls	Age and Study region	Number of full-term pregnancies (0, 1±2, > 3), Age at menarche (< 12, > 13), Duration of breastfeeding (continuous), Menopausal status, and Family history	370	173	20	391	145	13	0.9184	19	1.3 (1.0, 1.7)	1.7 (0.8, 3.4)	–	–
8	Suspitsin 2003	Russia	Europe	Caucasian	529/393	BV	HB	Peripheral leukocytes	Healthy controls	NR	NR	408	108	13	292	94	7	0.8582	14	–	–	–	–

Supplemental Table 6 continued

9	Buyru 2007	Turkey	Asia	Caucasian	115/63	NR	NR	Blood samples	Healthy controls	Age	NR	83	28	4	47	15	1	0.8741	8	–	–	–	–
10	Zhang 2007	China	Asia	Asian	83/269	PB	PB	Blood samples	Non-cancer controls	Age	Age, Marriage status, Culture degree and occupation	81	2	0	253	15	1	0.1450	20	1.32 (0.43, 4.06)	–	1.22 (0.40, 3.70)	–
11	Cavallone 2008	France	Europe	Caucasian	157/112	PB	FB	Blood samples	Non-cancer controls	NR	NR	102	53	2	79	32	1	0.2461	14	–	–	–	–
12	Costa 2008a	Portugal	Europe	Caucasian	70/440	BV	FB	Blood samples	Healthy controls	Age	Age	46	15	9	299	130	11	0.4779	18	0.80 (0.43, 1.49)	4.40 (1.60, 12.0)	–	–
13	Costa 2008b	Portugal	Europe	Caucasian	191/216	BV	HB	Blood samples	Healthy controls	Age	Age	122	56	13	147	65	4	0.2935	17	1.07 (0.67, 1.70)	3.88 (1.18, 12.8)	–	–
14	De 2008	Italy	Europe	Caucasian	350/352	BV	FB	NR	Healthy controls	NR	Age	233	103	14	256	87	9	0.6232	12	1.17 (0.79, 1.72)	2.67 (0.97, 7.33)	–	–
15	Gaudet 2008	USA	North America	Mixed	578/390	PB	PB	Blood samples	Non-cancer controls	Age	Age	404	157	17	272	108	10	0.8528	21	0.96 (0.72, 1.29)	1.16 (0.52, 2.59)	–	–
16	Akkiprik 2009	Turkey	Asia	Caucasian	97/107	NR	HB	Tumor tissue	Healthy controls	Age	NR	59	35	3	61	43	3	0.1530	12	–	–	–	–
17	Hrstka 2009	Czech	Europe	Caucasian	117/108	BV	HB	Tumor tissue	Healthy controls	NR	NR	81	32	4	81	24	3	0.4608	13	–	–	–	–
18	Ma 2009	China	Asia	Asian	117/123	HB	HB	Blood samples	Healthy controls	NR	NR	102	15	0	117	6	0	0.7816	13	–	–	–	–
19	Bisof 2010	Croatia	Europe	Caucasian	95/108	PB	HB	Tumor tissue	Healthy controls	Age and Place of birth	NR	67	21	7	77	31	0	0.0816	14	–	–	–	–
20	Jakubowska 2010	Poland	Europe	Caucasian	311/287	PB	PB	Blood samples	Non-cancer controls	NR	Age at first live birth, Parity, lifetime cumulative months of breastfeeding, Age at menarche, Oral contraceptive use, Smoking, Body mass index (at age of breast cancer diagnosis for cases and at time of interview for controls), Year of birth and BRCA1 mutation	208	103		195	92		–	18	–	–	1.21 (0.81, 1.83)	–
21	Trifa 2010	Tunisia	Africa	Caucasian	109/132	BV	HB	Blood samples	Healthy controls	NR	NR	66	38	5	86	41	5	0.9671	12	–	–	–	–
22	Alawadi 2011	Arabia	Asia	Caucasian	229/133	PB	HB	Blood samples	Healthy controls	NR	NR	97	121	11	65	54	14	0.5800	12	–	–	–	–
23	Faghani 2011	Iran	Asia	Caucasian	145/145	HB	HB	Blood samples	Non-cancer controls	Age	NR	21	124	0	62	83	0	>0.000	12	–	–	–	–
24	Cherdyntseva 2012	Russia	Europe	Caucasian	296/196	BV	HB	Blood samples	Non-cancer controls	Age and Ethnicity	NR	227	68	1	145	50	1	0.1284	17	–	–	–	–
25	Guleria 2012	India	Asia	Indian	80/80	NR	HB	Blood samples	Healthy controls	Age and Gender	NR	43	30	7	53	25	2	0.6360	14	–	–	–	–
26	Lajin 2013	Syria	Asia	Caucasian	122/139	PB	HB	Blood samples	Non-cancer controls	NR	NR	54	60	8	76	48	15	0.0882	14	–	–	–	–
27	Marouf 2014	Morocco	Africa	Caucasian	105/114	BV	HB	Blood samples	Healthy controls	NR	NR	73	28	4	78	28	8	0.0240	11	–	–	–	–
28	Pouladi 2014	Iran	Asia	Caucasian	221/170	PB	HB	Tumor tissue	Non-cancer controls	NR	NR	135	69	17	107	51	12	0.0963	13	–	–	–	–
29	Sharma 2014	India	Asia	Indian	200/200	NR	HB	Blood samples	Healthy controls	Age	NR	134	52	14	137	55	8	0.4106	14	–	–	–	–
30	Eskandari-Nasab 201	Iran	Asia	Caucasian	236/203	NR	NR	Blood samples	Healthy controls	Age	NR	108	88	40	113	67	23	0.0110	9	–	–	–	–
31	Gohari-Lasaki 2015	Iran	Asia	Caucasian	100/100	NR	HB	Blood samples	Healthy controls	NR	NR	53	38	9	60	37	3	0.3364	9	–	–	–	–
32	Vymetalkova 2015	Czech	Europe	Caucasian	662/611	HB	HB	Blood samples	Non-cancer controls	NR	Age	474	164	24	421	172	18	0.9320	16	0.88 (0.67, 1.16)	1.13 (0.57, 2.24)	0.91 (0.70, 1.18)	–
33	Hao 2018	China	Asia	Asian	254/252	PB	HB	Blood samples	Healthy controls	NR	Age	230	24	0	227	25	0	0.4074	16	0.84 (0.44, 1.58)	–	0.84 (0.44, 1.58)	–
34	Morten 2019	Australia	Oceania	Caucasian	1304/436	PB	PB	Blood samples	Non-cancer controls	NR	Population subsets and Age	986	289	29	325	104	7	0.6872	19	–	–	–	–
35	Diakite 2020	Mali	Africa	Caucasian	60/60	PB	HB	Blood samples	Healthy controls	Age	NR	27	25	8	39	16	5	0.0968	15	–	–	–	–

No.	First Author/Year	Country	Geographic region	Ethnicity	Sample size (case/control)	Source of controls	Source of cases	Source of genotyping material of case	Type of control	Matching	Adjustments	Genotypes distribution of IVS6+62A>G						HWE	Quality score	Adjusted OR and 95% CI			
												Cases			Controls								
												CC	CG	GG	CC	CG	GG			CG vs CC	GG vs CC	GG + CG vs CC	GG vs CC + CG
1	Peller 1995	Israel	Asia	Caucasian	30/38	NR	NR	White blood cells (WBC)	Healthy controls	NR	NR	24	5	1	26	12	0	0.248	8	–	–	–	–
2	Själander 1996	Sweden	Europe	Caucasian	212/689	PB	PB	Whole blood or washed and Homogenized placental tissue	NR	Age and Sex	NR	161	48	3	525	146	18	0.0468	16	–	–	–	–
3	Weston 1997	USA	North America	Caucasian	65/117	PB	PB	Blood samples	Non-cancer controls	Age, Race and Diagnosis	NR	43	20	2	95	22	0	0.2617	15	–	–	–	–
4	Weston 1997	USA	North America	Spanish	18/38	PB	PB	Blood samples	Non-cancer controls	Age, Race and Diagnosis	NR	16	2	0	23	13	2	0.9265	15	–	–	–	–
5	Weston 1997	USA	North America	African	16/30	PB	PB	Blood samples	Non-cancer controls	Age, Race and Diagnosis	NR	3	13	0	12	16	2	0.2733	15	–	–	–	–
6	Mavridou 1998	UK	Europe	Caucasian	224/254	NR	NR	NR	Non-cancer controls	NR	NR	184	39	1	208	42	4	0.2764	5	–	–	–	–
7	Khaliq 2000	Pakistan	Asia	Indian	689/41	PB	NR	Blood samples	NR	NR	NR	65	253	371	2	17	22	0.5722	12	–	–	–	–
8	Wang-Gohrke 2002	Germany	Europe	Caucasian	563/548	PB	PB	Blood samples	Non-cancer controls	Age and Study region	Number of full-term pregnancies (0, 1±2, > 3), Age at menarche (< 12, > 13), Duration of breastfeeding (continuous), Menopausal status, and Family history	388	157	18	399	139	10	0.5974	19	1.2 (0.9, 1.6)	2.0 (0.9, 4.5)	–	–
9	Suspitsin 2003	Russia	Europe	Caucasian	565/393	BV	HB	Peripheral leukocytes	Healthy controls	NR	NR	462	94	9	296	91	6	0.7401	14	–	–	–	–
10	Wirtenberger 2006	Germany	Europe	Caucasian	807/998	BV	FB	NR	Healthy controls	NR	NR	587	195	25	756	227	15	0.6628	14	–	–	–	–
11	Baynes 2007	UK	Europe	Caucasian	2042/2197	PB	PB	Blood samples	Non-cancer controls	U	NR	1545	449	48	1622	520	55	0.0875	18	–	–	–	–
12	Buyru 2007	Turkey	Asia	Caucasian	115/63	NR	NR	Blood samples	Healthy controls	Age	NR	107	7	1	56	6	1	0.1139	8	–	–	–	–
13	Garcia-Closas 2007	Norway and Poland	Europe	Caucasian	2681/3382	HB	HB	Blood samples	Non-cancer controls	Age	Age and Study population	2080	564	37	2686	641	55	0.0204	16	1.10 (0.96, 1.25)	0.89 (0.58, 1.37)	–	–
14	Sprague 2007	USA	North America	Caucasian	1648/1846	PB	PB	Buccal sample	Non-cancer controls	Age	NR	1254	359	35	1358	438	50	0.0434	17	–	–	–	–
15	Zhang 2007	China	Asia	Asian	83/268	PB	PB	Blood samples	Non-cancer controls	Age	Age, Marriage status, Culture degree and occupation	75	8	0	240	25	3	0.0184	18	1.54 (0.65, 3.65)	2.64 (0.22, 31.78)	1.62 (0.71, 3.69)	–
16	Gaudet 2008	USA	North America	Mixed	578/390	PB	PB	Blood samples	Non-cancer controls	Age	Age	412	152	14	282	99	9	0.9288	21	1.03 (0.76, 1.39)	1.02 (0.43, 2.40)	–	–
17	Singh 2008	India	Asia	Indian	104/105	HB	HB	Blood samples	Healthy controls	Study region	NR	80	20	4	74	28	3	0.8587	11	–	–	–	–
18	Akkiprik 2009	Turkey	Asia	Caucasian	99/107	NR	HB	Tumor tissue	Healthy controls	Age	NR	51	39	9	61	38	8	0.5431	12	–	–	–	–
19	Hrstka 2009	Czech	Europe	Caucasian	117/108	BV	HB	Tumor tissue	Healthy controls	NR	NR	76	39	2	83	23	2	0.7834	13	–	–	–	–
20	Jakubowska 2010	Poland	Europe	Caucasian	319/290	PB	PB	Blood samples	Non-cancer controls	NR	Age at first live birth, Parity, lifetime cumulative months of breastfeeding, Age at menarche, Oral contraceptive use, Smoking, Body mass index (at age of breast cancer diagnosis for cases and at time of interview for controls), Year of birth and BRCA1 mutation	230	82	7	208	76	6	0.7578	18	1.08 (0.69, 1.67)	3.64 (0.68, 19.35)	–	–
21	Cherdyntseva 2012	Russia	Europe	Caucasian	393/193	BV	HB	Blood samples	Non-cancer controls	Age and Ethnicity	NR	293	94	6	147	45	1	0.2102	17	–	–	–	–
22	Lajin 2013	Syria	Asia	Caucasian	122/139	PB	HB	Blood samples	Non-cancer controls	NR	NR	58	56	8	78	48	13	0.1712	14	–	–	–	–
23	Rodrigues 2013	Spain	Europe	Caucasian	446/365	PB	HB	Blood samples	Non-cancer controls	Sex and Ethnicity	NR	334	108	4	289	71	5	0.7883	16	–	–	–	–
24	Sharma 2014	India	Asia	Indian	200/200	NR	HB	Blood samples	Healthy controls	Age	NR	124	66	10	135	56	9	0.3122	14	–	–	–	–
25	Assad 2019	Iran	Asia	Caucasian	86/96	HB	HB	Blood samples	NR	NR	NR	60	20	6	30	48	18	0.8764	8	–	–	–	–
