# Supplementary material for: Association Between the TP53 Polymorphisms and Breast Cancer Risk: An Updated Meta-Analysis
Source: Front Genet. 2022 Apr 27;13:807466. doi: 10.3389/fgene.2022.807466 (PMC9091657; doi:10.3389/fgene.2022.807466)
Supplement: Supplementary file 5 [file DataSheet3.PDF]

Supplemental Table 3. Included studies of TP53 IVS3 16bp (rs17878362) polymorphism in BC risk within the meta-analyses (A, Asian; I: Indian; Af, African; C, Caucasian; ME, Middle East; H, Hispanic; M, mixed; U, unidentified).

No.	First Author/Year	Country	All studies							
			**This study, 2021**	Diakite 2020	Wu 2013	Sagne 2013	He 2011	Hu 2010(2)	Hu 2010(1)	Dunning 1999
1	Campbell 1996	UK	C	–	–	–	–	–	–	U
2	Själander 1996	Sweden	C(not in HWE)	–	U(not in HWE)	–	C	European	–	White Swedish
3	Weston 1997	USA	C	M	European	–	C	European	C	–
4	Weston 1997	USA	H		–	–	H			–
5	Weston 1997	USA	Af		–	–	Af			–
6	Wang-Gohrke 1998	Germany	Exclude	–	U	–	C	–	C	U
7	Khaliq 2000	Pakistan	I	–	–	–	–	–	–	–
8	Wang-Gohrke 2002	Germany	C	Germany	–	Northern Europe	–	European	–	–
9	Suspitsin 2003	Russian	C	Russia	European	–	C	European	C	–
10	Buyru 2007	Turkey	C	Turkey	U	–	C	European	C	–
11	Zhang 2007	China	A	–	A	–	–	–	–	–
12	Cavallone 2008	France	C	–	European	–	C	European	C	–
13	Costa 2008a	Portugal	C	Portugal	U	Mediterranean	C	European	C	–
14	Costa 2008b	Portugal	C							–
15	De 2008	Italy	C	Italy	U	Mediterranean	C	–	C	–
16	Gaudet 2008	USA	M	M	U	USA	M	M	M	–
17	Akkiprik 2009	Turkey	C	Turkey	European	–	C	–	C	–
18	Hrstka 2009	Czech	C	Island	U	Northern Europe	C	–	–	–
19	Ma 2009	China	A	–	A	–	–	–	–	–
20	Bisof 2010	Croatia	C	–	U	–	C	–	–	–
21	Jakubowska 2010	Poland	C	–	European	–	–	–	–	–
22	Trifa 2010	Tunisia	C	Tunisia	Af	–	–	–	–	–
23	Alawadi 2011	Arabia	C	–	A	U	–	–	–	–
24	Faghani 2011	Iran	C(not in HWE)	–	A(not in HWE)	–	–	–	–	–
25	Cherdyntseva 2012	Russia	C	Russia	European	–	–	–	–	–
26	Guleria 2012	India	I	India	–	–	–	–	–	–
27	Lajin 2013	Syria	C	–	–	–	–	–	–	–
28	Marouf 2014	Morocco	C(not in HWE)	–	–	–	–	–	–	–
29	Pouladi 2014	Iran	C	Iran	–	–	–	–	–	–
30	Sharma 2014	India	I	India	–	–	–	–	–	–
31	Eskandari-Nasab 2015	Iran	C(not in HWE)	–	–	–	–	–	–	–
32	Gohari-Lasaki 2015	Iran	C	Iran	–	–	–	–	–	–
33	Vymetalkova 2015	Czech	C	Czech	–	–	–	–	–	–
34	Hao 2018	China	A	Chine	–	–	–	–	–	–
35	Morten 2019	Australia	C	Australia	–	–	–	–	–	–
36	Diakite 2020	Mali	C	–	–	–	–	–	–	–
