# Supplementary material for: Association Between the TP53 Polymorphisms and Breast Cancer Risk: An Updated Meta-Analysis
Source: Front Genet. 2022 Apr 27;13:807466. doi: 10.3389/fgene.2022.807466 (PMC9091657; doi:10.3389/fgene.2022.807466)
Supplement: Supplementary file 6 [file DataSheet1.PDF]

Supplemental Table 1 Scale for quality assessment of molecular association studies of BC

Criterion	Score
Source of case	
Selected from population or cancer registry	3
Selected from hospital	2
Selected from pathology archives, but without description	1
Not described	0
Source of control	
Population-based	3
Blood donors or volunteers	2
Hospital-based	1
Not described	0
Ascertainment of cancer	
Histological or pathological confirmation	2
Diagnosis of BC by patient medical record	1
Not described	0
Ascertainment of control	
Controls were tested to screen out BC	2
Controls were subjects who did not report BC, no objective testing	1
Not described	0
Matching	
Controls matched with cases by age	2
Not matched or not described	0
Source of genotyping material of case	
Appropriate DNA sources (such as peripheral blood, buccal swabs and saliva, and so on)	2
Tumor tissue	1
Not described	0
Genotyping examination	
Genotyping done blindly and quality control	2
Only genotyping done blindly or quality control	1
Unblinded and without quality control	0
HWE	
HWE in the control group	2
Hardy-Weinberg disequilibrium in the control group	0
Association assessment	
Assess association between genotypes and BC with appropriate statistics and adjustment for confounders	2
Assess association between genotypes and BC with appropriate statistics without adjustment for confounders	1
Inappropriate statistics used	0
Total sample size	
>1000	3
500-1000	2
200-500	1
<200	0

HWE: Hardy-Weinberg equilibrium, BC: Breast cancer
