# Supplementary material for: Association Between the TP53 Polymorphisms and Breast Cancer Risk: An Updated Meta-Analysis
Source: Front Genet. 2022 Apr 27;13:807466. doi: 10.3389/fgene.2022.807466 (PMC9091657; doi:10.3389/fgene.2022.807466)
Supplement: Supplementary file 7 [file DataSheet5.PDF]

Supplemental Table 5. Results of previous meta-analyses between TP53 polymorphism and BC risk

First author/year	Variable	n (Cases/Controls)	G vs. C		CG vs. CC		GG vs. CC		CG + GG vs. CC		GG vs. CC + CG	
			OR (95% CI)	P_h/I^2 (%)	OR (95% CI)	P_h/I^2 (%)	OR (95% CI)	P_h/I^2 (%)	OR (95% CI)	P_h/I^2 (%)	OR (95% CI)	P_h/I^2 (%)
**Codon 72 (rs1042522)**												
Dunning 1999	Overall	3	1.27 (1.02, 1.59)	–	1.23 (1.02, 1.58)	–	1.12 (0.74, 1.65)	–	–	–	–	–
Suspitsin 2003	Overall	7	–	–	–	–	–	–	1.14 (1.00, 1.30)	–	–	–
Zhuo 2009	Overall	17 (12,226/10,782)	–	–	–	–	0.83 (0.67, 1.04)	0.000	0.88 (0.76, 1.02)	0.000	0.89 (0.76, 1.04)	0.010
	Caucasian	(11,549/9,830)	–	–	–	–	0.87 (0.69, 1.10)	0.001	0.92 (0.79, 1.08)	0.000	0.90 (0.77, 1.05)	0.060
	Asian	(631/873)	–	–	–	–	0.74 (0.33, 1.64)	0.000	0.82 (0.49, 1.39)	0.002	0.84 (0.48, 1.49)	0.006
	African	(46/79)	–	–	–	–	0.68 (0.18, 2.56)	0.760	0.60 (0.29, 1.25)	0.22	0.85 (0.24, 3.03)	0.450
Hu 2010(2)	Overall	37 (23,567/2,5995)	–	–	–	–	0.96 (0.85, 1.08)	0.000	0.96 (0.88, 1.03)	0.000	0.98 (0.89, 1.09)	0.010
	Asian	11 (2,270/2,848)	–	–	–	–	0.87 (0.73, 1.03)	0.000	0.94 (0.77, 1.16)	0.004	0.91 (0.71, 1.19)	0.010
	Mediterranean	6 (375/389)	–	–	–	–	0.35 (0.21, 0.60)	0.220	0.32 (0.24, 0.44)	0.006	0.73 (0.44, 1.19)	0.070
	N. European	20 (17,149/1,8173)	–	–	–	–	1.01 (0.93, 1.10)	0.250	1.01 (0.97, 1.06)	0.190	1.01 (0.93, 1.09)	0.460
	American	4 (3,773/4,585)	–	–	–	–	1.06 (0.89, 1.27)	0.070	1.08 (0.99, 1.18)	0.200	1.03 (0.86, 1.22)	0.080
Zhang 2010	Overall	39 (26,041/29,679)	–	–	0.91 (0.83, 1.00)	0.000	0.92 (0.82, 1.04)	0.000	0.90 (0.82, 0.99)	0.000	0.95 (0.87, 1.04)	0.032
	Asian	9	–	–	1.04 (0.86, 1.25)	0.216	0.92 (0.65, 1.31)	0.013	1.01 (0.82, 1.25)	0.065	0.93 (0.70, 1.23)	0.059
	European	29	–	–	0.89 (0.80, 0.99)	0.000	0.92 (0.81, 1.05)	0.000	0.88 (0.80, 0.98)	0.000	0.96 (0.87, 1.06)	0.028
	African	1	–	–	0.36 (0.13, 0.99)	–	0.79 (0.16, 4.04)	–	0.42 (0.17, 1.07)	–	1.25 (0.26, 6.02)	–
Francisco 2011	Overall	42 (23,429/28,000)	–	–	–	–	0.97 (0.87, 1.08)	–/44.8	0.95 (0.88, 1.03)	–/70.6	1.01 (0.95, 1.08)	–/11.6
	Caucasian	18 (15,791/18,308)	–	–	–	–	1.06 (0.98, 1.16)	–	1.02 (0.98, 1.07)	–	1.07 (0.98, 1.16)	–
	Asian	5 (1,281/1,399)	–	–	–	–	1.15 (0.91, 1.46)	–	1.07 (0.90, 1.26)	–	1.14 (0.91, 1.41)	–
	Indian	5 (715/1,668)	–	–	–	–	0.70 (0.53, 0.91)	–	0.75 (0.61, 0.93)	–	0.77 (0.61, 0.97)	–
	Mixed Population	11 (5,297/6,330)	–	–	–	–	0.95 (0.82, 1.10)	–	0.97 (0.90, 1.05)	–	0.99 (0.86, 1.14)	–
He 2011	Overall	52 (27,046/30,998)	–	–	–	–	0.95 (0.85, 1.07)	0.000	0.97 (0.90, 1.05)	0.000	0.96 (0.88, 1.06)	0.009
	Caucasian	27 (21,017/22,726)	–	–	–	–	0.97 (0.84, 1.12)	0.000	1.00 (0.90, 1.10)	0.000	0.97 (0.84, 1.12)	0.049
	Asian	17 (3,611/5,024)	–	–	–	–	0.94 (0.74, 1.20)	0.001	0.98 (0.84, 1.14)	0.010	0.95 (0.79, 1.15)	0.010
	Indian	4 (674/979)	–	–	–	–	0.62 (0.46, 0.82)	0.153	0.68 (0.45, 1.03)	0.037	0.70 (0.50, 0.92)	0.463
Ma 2011	Overall	21 (22,515/22,388)	–	–	–	–	0.80 (0.64, 1.00)	0.000	0.85 (0.73, 0.98)	0.000	0.87 (0.75, 1.02)	0.008
	Caucasian	10	–	–	–	–	0.86 (0.66, 1.12)	0.001	0.90 (0.75, 1.09)	0.000	0.91 (0.76, 1.08)	0.060
	Asian	7	–	–	–	–	0.83 (0.43, 1.59)	0.001	0.90 (0.62, 1.32)	0.007	0.86 (0.52, 1.43)	0.002
	African	2	–	–	–	–	0.68 (0.18, 2.63)	0.756	0.63 (0.25, 1.59)	0.223	0.88 (0.24, 3.23)	0.452
Cheng 2012	Overall	(19,075/19,291)	–	–	0.96 (0.89, 1.05)	0.000	0.96 (0.83, 1.10)	0.000	0.96 (0.88, 1.04)	0.000	0.96 (0.86, 1.07)	0.020
	Asian	(1,617/1,881)	–	–	1.03 (0.81, 1.30)	0.083	0.92 (0.60, 1.43)	0.000	1.00 (0.75, 1.32)	0.004	0.92 (0.67, 1.26)	0.009
	Caucasian	(17,163/16,939)	–	–	0.98 (0.89, 1.07)	0.000	0.99 (0.86, 1.15)	0.003	0.98 (0.89, 1.07)	0.000	0.99 (0.88, 1.10)	0.075
	African	(205/343)	–	–	0.78 (0.53, 1.14)	0.399	0.73 (0.42, 1.27)	0.761	0.76 (0.53, 1.09)	0.550	0.83 (0.50, 1.39)	0.600
	Hispanic	(18/38)	–	–	1.67 (0.36, 7.81)	–	1.94 (0.40, 9.55)	–	1.79 (0.43, 7.50)	–	1.38 (0.43, 4.44)	–
	Mixed	(72/90)	–	–	0.41 (0.21, 0.79)	–	0.32 (0.08, 1.35)	–	0.40 (0.21, 0.76)	–	0.52 (0.13, 2.07)	–
Dahabreh 2013	Overall	59 (29,801/35,436)	0.99 (0.94, 1.03)	0.532	–	–	–	–	–	–	–	–
	Whites	40 (25,469/29,930)	1.01 (0.96, 1.06)	0.609	–	–	–	–	–	–	–	–
	East Asians	7 (2,859/2,880)	1.04 (0.92, 1.16)	0.525	–	–	–	–	–	–	–	–
Hou 2013	Overall	58 (28,539/32,788)	1.00 (0.98, 1.03)	0.000	1.02 (0.93, 1.11)	0.007	1.03 (0.92, 1.15)	0.000	1.02 (0.93, 1.11)	0.001	1.03 (0.95, 1.12)	0.000
	Asian	20	1.02 (0.96, 1.08)	0.000	1.03 (0.89, 1.19)	0.082	1.04 (0.84, 1.27)	0.001	1.03 (0.88, 1.20)	0.012	1.01 (0.88, 1.16)	0.010
	Caucasian	31	1.00 (0.97, 1.03)	0.000	1.05 (0.93, 1.18)	0.473	1.03 (0.88, 1.20)	0.000	1.04 (0.93, 1.16)	0.035	1.02 (0.92, 1.13)	0.000
	Others	8	0.96 (0.88, 1.04)	0.000	0.88 (0.65, 1.20)	0.160	1.02 (0.67, 1.55)	0.042	0.91 (0.65, 1.28)	0.064	1.17 (0.85, 1.60)	0.000
Gonçalves 2014	Overall	41 (25,629/26,633)	1.02 (1.00, 1.05)	–	1.05 (0.99, 1.12)	–	1.05 (0.99, 1.12)	–	1.06 (0.99, 1.12)	–	1.05 (0.98, 1.05)	–
	African	2 (189/181)	1.16 (0.86, 1.56)	–	0.99 (0.54, 1.83)	–	1.30 (0.69, 2.44)	–	1.05 (0.60, 1.86)	–	1.32 (0.86, 2.01)	–
	America	6 (6,483/8,011)	0.98 (0.92, 1.02)	–	1.01 (0.88, 1.16)	–	1.04 (0.82, 1.33)	–	0.98 (0.86, 1.11)	–	0.96 (0.90, 1.03)	–
	Asian	14 (2,570/2,833)	1.09 (1.01, 1.17)	–	1.15 (0.85, 1.57)	–	1.24 (0.87, 1.78)	–	1.23 (1.07, 1.41)	–	1.04 (0.93, 1.17)	–
	Europe	19 (16,387/15,608)	1.02 (0.99, 1.06)	–	1.01 (0.92, 1.11)	–	1.10 (0.93, 1.31)	–	1.04 (0.95, 1.13)	–	1.03 (0.98, 1.08)	–
Diakite 2020	Overall	21 (7,841/8,876)	1.09 (1.01, 1.17)	0.010/47	–	–	–	–	1.09 (1.02, 1.16)	0.090/31	1.07 (0.97, 1.18)	0.050/36
	Caucasian	11 (5,472/6,396)	1.07 (1.01, 1.14)	0.090/39	–	–	–	–	1.09 (1.01, 1.17)	0.190/27	1.09 (0.95, 1.25)	0.340/10
	Asian	9 (2,244/2,354)	1.06 (0.91, 1.23)	0.030/54	–	–	–	–	1.06 (0.94, 1.20)	0.070/45	1.01 (0.87, 1.17)	0.050/48
	African	1 (125/126)	1.49 (1.03, 2.16)	–	–	–	–	–	1.36 (0.83, 2.23)	–	2.14 (1.08, 4.23)	–
**IVS3 16bp (rs17878362)**												
Dunning 1999	Overall	3	–	–	1.02 (0.79, 1.31)	–	0.89 (0.39, 1.79)	–	–	–	–	–
Hu 2010(1)	Overall	9 (2715/2595)	–	–	1.10 (0.97, 1.25)	0.350	1.81 (1.30, 2.52)	0.300	1.16 (1.03, 1.31)	0.280	–	–
Hu 2010(2)	Overall	8 (2,470/2,825)	–	–	–	–	1.70 (1.20, 2.37)	0.120	1.15 (1.01, 1.30)	0.280	–	–
He 2011	Overall	14 (3,332/3,700)	–	–	–	–	1.66 (1.24, 2.21)	0.280	1.14 (1.02, 1.27)	0.300	1.61 (1.21, 2.15)	0.250
Sagne 2013	Overall	6 (2,028/1,748)	–	–	1.18 (1.02, 1.37)	0.570	1.41 (0.97, 2.06)	0.080	–	–	–	–
Wu 2013	Overall	19 (4,479/4,683)	–	–	1.18 (1.00, 1.40)	0.001	1.42 (1.09, 1.84)	0.079	1.21 (1.03, 1.41)	0.001	1.28 (0.87, 1.89)	0.038
	Asian	4	–	–	1.76 (0.73, 4.28)	0.001	0.53 (0.23, 1.23)	–	1.67 (0.66, 4.24)	0.000	0.43 (0.19, 0.97)	–
	European	6	–	–	1.04 (0.84, 1.28)	0.273	1.07 (0.60, 1.91)	0.699	1.05 (0.84, 1.32)	0.172	1.09 (0.62, 1.92)	0.774
	African	1	–	–	1.20 (0.73, 1.97)	–	0.88 (0.25, 3.13)	–	1.16 (0.72, 1.88)	–	0.82 (0.23, 2.91)	–
Diakite 2020	Overall	18	1.11 (1.02, 1.19)	0.010	–	–	–	–	1.07 (0.98, 1.17)	0.150	1.46 (1.15–1.85)	0.002
**IVS6+62A>G (rs1625895)**												
Dunning 1999	Overall	4	–	–	1.16 (0.91, 1.47)	–	0.84 (0.32, 1.79)	–	–	–	–	–
Hu 2010(2)	Overall	10 (8,537/9,586)	–	–	–	–	0.91 (0.74, 1.12)	0.710	1.00 (0.89, 1.13)	0.040	0.92 (0.74, 1.13)	0.710
He 2011	Overall	14 (8,787/9,869)	–	–	–	–	0.93 (0.76, 1.14)	0.800	1.03 (0.91, 1.18)	0.009	0.93 (0.76, 1.14)	0.850
	Caucasian	10 (8,071/9,306)	–	–	–	–	0.93 (0.75, 1.15)	0.500	1.01 (0.94, 1.08)	0.010	0.93 (0.75, 1.15)	0.650
