# Supplementary material for: Association Between the TP53 Polymorphisms and Breast Cancer Risk: An Updated Meta-Analysis
Source: Front Genet. 2022 Apr 27;13:807466. doi: 10.3389/fgene.2022.807466 (PMC9091657; doi:10.3389/fgene.2022.807466)
Supplement: Supplementary file 8 [file DataSheet8.DOCX]

**Appendix**

**Detailed search strategies**

("p53"[All Fields] OR "tp53"[All Fields] OR "tp-53"[All Fields] OR "p-53"[All Fields]) AND ("polymorphism, genetic"[MeSH Terms] OR "polymorphism, genetic"[All Fields] OR ("polymorphism"[All Fields] AND "genetic"[All Fields]) OR "genetic polymorphism"[All Fields] OR "polymorphism"[All Fields] OR "variant*"[All Fields] OR "variati*"[All Fields] OR "mutation"[MeSH Terms] OR "mutat*"[All Fields] OR "snp"[All Fields] OR "genome wide association study"[MeSH Terms] OR "genome wide association study"[All Fields] OR ("genome wide"[All Fields] AND "association"[All Fields] AND "study"[All Fields]) OR ("genome"[All Fields] AND "wide"[All Fields] AND "association"[All Fields] AND "study"[All Fields]) OR "genotype"[MeSH Terms] OR "genotyp*"[All Fields] OR "allel*"[All Fields]) AND ("breast"[MeSH Terms] OR "breast*"[All Fields]) AND ("rs1042522"[All Fields] OR "arg72pro"[All Fields] OR "arg72"[All Fields] OR "pro72"[All Fields] OR "pro72arg"[All Fields] OR (("codon"[MeSH Terms] OR "codon*"[All Fields]) AND "72"[All Fields]) OR (("exons"[MeSH Terms] OR "exon*"[All Fields]) AND "4"[All Fields]) OR "R72P"[All Fields] OR "rs17878362"[All Fields] OR "PIN3"[All Fields] OR "IVS3 16 bp"[All Fields] OR ("IVS3"[All Fields] AND "16"[All Fields] AND ("body posit"[Journal] OR "bp"[All Fields])) OR (("introns"[MeSH Terms] OR "intron*"[All Fields]) AND "3"[All Fields]) OR ("IVS3"[All Fields] AND "24"[All Fields] AND "Ins16"[All Fields]) OR "Ins16"[All Fields] OR "Ins16bp"[All Fields] OR ("16"[All Fields] AND ("body posit"[Journal] OR "bp"[All Fields])) OR "rs1625895"[All Fields] OR (("introns"[MeSH Terms] OR "intron*"[All Fields]) AND "6"[All Fields]) OR "MspI"[All Fields] OR "IVS6+62A＞G"[All Fields] OR ("IVS6+62A"[All Fields] AND "G"[All Fields]) OR "R.13494g"[All Fields])

**Overlapping Exclusion:**

| **Overlapping extraction** | **Reservation** |
| --- | --- |
| ***TP53* codon 72 (rs1042522)** | |
| BCAC 2006 HBCS | Tommiska 2005 |
| Wang-Gohrke 1998 | BCAC 2006 GESBC |
| Wang-Gohrke 2002 |  |
| Samson 2007 | Rajkumar 2008 |
| Akhter 2018 | Akhter 2021 |
| Isakova 2017 | Isakova 2020 |
| Denisov 2009 | Cherdyntseva 2012 |
| Buyru 2003 | Buyru 2007 |
| BCAC 2006 SEARCH | Baynes 2007 |
| Pharoah 2007 |  |
| BCAC 2006 PBSC | Garcia-Closas 2007 |
| **IVS3 16bp (rs17878362)** | |
| Wang-Gohrke 1998 | Wang-Gohrke 2002 |
| **IVS6+62A>G (rs1625895)** | |
| Wang-Gohrke 1998 | Wang-Gohrke 2002 |
| Pharoah 2007 | Baynes 2007 |

**List of eligible studies**

1. Kawajiri K, Nakachi K, Imai K, Watanabe J, Hayashi S. Germ line polymorphisms of p53 and CYP1A1 genes involved in human lung cancer. Carcinogenesis 1993, 14(6):1085-1089.
2. Peller S, Kopilova Y, Slutzki S, Halevy A, Kvitko K, Rotter V. A novel polymorphism in intron 6 of the human p53 gene: a possible association with cancer predisposition and susceptibility. DNA Cell Biol 1995, 14(12):983-990.
3. Campbell IG, Eccles DM, Dunn B, Davis M, Leake V. p53 polymorphism in ovarian and breast cancer. Lancet 1996, 347(8998):393-394.
4. Själander A, Birgander R, Hallmans G, Cajander S, Lenner P, Athlin L, Beckman G, Beckman L. p53 polymorphisms and haplotypes in breast cancer. Carcinogenesis 1996, 17(6):1313-1316.
5. Weston A, Pan CF, Ksieski HB, Wallenstein S, Berkowitz GS, Tartter PI, Bleiweiss IJ, Brower ST, Senie RT, Wolff MS. p53 haplotype determination in breast cancer. Cancer Epidemiol Biomarkers Prev 1997, 6(2):105-112.
6. Helland A, Langerød A, Johnsen H, Olsen AO, Skovlund E, Børresen-Dale AL. p53 polymorphism and risk of cervical cancer. Nature 1998, 396(6711):530-531; author reply 532.
7. Mavridou D, Gornall R, Campbell IG, Eccles DM. TP53 intron 6 polymorphism and the risk of ovarian and breast cancer. Br J Cancer 1998, 77(4):676-677.
8. Wang-Gohrke S, Rebbeck TR, Besenfelder W, Kreienberg R, Runnebaum IB. p53 germline polymorphisms are associated with an increased risk for breast cancer in German women. Anticancer Res 1998, 18(3b):2095-2099.
9. Khaliq S, Hameed A, Khaliq T, Ayub Q, Qamar R, Mohyuddin A, Mazhar K, Qasim-Mehdi S. P53 mutations, polymorphisms, and haplotypes in Pakistani ethnic groups and breast cancer patients. Genet Test 2000, 4(1):23-29.
10. Papadakis EN, Dokianakis DN, Spandidos DA. p53 codon 72 polymorphism as a risk factor in the development of breast cancer. Mol Cell Biol Res Commun 2000, 3(6):389-392.
11. Li T, Lu ZM, Guo M, Wu QJ, Chen KN, Xing HP, Mei Q, Ke Y. p53 codon 72 polymorphism (C/G) and the risk of human papillomavirus-associated carcinomas in China. Cancer 2002, 95(12):2571-2576.
12. Wang-Gohrke S, Becher H, Kreienberg R, Runnebaum IB, Chang-Claude J. Intron 3 16 bp duplication polymorphism of p53 is associated with an increased risk for breast cancer by the age of 50 years. Pharmacogenetics 2002, 12(3):269-272.
13. Buyru N, Tigli H, Dalay N. P53 codon 72 polymorphism in breast cancer. Oncol Rep 2003, 10(3):711-714.
14. Huang XE, Hamajima N, Katsuda N, Matsuo K, Hirose K, Mizutani M, Iwata H, Miura S, Xiang J, Tokudome S *et al*. Association of p53 codon Arg72Pro and p73 G4C14-to-A4T14 at exon 2 genetic polymorphisms with the risk of Japanese breast cancer. Breast Cancer 2003, 10(4):307-311.
15. Katiyar S, Thelma BK, Murthy NS, Hedau S, Jain N, Gopalkrishna V, Husain SA, Das BC. Polymorphism of the p53 codon 72 Arg/Pro and the risk of HPV type 16/18-associated cervical and oral cancer in India. Mol Cell Biochem 2003, 252(1-2):117-124.
16. Mabrouk I, Baccouche S, El-Abed R, Mokdad-Gargouri R, Mosbah A, Saïd S, Daoud J, Frikha M, Jlidi R, Gargouri A. No evidence of correlation between p53 codon 72 polymorphism and risk of bladder or breast carcinoma in Tunisian patients. Ann N Y Acad Sci 2003, 1010:764-770.
17. Suspitsin EN, Buslov KG, Grigoriev MY, Ishutkina JG, Ulibina JM, Gorodinskaya VM, Pozharisski KM, Berstein LM, Hanson KP, Togo AV *et al*. Evidence against involvement of p53 polymorphism in breast cancer predisposition. Int J Cancer 2003, 103(3):431-433.
18. Mahasneh AA, Abdel-Hafiz SS. Polymorphism of p53 gene in Jordanian population and possible associations with breast cancer and lung adenocarcinoma. Saudi Med J 2004, 25(11):1568-1573.
19. Menzel HJ, Sarmanova J, Soucek P, Berberich R, Grünewald K, Haun M, Kraft HG. Association of NQO1 polymorphism with spontaneous breast cancer in two independent populations. Br J Cancer 2004, 90(10):1989-1994.
20. Noma C, Miyoshi Y, Taguchi T, Tamaki Y, Noguchi S. Association of p53 genetic polymorphism (Arg72Pro) with estrogen receptor positive breast cancer risk in Japanese women. Cancer Lett 2004, 210(2):197-203.
21. Kalemi TG, Lambropoulos AF, Gueorguiev M, Chrisafi S, Papazisis KT, Kotsis A. The association of p53 mutations and p53 codon 72, Her 2 codon 655 and MTHFR C677T polymorphisms with breast cancer in Northern Greece. Cancer Lett 2005, 222(1):57-65.
22. Ohayon T, Gershoni-Baruch R, Papa MZ, Distelman Menachem T, Eisenberg Barzilai S, Friedman E. The R72P P53 mutation is associated with familial breast cancer in Jewish women. Br J Cancer 2005, 92(6):1144-1148.
23. Siddique MM, Balram C, Fiszer-Maliszewska L, Aggarwal A, Tan A, Tan P, Soo KC, Sabapathy K. Evidence for selective expression of the p53 codon 72 polymorphs: implications in cancer development. Cancer Epidemiol Biomarkers Prev 2005, 14(9):2245-2252.
24. Tommiska J, Eerola H, Heinonen M, Salonen L, Kaare M, Tallila J, Ristimäki A, von Smitten K, Aittomäki K, Heikkilä P *et al*. Breast cancer patients with p53 Pro72 homozygous genotype have a poorer survival. Clin Cancer Res 2005, 11(14):5098-5103.
25. Commonly studied single-nucleotide polymorphisms and breast cancer: results from the Breast Cancer Association Consortium. J Natl Cancer Inst 2006, 98(19):1382-1396.
26. Damin AP, Frazzon AP, Damin DC, Roehe A, Hermes V, Zettler C, Alexandre CO. Evidence for an association of TP53 codon 72 polymorphism with breast cancer risk. Cancer Detect Prev 2006, 30(6):523-529.
27. Ma H, Hu Z, Zhai X, Wang S, Wang X, Qin J, Chen W, Jin G, Liu J, Gao J *et al*. Joint effects of single nucleotide polymorphisms in P53BP1 and p53 on breast cancer risk in a Chinese population. Carcinogenesis 2006, 27(4):766-771.
28. Wirtenberger M, Frank B, Hemminki K, Klaes R, Schmutzler RK, Wappenschmidt B, Meindl A, Kiechle M, Arnold N, Weber BH *et al*. Interaction of Werner and Bloom syndrome genes with p53 in familial breast cancer. Carcinogenesis 2006, 27(8):1655-1660.
29. Baynes C, Healey CS, Pooley KA, Scollen S, Luben RN, Thompson DJ, Pharoah PD, Easton DF, Ponder BA, Dunning AM. Common variants in the ATM, BRCA1, BRCA2, CHEK2 and TP53 cancer susceptibility genes are unlikely to increase breast cancer risk. Breast Cancer Res 2007, 9(2):R27.
30. Buyru N, Altinisik J, Demokan S, Dalay N. p53 genotypes and haplotypes associated with risk of breast cancer. Cancer Detect Prev 2007, 31(3):207-213.
31. Cox DG, Deer D, Guo Q, Tworoger SS, Hankinson SE, Hunter DJ, De Vivo I. The p53 Arg72Pro and MDM2 -309 polymorphisms and risk of breast cancer in the nurses' health studies. Cancer Causes Control 2007, 18(6):621-625.
32. Franeková M, Zúbor P, Stanclová A, Dussan CA, Bohusová T, Galo S, Dobrota D, Kajo K, Péc M, Racay P. Association of p53 polymorphisms with breast cancer: a case-control study in Slovak population. Neoplasma 2007, 54(2):155-161.
33. Garcia-Closas M, Kristensen V, Langerød A, Qi Y, Yeager M, Burdett L, Welch R, Lissowska J, Peplonska B, Brinton L *et al*. Common genetic variation in TP53 and its flanking genes, WDR79 and ATP1B2, and susceptibility to breast cancer. Int J Cancer 2007, 121(11):2532-2538.
34. Gochhait S, Bukhari SI, Bairwa N, Vadhera S, Darvishi K, Raish M, Gupta P, Husain SA, Bamezai RN. Implication of BRCA2 -26G>A 5' untranslated region polymorphism in susceptibility to sporadic breast cancer and its modulation by p53 codon 72 Arg>Pro polymorphism. Breast Cancer Res 2007, 9(5):R71.
35. Johnson N, Fletcher O, Palles C, Rudd M, Webb E, Sellick G, dos Santos Silva I, McCormack V, Gibson L, Fraser A *et al*. Counting potentially functional variants in BRCA1, BRCA2 and ATM predicts breast cancer susceptibility. Hum Mol Genet 2007, 16(9):1051-1057.
36. Khadang B, Fattahi MJ, Talei A, Dehaghani AS, Ghaderi A. Polymorphism of TP53 codon 72 showed no association with breast cancer in Iranian women. Cancer Genet Cytogenet 2007, 173(1):38-42.
37. Pharoah PD, Tyrer J, Dunning AM, Easton DF, Ponder BA. Association between common variation in 120 candidate genes and breast cancer risk. PLoS Genet 2007, 3(3):e42.
38. Samson M, Swaminathan R, Rama R, Sridevi V, Nancy KN, Rajkumar T. Role of GSTM1 (Null/Present), GSTP1 (Ile105Val) and P53 (Arg72Pro) genetic polymorphisms and the risk of breast cancer: a case control study from South India. Asian Pac J Cancer Prev 2007, 8(2):253-257.
39. Schmidt MK, Reincke S, Broeks A, Braaf LM, Hogervorst FB, Tollenaar RA, Johnson N, Fletcher O, Peto J, Tommiska J *et al*. Do MDM2 SNP309 and TP53 R72P interact in breast cancer susceptibility? A large pooled series from the breast cancer association consortium. Cancer Res 2007, 67(19):9584-9590.
40. Sprague BL, Trentham-Dietz A, Garcia-Closas M, Newcomb PA, Titus-Ernstoff L, Hampton JM, Chanock SJ, Haines JL, Egan KM. Genetic variation in TP53 and risk of breast cancer in a population-based case control study. Carcinogenesis 2007, 28(8):1680-1686.
41. Zhang W. A case-control study on the relationship between polymorphisms of G _ 1mer S phase related regulatory genes and breast cancer susceptibility. Zhejiang University; 2007.
42. Cavallone L, Arcand SL, Maugard C, Ghadirian P, Mes-Masson AM, Provencher D, Tonin PN. Haplotype analysis of TP53 polymorphisms, Arg72Pro and Ins16, in BRCA1 and BRCA2 mutation carriers of French Canadian descent. BMC Cancer 2008, 8:96.
43. Costa S, Pinto D, Pereira D, Rodrigues H, Cameselle-Teijeiro J, Medeiros R, Schmitt F. Importance of TP53 codon 72 and intron 3 duplication 16bp polymorphisms in prediction of susceptibility on breast cancer. BMC Cancer 2008, 8:32.
44. De Vecchi G, Verderio P, Pizzamiglio S, Manoukian S, Bernard L, Pensotti V, Volorio S, Ravagnani F, Radice P, Peterlongo P. The p53 Arg72Pro and Ins16bp polymorphisms and their haplotypes are not associated with breast cancer risk in BRCA-mutation negative familial cases. Cancer Detect Prev 2008, 32(2):140-143.
45. Gaudet MM, Gammon MD, Bensen JT, Sagiv SK, Shantakumar S, Teitelbaum SL, Eng SM, Neugut AI, Santella RM. Genetic variation of TP53, polycyclic aromatic hydrocarbon-related exposures, and breast cancer risk among women on Long Island, New York. Breast Cancer Res Treat 2008, 108(1):93-99.
46. Lum SS, Chua HW, Li H, Li WF, Rao N, Wei J, Shao Z, Sabapathy K. MDM2 SNP309 G allele increases risk but the T allele is associated with earlier onset age of sporadic breast cancers in the Chinese population. Carcinogenesis 2008, 29(4):754-761.
47. Nordgard SH, Alnaes GI, Hihn B, Lingjaerde OC, Liestøl K, Tsalenko A, Sørlie T, Lønning PE, Børresen-Dale AL, Kristensen VN. Pathway based analysis of SNPs with relevance to 5-FU therapy: relation to intratumoral mRNA expression and survival. Int J Cancer 2008, 123(3):577-585.
48. Rajkumar T, Samson M, Rama R, Sridevi V, Mahji U, Swaminathan R, Nancy NK. TGFbeta1 (Leu10Pro), p53 (Arg72Pro) can predict for increased risk for breast cancer in south Indian women and TGFbeta1 Pro (Leu10Pro) allele predicts response to neo-adjuvant chemo-radiotherapy. Breast Cancer Res Treat 2008, 112(1):81-87.
49. Singh V, Rastogi N, Mathur N, Singh K, Singh MP. Association of polymorphism in MDM-2 and p53 genes with breast cancer risk in Indian women. Ann Epidemiol 2008, 18(1):48-57.
50. Akkiprik M, Sonmez O, Gulluoglu BM, Caglar HB, Kaya H, Demirkalem P, Abacioglu U, Sengoz M, Sav A, Ozer A. Analysis of p53 gene polymorphisms and protein over-expression in patients with breast cancer. Pathol Oncol Res 2009, 15(3):359-368.
51. Aoki MN, da Silva do Amaral Herrera AC, Amarante MK, do Val Carneiro JL, Fungaro MH, Watanabe MA. CCR5 and p53 codon 72 gene polymorphisms: implications in breast cancer development. Int J Mol Med 2009, 23(3):429-435.
52. Denisov EV, Cherdyntseva NV, Litvyakov NV, Slonimskaya EM, Malinovskaya EA, Voevoda MI, Belyavskaya VA, Stegniy VN. TP53 mutations and Arg72Pro polymorphism in breast cancers. Cancer Genet Cytogenet 2009, 192(2):93-95.
53. Henríquez-Hernández LA, Murias-Rosales A, Hernández González A, Cabrera De León A, Díaz-Chico BN, Mori De Santiago M, Fernández Pérez L. Gene polymorphisms in TYMS, MTHFR, p53 and MDR1 as risk factors for breast cancer: a case-control study. Oncol Rep 2009, 22(6):1425-1433.
54. Hrstka R, Beranek M, Klocova K, Nenutil R, Vojtesek B. Intronic polymorphisms in TP53 indicate lymph node metastasis in breast cancer. Oncol Rep 2009, 22(5):1205-1211.
55. Kazemi M, Salehi Z, Chakosari RJ. TP53 codon 72 polymorphism and breast cancer in northern Iran. Oncol Res 2009, 18(1):25-30.
56. Lång A, Palmebäck Wegman P, Wingren S. The significance of MDM2 SNP309 and p53 Arg72Pro in young women with breast cancer. Oncol Rep 2009, 22(3):575-579.
57. Ma HL, Zhu M, Huang TS. The p53(PIN3) gene polymorphism and susceptibility to breast cancer in Guizhou region women. Chin J Lab Diagn 2009(04):478-480.
58. Sinilnikova OM, Antoniou AC, Simard J, Healey S, Léoné M, Sinnett D, Spurdle AB, Beesley J, Chen X, Greene MH *et al*. The TP53 Arg72Pro and MDM2 309G>T polymorphisms are not associated with breast cancer risk in BRCA1 and BRCA2 mutation carriers. Br J Cancer 2009, 101(8):1456-1460.
59. Song F, Zheng H, Liu B, Wei S, Dai H, Zhang L, Calin GA, Hao X, Wei Q, Zhang W *et al*. An miR-502-binding site single-nucleotide polymorphism in the 3'-untranslated region of the SET8 gene is associated with early age of breast cancer onset. Clin Cancer Res 2009, 15(19):6292-6300.
60. Bisof V, Salihović MP, Narancić NS, Skarić-Jurić T, Jakić-Razumović J, Janićijević B, Turek S, Rudan P. TP53 gene polymorphisms and breast cancer in Croatian women: a pilot study. Eur J Gynaecol Oncol 2010, 31(5):539-544.
61. Ebner F, Schremmer-Danninger E, Rehbock J. The role of TP53 and p21 gene polymorphisms in breast cancer biology in a well specified and characterized German cohort. J Cancer Res Clin Oncol 2010, 136(9):1369-1375.
62. Jakubowska A, Gronwald J, Menkiszak J, Górski B, Huzarski T, Byrski T, Tołoczko-Grabarek A, Gilbert M, Edler L, Zapatka M *et al*. BRCA1-associated breast and ovarian cancer risks in Poland: no association with commonly studied polymorphisms. Breast Cancer Res Treat 2010, 119(1):201-211.
63. Kara N, Karakus N, Ulusoy AN, Ozaslan C, Gungor B, Bagci H. P53 codon 72 and HER2 codon 655 polymorphisms in Turkish breast cancer patients. DNA Cell Biol 2010, 29(7):387-392.
64. Nan JN, Chen CJ, Hu GX, Jin Y. Relationship between p53 codon72 Polymorphism and Breast Cancer in Yanbian area. Medical Journal of Yanbian University 2010(04):243-246.
65. Syeed N, Sameer AS, Abdullah S, Husain SA, Siddiqi MA. A Case-Control Study of TP53 R72P Polymorphism in the Breast Cancer Patients of Ethnic Kashmiri Population. World J Oncol 2010, 1(6):236-241.
66. Trifa F, Karray-Chouayekh S, Mabrouk I, Baccouche S, Khabir A, Sellami-Boudawara T, Gargouri A, Mokdad-Gargouri R. Haplotype analysis of p53 polymorphisms: Arg72Pro, Ins16bp and G13964C in Tunisian patients with familial or sporadic breast cancer. Cancer Epidemiol 2010, 34(2):184-188.
67. Alawadi S, Ghabreau L, Alsaleh M, Abdulaziz Z, Rafeek M, Akil N, Alkhalaf M. P53 gene polymorphisms and breast cancer risk in Arab women. Med Oncol 2011, 28(3):709-715.
68. Faghani M, Ghasemi FM, Nikhbakht M, Salehi M. TP53 PIN3 polymorphism associated with breast cancer risk in Iranian women. Indian J Cancer 2011, 48(3):298-302.
69. Koh WP, Van Den Berg D, Jin A, Wang R, Yuan JM, Yu MC. Combined effects of MDM2 SNP309 and TP53 R72P polymorphisms, and soy isoflavones on breast cancer risk among Chinese women in Singapore. Breast Cancer Res Treat 2011, 130(3):1011-1019.
70. Leu JD, Wang CY, Tsai HY, Lin IF, Chen RC, Lee YJ. Involvement of p53 R72P polymorphism in the association of MDM2-SNP309 with breast cancer. Oncol Rep 2011, 25(6):1755-1763.
71. Yoshimoto N, Nishiyama T, Toyama T, Takahashi S, Shiraki N, Sugiura H, Endo Y, Iwasa M, Fujii Y, Yamashita H. Genetic and environmental predictors, endogenous hormones and growth factors, and risk of estrogen receptor-positive breast cancer in Japanese women. Cancer Sci 2011, 102(11):2065-2072.
72. Alshatwi AA, Hasan TN, Shafi G, Alsaif MA, Al-Hazzani AA, Alsaif AA. A single-nucleotide polymorphism in the TP53 and MDM-2 gene modifies breast cancer risk in an ethnic Arab population. Fundam Clin Pharmacol 2012, 26(3):438-443.
73. Cherdyntseva NV, Denisov EV, Litviakov NV, Maksimov VN, Malinovskaya EA, Babyshkina NN, Slonimskaya EM, Voevoda MI, Choinzonov EL. Crosstalk between the FGFR2 and TP53 genes in breast cancer: data from an association study and epistatic interaction analysis. DNA Cell Biol 2012, 31(3):306-316.
74. Guleria K, Sharma S, Manjari M, Uppal MS, Singh NR, Sambyal V. p.R72P, PIN3 Ins16bp polymorphisms of TP53 and CCR5?32 in north Indian breast cancer patients. Asian Pac J Cancer Prev 2012, 13(7):3305-3311.
75. Proestling K, Hebar A, Pruckner N, Marton E, Vinatzer U, Schreiber M. The Pro allele of the p53 codon 72 polymorphism is associated with decreased intratumoral expression of BAX and p21, and increased breast cancer risk. PLoS One 2012, 7(10):e47325.
76. Lajin B, Alhaj Sakur A, Alachkar A. Association between polymorphisms in apoptotic genes and susceptibility for developing breast cancer in Syrian women. Breast Cancer Res Treat 2013, 138(2):611-619.
77. Liu J, Tang X, Li M, Lu C, Shi J, Zhou L, Yuan Q, Yang M. Functional MDM4 rs4245739 genetic variant, alone and in combination with P53 Arg72Pro polymorphism, contributes to breast cancer susceptibility. Breast Cancer Res Treat 2013, 140(1):151-157.
78. Rodrigues P, Furriol J, Tormo E, Ballester S, Lluch A, Eroles P. Epistatic interaction of Arg72Pro TP53 and -710 C/T VEGFR1 polymorphisms in breast cancer: predisposition and survival. Mol Cell Biochem 2013, 379(1-2):181-190.
79. Wang ML, Xu YX, Qian J, Wang FH. [Association of murine double minute 2 and P53 polymorphisms with breast cancer susceptibility]. Zhonghua Yu Fang Yi Xue Za Zhi 2013, 47(2):124-128.
80. Krivokuca AM, Malisic EJ, Dobricic JD, Brotto KV, Cavic MR, Jankovic RN, Tomasevic ZI, Brankovic-Magic MV. RAD51 135G>C and TP53 Arg72Pro polymorphisms and susceptibility to breast cancer in Serbian women. Fam Cancer 2014, 13(2):173-180.
81. Marouf C, Tazzite A, Diakité B, Jouhadi H, Benider A, Nadifi S. Association of TP53 PIN3 polymorphism with breast cancer in Moroccan population. Tumour Biol 2014, 35(12):12403-12408.
82. Pouladi N, Kouhsari SM, Feizi MH, Dehghan R, Azarfam P, Farajzadeh D. Lack of association of intron 3 16 bp polymorphism of TP53 with breast cancer among Iranian-Azeri patients. Asian Pac J Cancer Prev 2014, 15(6):2631-2634.
83. Saadatian Z, Gharesouran J, Ghojazadeh M, Ghohari-Lasaki S, Tarkesh-Esfahani N, Mohaddes Ardebili SM. Association of rs1219648 in FGFR2 and rs1042522 in TP53 with premenopausal breast cancer in an Iranian Azeri population. Asian Pac J Cancer Prev 2014, 15(18):7955-7958.
84. Sharma S, Sambyal V, Guleria K, Manjari M, Sudan M, Uppal MS, Singh NR, Bansal D, Gupta A. TP53 polymorphisms in sporadic North Indian breast cancer patients. Asian Pac J Cancer Prev 2014, 15(16):6871-6879.
85. Arfaoui A, Douik H, Kablouti G, Chaaben AB, Handiri N, Zid Z, Ouni N, Zouiouch F, Ayari F, Mamoughli T *et al*. Role of p53 Codon72 SNP in breast cancer risk and anthracycline resistance. Anticancer Res 2015, 35(3):1763-1769.
86. Devi KR, Chenkual S, Majumdar G, Ahmed J, Kaur T, Zonunmawia JC, Mukherjee K, Phukan RK, Mahanta J, Rajguru SK *et al*. TLR2∆22 (-196-174) significantly increases the risk of breast cancer in females carrying proline allele at codon 72 of TP53 gene: a case-control study from four ethnic groups of North Eastern region of India. Tumour Biol 2015, 36(12):9995-10002.
87. Eskandari-Nasab E, Hashemi M, Amininia S, Ebrahimi M, Rezaei M, Hashemi SM. Effect of TP53 16-bp and β-TrCP 9-bp INS/DEL polymorphisms in relation to risk of breast cancer. Gene 2015, 568(2):181-185.
88. Gohari-Lasaki S, Gharesouran J, Ghojazadeh M, Montazeri V, Mohaddes Ardebili SM. Lack of influence of TP53 Arg72Pro and 16bp duplication polymorphisms on risk of breast cancer in Iran. Asian Pac J Cancer Prev 2015, 16(7):2971-2974.
89. Vymetalkova V, Soucek P, Kunicka T, Jiraskova K, Brynychova V, Pardini B, Novosadova V, Polivkova Z, Kubackova K, Kozevnikovova R *et al*. Genotype and Haplotype Analyses of TP53 Gene in Breast Cancer Patients: Association with Risk and Clinical Outcomes. PLoS One 2015, 10(7):e0134463.
90. Almeida BC, Kleine JP, Camargo-Kosugi CM, Lisboa MR, França CN, França JP, Silva ID. Analysis of polymorphisms in codons 11, 72 and 248 of TP53 in Brazilian women with breast cancer. Genet Mol Res 2016, 15(1).
91. Golmohammadi R, Namazi MJ. Allelic polymorphism in codon 72 of p53 gene: prognosis value, survival rates, and their association with breast cancer. Eur J Gynaecol Oncol 2016, 37(4):493-498.
92. Shabnaz S, Ahmed MU, Islam MS, Islam MR, Al-Mamun MM, Islam MS, Hasnat A. Breast cancer risk in relation to TP53 codon 72 and CDH1 gene polymorphisms in the Bangladeshi women. Tumour Biol 2016, 37(6):7229-7237.
93. Yadav P, Masroor M, Tanwer K, Mir R, Javid J, Ahmad I, Zuberi M, Kaza RC, Jain SK, Khurana N *et al*. Clinical significance of TP53 (R72P) and MDM2 (T309G) polymorphisms in breast cancer patients. Clin Transl Oncol 2016, 18(7):728-734.
94. Hossain A, Murshid GMM, Zilani MNH, Islam F, Sultana R, Sultana T, Hossain MG, Rahman MM. TP53 codon 72 polymorphism and breast cancer risk in Bangladeshi population. Breast Cancer 2017, 24(4):571-578.
95. Isakova J, Talaibekova E, Aldasheva N, Vinnikov D, Aldashev A. The association of polymorphic markers Arg399Gln of XRCC1 gene, Arg72Pro of TP53 gene and T309G of MDM2 gene with breast cancer in Kyrgyz females. BMC Cancer 2017, 17(1):758.
96. Akhter N, Dar SA, Chattopadhyay S, Haque S, Anwer R, Wahid M, Jawed A, Lohani M, Mandal RK, Shukla NK *et al*. Impact of p53 arg72pro SNP on Breast Cancer Risk in North Indian Population. Curr Genomics 2018, 19(5):395-410.
97. Ayoubi SE, Elkarroumi M, El Khachibi M, Hassani Idrissi H, Ayoubi H, Ennachit S, Arazzakou M, Nadifi S. The 72Pro Variant of the Tumor Protein 53 Is Associated with an Increased Breast Cancer Risk in the Moroccan Population. Pathobiology 2018, 85(4):247-253.
98. Chen FY, Wang H, Li H, Hu XL, Dai X, Wang SM, Yan GJ, Jiang PL, Hu YP, Huang J *et al*. Association of Single-Nucleotide Polymorphisms in Monoubiquitinated FANCD2-DNA Damage Repair Pathway Genes With Breast Cancer in the Chinese Population. Technol Cancer Res Treat 2018, 17:1533033818819841.
99. Habyarimana T, Attaleb M, Mugenzi P, Mazarati JB, Bakri Y, El Mzibri M. Association of p53 Codon 72 Polymorphism with Breast Cancer in a Rwandese Population. Pathobiology 2018, 85(3):186-191.
100. Hao W, Xu X, Shi H, Zhang C, Chen X. No association of TP53 codon 72 and intron 3 16-bp duplication polymorphisms with breast cancer risk in Chinese Han women: new evidence from a population-based case-control investigation. Eur J Med Res 2018, 23(1):47.
101. Assad Samani L, Javadirad SM, Parsafar S, Tabatabaeian H, Ghaedi K, Azadeh M. TP53 rs1625895 is Related to Breast Cancer Incidence and Early Death in Iranian Population. Indian J Clin Biochem 2019, 34(4):485-489.
102. Morten BC, Chiu S, Oldmeadow C, Lubinski J, Scott RJ, Avery-Kiejda KA. The intron 3 16 bp duplication polymorphism of p53 (rs17878362) is not associated with increased risk of developing triple-negative breast cancer. Breast Cancer Res Treat 2019, 173(3):727-733.
103. Afzaljavan F, Chaeichi Tehrani N, Rivandi M, Zarif Ghasemian S, Vahednia E, Khayami R, Abavisani M, Pasdar A. The Dilemma of TP53 Codon 72 Polymorphism (rs1042522) and Breast Cancer Risk: A Case-Control Study and Meta-Analysis in The Iranian Population. Cell J 2020, 22(2):185-192.
104. Diakite B, Kassogue Y, Dolo G, Kassogue O, Keita ML, Joyce B, Neuschler E, Wang J, Musa J, Traore CB *et al*. Association of PIN3 16-bp duplication polymorphism of TP53 with breast cancer risk in Mali and a meta-analysis. BMC Med Genet 2020, 21(1):142.
105. Icen-Taskin I, Irtegun-Kandemir S, Munzuroglu O. TP53 rs1042522 polymorphism and early-onset breast cancer. J Res Med Sci 2020, 25:25.
106. Isakova JT, Vinnikov D, Kipen VN, Talaibekova ET, Aldashev AA, Aldasheva NM, Makieva KB, Semetei Kyzy A, Bukuev NM, Tilekov EA *et al*. Gene-to-gene interactions and the association of TP53, XRCC1, TNFα, HMMR, MDM2 and PALB2 with breast cancer in Kyrgyz females. Breast Cancer 2020, 27(5):938-946.
107. Pouladi N, Shavali M, Abdolahi S. Combined Genotype Effects of TP53 and PAI-1 Polymorphisms in Breast Cancer Susceptibility: Multifactor Dimensionality Reduction and in silico Analysis. Hum Hered 2020, 85(2):51-60.
108. Akhter N, Dar SA, Haque S, Wahid M, Jawed A, Akhtar MS, Alharbi RA, Sindi AAA, Alruwetei A, Zubair Choudhry HM *et al*. Crosstalk of Cyclin-dependent kinase inhibitor 1A (CDKN1A) gene polymorphism with p53 and CCND1 polymorphism in breast cancer. Eur Rev Med Pharmacol Sci 2021, 25(12):4258-4273.
